# Supplementary material for: Design, Synthesis, and Biological Evaluation of N′-Phenylhydrazides as Potential Antifungal Agents
Source: Int J Mol Sci. 2023 Oct 12;24(20):15120. doi: 10.3390/ijms242015120 (PMC10606473; doi:10.3390/ijms242015120)
Supplement: Supplementary file 1 [file ijms-24-15120-s001.zip › ijms-2580181-supplementary.pdf]

Design, Synthesis, and Biological Evaluation of *N*'-Phenylhydrazides as Potential Antifungal Agents

Supporting information

1. Characterizations of target compounds·······················S1

2. The spectra of all synthesized compounds·················S15

References···S78

1. Characterizations of target compounds

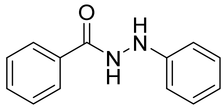

N'-phenylbenzohydrazide (**A1**). Yield: 96%; white crystal; m.p. 153.0–153.9 °C; ^{1}H NMR (400 MHz, DMSO-d_6): δ 10.36 (d, J = 3.0 Hz, 1H), 7.93 (s, 1H), 7.91 (d, J = 1.6 Hz, 1H), 7.90 (d, J = 3.1 Hz, 1H), 7.61 – 7.54 (m, 1H), 7.50 (t, J = 7.3 Hz, 2H), 7.15 (dd, J = 8.5, 7.2 Hz, 2H), 6.79 (d, J = 7.5 Hz, 2H), 6.72 (t, J = 7.3 Hz, 1H); ^{13}C NMR (100 MHz, DMSO-d_6): δ 166.8, 150.0, 133.5, 132.1, 129.2, 128.9, 127.7, 119.1, 112.8. This is a known compound and the spectral data are identical to those reported in the literature [1].

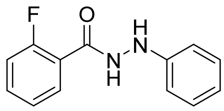

2-fluoro-N'-phenylbenzohydrazide (**A2**). Yield: 65%; white crystal; m.p. 131.2–133.6 °C; ^{1}H NMR (400 MHz, DMSO-d_6): δ 10.19 (s, 1H), 7.65 (td, J = 7.4, 1.8 Hz, 1H), 7.61 – 7.54 (m, 1H), 7.39 – 7.27 (m, 2H), 7.22 – 7.15 (m, 2H), 6.82 (d, J = 7.6 Hz, 2H), 6.73 (t, J = 7.3 Hz, 1H). ^{13}C NMR (101 MHz, DMSO-d_6) δ 164.54, 160.88, 158.41, 149.60, 133.14 (d, J = 8.3 Hz), 130.46 (d, J = 3.2 Hz), 129.25, 125.10 (d, J = 3.5 Hz), 123.47 (d, J = 15.5 Hz), 116.66 (d, J = 21.9 Hz), 112.73. This is a known compound and the spectral data are identical to those reported in the literature [2].

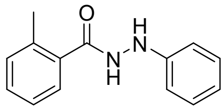

2-methyl-N'-phenylbenzohydrazide (**A3**). Yield: 69%; white crystal; m.p. 163.3–164.7 °C; ^{1}H NMR (400 MHz, DMSO-d_6): δ 10.06 (d, J = 3.0 Hz, 1H), 7.94 (d, J = 3.0 Hz, 1H), 7.46 (d, J = 7.5 Hz, 1H), 7.37 (t, J = 7.4 Hz, 1H), 7.31 – 7.26 (m, 2H), 7.18 (t, J = 7.7 Hz, 2H), 6.82 (d, J = 8.1 Hz, 2H), 6.73 (t, J = 7.2 Hz, 1H), 2.38 (s, 3H); ^{13}C NMR (100 MHz, DMSO-d_6): δ 169.4, 149.9, 136.1, 135.6, 131.0, 130.2, 129.2, 127.7, 126.1, 119.0, 112.7, 19.8. HRMS (ESI, positive) *m/z*: calcd for $C_{14}H_{15}N_2O^+$ [M+H]$^+$, 227.1179, found, 227.1184. This is a known compound and the spectral data are identical to those reported in the literature [3].

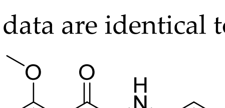

2-methoxy-N'-phenylbenzohydrazide (**A4**). Yield: 65%; white crystal; m.p. 80.6–81.8 °C; ^{1}H NMR (400 MHz, DMSO-d_6): δ 9.85 (s, 1H), 7.64 (d, J = 7.5 Hz, 1H), 7.50 (t, J = 7.8 Hz, 1H), 7.21 – 7.15 (m, 3H), 7.06 (t, J = 7.5 Hz, 1H), 6.85 (d, J = 8.1 Hz, 2H), 6.74 (t, J = 7.2 Hz, 1H), 3.91 (s, 3H); ^{13}C NMR (100 MHz, DMSO-d_6): δ 166.3, 157.3, 149.9, 132.5, 130.3, 129.1, 123.5, 120.9, 118.9,

112.8, 112.3, 56.2. This is a known compound and the spectral data are identical to those reported in the literature [2].

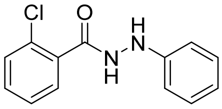

2-chloro-N'-phenylbenzohydrazide (**A5**). Yield: 62%; white crystal; m.p. 150.1–150.8 °C; ^{1}H NMR (400 MHz, DMSO-*d*$_6$): *δ* 10.21 (d, *J* = 2.0 Hz, 1H), 8.02 (s, 1H), 7.59 – 7.38 (m, 4H), 7.18 (t, *J* = 7.7 Hz, 2H), 6.86 (d, *J* = 7.9 Hz, 2H), 6.73 (t, *J* = 7.1 Hz, 1H); ^{13}C NMR (100 MHz, DMSO-*d*$_6$): *δ* 166.7, 149.6, 135.7, 131.7, 130.7, 130.2, 129.6, 129.2, 127.7, 119.1, 112.8. HRMS (ESI, positive) *m*/*z*: calcd for $C_{13}H_{12}ClN_2O^+$ [M+H]$^+$, 247.0633, found, 247.0639. This is a known compound and the spectral data are identical to those reported in the literature [4].

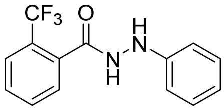

N'-phenyl-2-(trifluoromethyl)benzohydrazide (**A6**). Yield: 55%; White flocculent solid; m.p. 149.9–151.1 °C; ^{1}H NMR (400 MHz, DMSO-*d*$_6$): *δ* 10.47 (s, 1H), 7.97 (s, 1H), 7.79 (d, *J* = 7.8 Hz, 1H), 7.73 (ddd, *J* = 9.9, 2.6, 1.5 Hz, 1H), 7.57 (td, *J* = 8.0, 5.8 Hz, 1H), 7.44 (td, *J* = 8.3, 1.7 Hz, 1H), 7.16 (dd, *J* = 8.6, 7.2 Hz, 2H), 6.81 (s, 2H), 6.73 (t, *J* = 7.3 Hz, 1H). ^{13}C NMR (101 MHz, DMSO-*d*$_6$) δ 167.35, 149.62, 135.10 (d, *J* = 2.3 Hz), 133.06, 131.65, 130.76, 130.14, 129.35 (d, *J* = 26.9 Hz), 126.94 (d, *J* = 5.3 Hz), 122.82, 119.16, 112.69. HRMS (ESI, positive) *m*/*z*: calcd for $C_{14}H_{15}N_2O^+$ [M+H]$^+$, 281.0896, found, 281.0894.

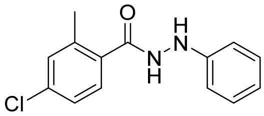

4-chloro-2-methyl-N'-phenylbenzohydrazide (**A7**). Yield: 66%; White flocculent solid; m.p. 190.8–191.5 °C; ^{1}H NMR (400 MHz, DMSO-*d*$_6$): *δ* 10.11 (d, *J* = 2.5 Hz, 1H), 7.94 (d, *J* = 2.7 Hz, 1H), 7.48 (d, *J* = 8.1 Hz, 1H), 7.40 (s, 1H), 7.37 (d, *J* = 8.1 Hz, 1H), 7.17 (t, *J* = 7.7 Hz, 2H), 6.81 (d, *J* = 8.2 Hz, 2H), 6.73 (t, *J* = 7.2 Hz, 1H), 2.38 (s, 3H); ^{13}C NMR (100 MHz, DMSO-*d*$_6$): *δ* 168.5, 149.7, 138.9, 134.7, 134.3, 130.7, 129.6, 129.2, 126.0, 119.1, 112.6, 19.5. HRMS (ESI, positive) *m*/*z*: calcd for $C_{14}H_{14}ClN_2O^+$ [M+H]$^+$, 261.0789, found, 261.0793. This is a known compound and the spectral data are identical to those reported in the literature [5].

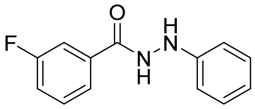

3-fluoro-N'-phenylbenzohydrazide (**A8**). Yield: 75%; white crystal; m.p. 150.3–150.8 °C; ^{1}H NMR (400 MHz, DMSO-*d*$_6$): *δ* 10.48 (d, *J* = 3.0 Hz, 1H), 7.97 (d, *J* = 2.9 Hz, 1H), 7.79 (d, *J* = 7.7

Hz, 1H), 7.73 (dt, *J* = 9.7, 2.2 Hz, 1H), 7.57 (td, *J* = 8.0, 5.8 Hz, 1H), 7.44 (td, *J* = 8.5, 2.6 Hz, 1H), 7.16 (dd, *J* = 8.5, 7.2 Hz, 2H), 6.84 – 6.77 (m, 2H), 6.73 (t, *J* = 7.3 Hz, 1H); ^{13}C NMR (100 MHz, DMSO-d_6): *δ* 165.5 (d, *J* = 2.6 Hz), 162.4 (d, *J* = 244.7 Hz), 149.8, 135.7 (d, *J* = 6.6 Hz), 131.2 (d, *J* = 8.0 Hz), 129.2, 123.9 (d, *J* = 3.1 Hz), 119.2, 119.0 (d, *J* = 21.1 Hz), 114.6 (d, *J* = 22.8 Hz), 112.8. This is a known compound and the spectral data are identical to those reported in the literature [6].

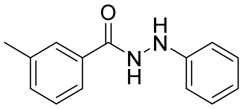

3-methyl-N'-phenylbenzohydrazide (**A$_9$**). Yield: 74%; white crystal; m.p. 155.9–157.1 °C; ^{1}H NMR (400 MHz, DMSO-d_6): *δ* 10.33 (d, *J* = 2.5 Hz, 1H), 7.92 (d, *J* = 2.5 Hz, 1H), 7.75 (s, 1H), 7.74 – 7.69 (m, 1H), 7.38 (d, *J* = 4.6 Hz, 2H), 7.15 (t, *J* = 7.7 Hz, 2H), 6.79 (d, *J* = 8.0 Hz, 2H), 6.72 (t, *J* = 7.2 Hz, 1H); ^{13}C NMR (100 MHz, DMSO-d_6): *δ* 166.9, 150.0, 138.2, 133.5, 132.66, 129.2, 128.8, 128.3, 124.8, 119.0, 112.7, 21.4. This is a known compound and the spectral data are identical to those reported in the literature [6].

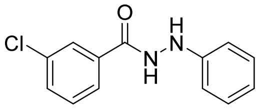

3-chloro-N'-phenylbenzohydrazide (**A$_{10}$**). Yield:75%; white crystal; m.p. 152.9–153.1 °C; ^{1}H NMR (400 MHz, DMSO-d_6): *δ* 10.50 (s, 1H), 7.97 (d, *J* = 8.8 Hz, 2H), 7.89 (d, *J* = 7.8 Hz, 1H), 7.67 (d, *J* = 8.0 Hz, 1H), 7.56 (t, *J* = 7.8 Hz, 1H), 7.16 (t, *J* = 7.9 Hz, 2H), 6.79 (d, *J* = 8.0 Hz, 2H), 6.73 (t, *J* = 7.3 Hz, 1H); ^{13}C NMR (100 MHz, DMSO-d_6): *δ* 165.4, 149.7, 135.4, 133.8, 132.02, 131.0, 129.2, 127.6, 126.5, 119.2, 112.8. HRMS (ESI, positive) *m/z*: calcd for $C_{13}H_{12}ClN_2O^+$ [M+H]$^+$, 247.0633, found, 247.0632. This is a known compound and the spectral data are identical to those reported in the literature [5].

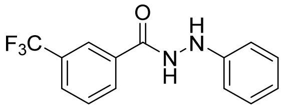

N'-phenyl-3-(trifluoromethyl)benzohydrazide (**A$_{11}$**). Yield: 59%; white flocculent solid; m.p. 110.7–112.1 °C; ^{1}H NMR (400 MHz, DMSO-d_6): *δ* 10.64 (s, 1H), 8.29 – 8.21 (m, 2H), 7.97 (d, *J* = 8.5 Hz, 1H), 7.78 (t, *J* = 7.8 Hz, 1H), 7.17 (dd, *J* = 8.6, 7.2 Hz, 2H), 6.82 (d, *J* = 7.4 Hz, 2H), 6.74 (t, *J* = 7.3 Hz, 1H). ^{13}C NMR (101 MHz, DMSO-d_6) δ 165.38, 149.72, 134.35, 131.87, 130.31, 129.83 (d, *J* = 31.9 Hz), 129.24, 128.69 (d, *J* = 3.6 Hz), 125.76, 124.41 (d, *J* = 4.0 Hz), 119.28, 112.87. It is a known compound and the spectral data are identical to those reported in the literature [7].

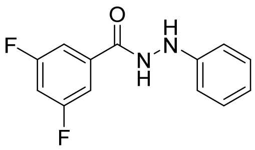

3,5-difluoro-N'-phenylbenzohydrazide (**A12**). Yield: 58%; white solid; mp. 147.5–148.3 °C; ^{1}H NMR (400 MHz, DMSO-d_6): δ 10.20 (s, 1H), 8.00 (d, J = 16.6 Hz, 1H), 7.66 (t, J = 7.2 Hz, 1H), 7.56 (q, J = 6.8, 6.4 Hz, 1H), 7.32 (q, J = 8.1, 7.0 Hz, 2H), 7.18 (t, J = 7.7 Hz, 2H), 6.83 (d, J = 8.0 Hz, 2H), 6.74 (t, J = 7.3 Hz, 1H); ^{13}C NMR (100 MHz, DMSO-d_6): δ 164.5, 159.6 (d, J = 249.2 Hz), 149.6, 133.1 (d, J = 8.2 Hz), 130.4 (d, J = 3.0 Hz), 129.2, 125.1 (d, J = 3.5 Hz), 123.4 (d, J = 15.4 Hz), 119.1, 116.6 (d, J = 21.9 Hz), 112.7. HRMS (ESI, positive) m/z: calcd for $C_{13}H_{10}F_2N_2O^+$ [M+H]$^+$, 249.0834, found, 249.0830.

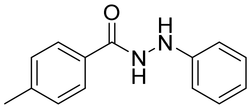

4-methyl-N'-phenylbenzohydrazide (**A13**). Yield: 78%; white solid; m.p. 161.4–162.1 °C; ^{1}H NMR (400 MHz, DMSO-d_6): δ 10.29 (d, J = 3.0 Hz, 1H), 7.88 (d, J = 2.9 Hz, 1H), 7.83 (d, J = 7.9 Hz, 2H), 7.31 (d, J = 7.9 Hz, 2H), 7.15 (t, J = 7.7 Hz, 2H), 6.78 (d, J = 8.0 Hz, 3H), 6.71 (t, J = 7.3 Hz, 1H), 2.37 (s, 3H); ^{13}C NMR (100 MHz, DMSO-d_6): δ 166.6, 150.0, 142.0, 130.6, 129.4, 129.2, 127.7, 119.0, 112.7, 21.4. This is a known compound and the spectral data are identical to those reported in the literature [8].

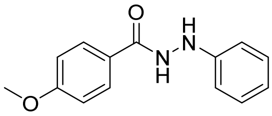

4-methoxy-N'-phenylbenzohydrazide (**A14**). Yield: 73%; light yellow solid; m.p. 169.9–170.9 °C; ^{1}H NMR (400 MHz, DMSO-d_6): δ 10.23 (d, J = 3.0 Hz, 1H), 7.91 (d, J = 8.7 Hz, 2H), 7.85 (d, J = 2.9 Hz, 1H), 7.14 (t, J = 7.7 Hz, 2H), 7.03 (d, J = 8.8 Hz, 2H), 6.77 (d, J = 8.0 Hz, 2H), 6.71 (t, J = 7.2 Hz, 1H), 3.82 (s, 3H); ^{13}C NMR (100 MHz, DMSO-d_6): δ 166.2, 162.3, 150.1, 129.6, 129.1, 125.6, 119.0, 114.1, 112.7, 55.8. This is a known compound and the spectral data are identical to those reported in the literature [1].

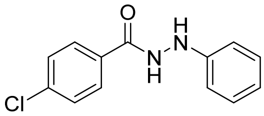

4-chloro-N'-phenylbenzohydrazide (**A15**). Yield: 83%; white crystal; m.p. 186.5–187.4 °C; ^{1}H NMR (400 MHz, DMSO-d_6): δ 10.46 (d, J = 2.9 Hz, 1H), 8.02 – 7.89 (m, 3H), 7.59 (d, J = 8.5 Hz, 2H), 7.22 – 7.10 (m, 2H), 6.79 (d, J = 7.9 Hz, 2H), 6.73 (t, J = 7.3 Hz, 1H); ^{13}C NMR (100 MHz, DMSO-d_6): δ 165.8, 149.8, 136.9, 132.2, 129.7, 129.2, 129.0, 119.1, 112.8. This is a known

compound and the spectral data are identical to those reported in the literature [9].

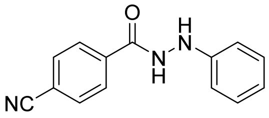

4-cyano-N'-phenylbenzohydrazide (**A$_{16}$**). Yield: 61%; light yellow solid; m.p. 165.8–166.3 °C; ^{1}H NMR (400 MHz, DMSO-d_6): δ 10.64 (s, 1H), 8.11 – 7.99 (m, 5H), 7.17 (t, J = 7.7 Hz, 2H), 6.81 (d, J = 8.0 Hz, 2H), 6.74 (t, J = 7.3 Hz, 1H); ^{13}C NMR (100 MHz, DMSO): δ 165.4, 149.6, 137.5, 133.0, 129.2, 128.6, 119.3, 118.7, 114.5, 112.8. This is a known compound and the spectral data are identical to those reported in the literature [1].

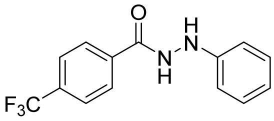

N'-phenyl-4-(trifluoromethyl)benzohydrazide (**A$_{17}$**). Yield: 64%; white crystal; m.p. 186.5–187.5 °C; ^{1}H NMR (400 MHz, DMSO-d_6): δ 10.63 (d, J = 2.3 Hz, 1H), 8.15 (d, J = 8.1 Hz, 2H), 8.04 (d, J = 2.3 Hz, 1H), 7.90 (d, J = 8.2 Hz, 2H), 7.18 (t, J = 7.7 Hz, 2H), 6.83 (d, J = 8.1 Hz, 2H), 6.75 (t, J = 7.3 Hz, 1H); ^{13}C NMR (100 MHz, DMSO-d_6): δ 165.7, 149.7, 137.3, 132.0 (q, J = 31.9 Hz), 129.2, 125.9 (q, J = 3.7 Hz), 124.3 (q, J = 272.5 Hz), 119.2, 112.8. HRMS (ESI, positive) *m/z*: calcd for $C_{14}H_{12}F_3N_2O^+$ $[M+H]^+$, 281.0902, found, 281.0896. This is a known compound and the spectral data are identical to those reported in the literature [10].

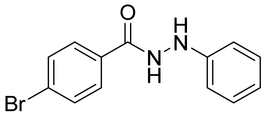

4-bromo-N'-phenylbenzohydrazide (**A$_{18}$**). Yield: 81%; white crystal; m.p. 193.3–194.3 °C; ^{1}H NMR (400 MHz, DMSO-d_6): δ 10.46 (d, J = 2.9 Hz, 1H), 7.95 (d, J = 2.9 Hz, 1H), 7.87 (d, J = 8.4 Hz, 2H), 7.77 – 7.69 (m, 2H), 7.16 (t, J = 7.8 Hz, 2H), 6.79 (d, J = 8.0 Hz, 2H), 6.73 (t, J = 7.2 Hz, 1H); ^{13}C NMR (100 MHz, DMSO-d_6): δ 165.9, 149.8, 132.6, 132.0, 129.9, 129.2, 125.8, 119.2, 112.8. This is a known compound and the spectral data are identical to those reported in the literature [1].

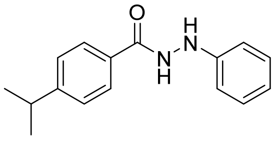

4-isopropyl-N'-phenylbenzohydrazide (**A$_{19}$**). Yield: 65%; white crystal; m.p. 197.0–197.4 °C; ^{1}H NMR (400 MHz, DMSO-d_6): δ 10.31 (s, 1H), 7.87 (d, J = 7.9 Hz, 3H), 7.37 (d, J = 7.9 Hz, 2H), 7.15 (t, J = 7.6 Hz, 2H), 6.78 (d, J = 8.0 Hz, 2H), 6.71 (t, J = 7.2 Hz, 1H), 2.95 (hept, J = 6.9 Hz, 1H), 1.23 (s, 3H), 1.22 (s, 3H); ^{13}C NMR (100 MHz, DMSO-d_6): δ 166.7, 152.7, 150.0, 131.1, 129.1,

127.9, 126.8, 119.0, 112.7, 33.8, 24.1. HRMS (ESI, positive) *m/z*: calcd for $C_{16}H_{18}N_2O^+$ [M+H]$^+$, 255.1497, found: 255.1493 This is a known compound and the spectral data are identical to those reported in the literature [5].

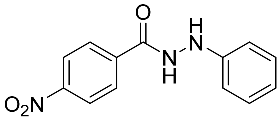

4-nitro-N'-phenylbenzohydrazide (**A20**). Yield: 60%; yellow solid; m.p. 197.4–198.1 °C; ^{1}H NMR (400 MHz, DMSO-d_6): δ 10.70 (d, *J* = 2.8 Hz, 1H), 8.36 (d, *J* = 8.8 Hz, 2H), 8.15 (d, *J* = 8.8 Hz, 2H), 8.06 (d, *J* = 2.6 Hz, 1H), 7.17 (t, *J* = 7.9 Hz, 2H), 6.81 (d, *J* = 8.0 Hz, 2H), 6.74 (t, *J* = 7.3 Hz, 1H); ^{13}C NMR (100 MHz, DMSO-d_6): δ 165.2, 149.7, 149.5, 139.1, 129.3, 129.3, 124.1, 119.3, 112.8. This is a known compound and the spectral data are identical to those reported in the literature [1].

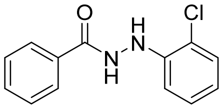

N'-(2-chlorophenyl)benzohydrazide (**B1**). Yield: 60%; white solid; m.p. 145.2–146.9 °C; ^{1}H NMR (400 MHz, DMSO-d_6): δ 10.53 (d, *J* = 1.8 Hz, 1H), 7.94 (d, *J* = 7.4 Hz, 2H), 7.63 (d, *J* = 1.7 Hz, 1H), 7.60 (t, *J* = 7.4 Hz, 1H), 7.52 (t, *J* = 7.5 Hz, 2H), 7.32 (d, *J* = 7.8 Hz, 1H), 7.16 (t, *J* = 7.7 Hz, 1H), 6.84 (d, *J* = 8.0 Hz, 1H), 6.77 (t, *J* = 7.6 Hz, 1H); ^{13}C NMR (100 MHz, DMSO-d_6): δ 166.7, 145.3, 133.1, 132.3, 129.6, 129.0, 128.2, 127.8, 120.1, 117.8, 113.4. This is a known compound and the spectral data are identical to those reported in the literature [1].

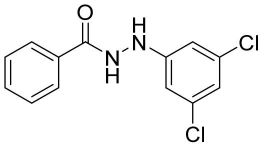

N'-(3,5-dichlorophenyl)benzohydrazide (**B2**). Yield: 60%; light powder flocculent solid; m.p. 183.2–184.1 °C; ^{1}H NMR (400 MHz, DMSO-d_6): δ 10.54 (s, 1H), 8.58 (s, 1H), 7.96 (d, *J* = 7.4 Hz, 2H), 7.61 (t, *J* = 7.2 Hz, 1H), 7.53 (t, *J* = 7.5 Hz, 3H), 6.86 (s, 1H), 6.77 (s, 2H); ^{13}C NMR (100 MHz, DMSO-d_6): δ 166.8, 152.4, 134.9, 132.8, 132.4, 129.0, 127.8, 117.8, 110.8. HRMS (ESI, positive) *m/z*: calcd for $C_{13}H_{11}C_{12}N_2O^+$ [M+H]$^+$, 281.0243, found, 281.0248. This is a known compound and the spectral data are identical to those reported in the literature [5].

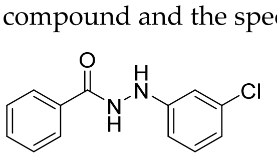

N'-(3-chlorophenyl)benzohydrazide (**B3**). Yield: 65%; Brown crystal; m.p. 137.8–141.1 °C; ^{1}H NMR (400 MHz, DMSO-d_6): δ 10.44 (s, 1H), 8.25 (s, 1H), 7.94 (d, *J* = 7.9 Hz, 2H), 7.59 (t, *J* = 7.3

Hz, 1H), 7.52 (t, *J* = 7.6 Hz, 2H), 7.17 (t, *J* = 7.9 Hz, 1H), 6.77 (d, *J* = 6.2 Hz, 2H), 6.74 (s, 1H); ^{13}C NMR (100 MHz, DMSO-d_6): *δ* 166.8, 151.6, 133.9, 133.1, 132.2, 130.9, 129.0, 127.8, 118.5, 112.0, 111.4. This is a known compound and the spectral data are identical to those reported in the literature [1].

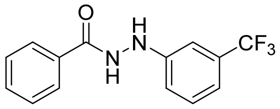

N'-(3-(trifluoromethyl)phenyl)benzohydrazide (**B**$_4$). Yield: 57%; brown solid; m.p. 167.7–168.5 ^{o}C; ^{1}H NMR (400 MHz, DMSO-d_6): *δ* 10.50 (d, *J* = 2.5 Hz, 1H), 8.41 (d, *J* = 2.5 Hz, 1H), 8.05 – 7.85 (m, 2H), 7.63 – 7.57 (m, 1H), 7.53 (t, *J* = 7.5 Hz, 2H), 7.39 (t, *J* = 7.8 Hz, 1H), 7.05 (d, *J* = 2.8 Hz, 1H), 7.03 (s, 2H); ^{13}C NMR (100 MHz, DMSO-d_6): *δ* 166.8, 150.6, 133.1, 132.3, 130.4, 130.1 (q, *J* = 31.3 Hz), 129.0, 127.8, 124.8 (q, *J* = 272.3 Hz), 116.2, 115.1 (q, *J* = 3.9 Hz), 108.5 (q, *J* = 4.1 Hz). This is a known compound and the spectral data are identical to those reported in the literature [11].

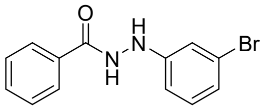

N'-(3-bromophenyl)benzohydrazide (**B**$_5$). Yield: 67%; light yellow crystal; m.p. 155.6–157.4 ^{o}C; ^{1}H NMR (400 MHz, DMSO-d_6): *δ* 10.43 (d, *J* = 2.6 Hz, 1H), 8.23 (d, *J* = 2.5 Hz, 1H), 7.95 – 7.88 (m, 2H), 7.60 (t, *J* = 7.3 Hz, 1H), 7.52 (t, *J* = 7.5 Hz, 2H), 7.11 (t, *J* = 8.0 Hz, 1H), 6.95 – 6.83 (m, 2H), 6.78 (dd, *J* = 8.1, 2.1 Hz, 1H); ^{13}C NMR (100 MHz, DMSO-d_6): *δ* 166.8, 151.7, 133.1, 132.2, 131.2, 129.0, 127.8, 122.5, 121.4, 114.8, 111.7. This is a known compound and the spectral data are identical to those reported in the literature [6].

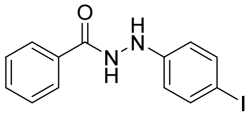

N'-(4-iodophenyl)benzohydrazide (**B**$_6$). Yield: 73%; Brown crystal; m.p. 165.8–166.2 ^{o}C; ^{1}H NMR (400 MHz, DMSO-d_6): *δ* 10.43 (d, *J* = 2.5 Hz, 1H), 8.16 (s, 1H), 7.92 (d, *J* = 7.4 Hz, 2H), 7.58 (t, *J* = 7.2 Hz, 1H), 7.51 (t, *J* = 7.5 Hz, 3H), 7.46 (d, *J* = 8.4 Hz, 2H), 6.64 (d, *J* = 8.5 Hz, 2H); ^{13}C NMR (100 MHz, DMSO-d_6): *δ* 166.7, 149.9, 137.6, 133.29 132.2, 128.9, 127.8, 115.3, 80.3. HRMS (ESI, positive) *m/z*: calcd for $C_{13}H_{12}IN_2O^+$ [M+H]$^+$, 338.9994, found, 338.9988. This is a known compound and the spectral data are identical to those reported in the literature [5].

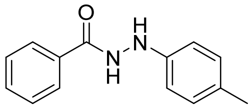

N'-(p-tolyl)benzohydrazide (**B**$_7$). Yield: 66%; white solid; m.p. 137.5–138.2 ^{o}C; ^{1}H NMR (400

MHz, DMSO-d_6): δ 10.35 (d, J = 3.3 Hz, 1H), 7.95 – 7.86 (m, 2H), 7.74 (d, J = 3.2 Hz, 1H), 7.61 – 7.53 (m, 1H), 7.50 (t, J = 7.5 Hz, 2H), 6.97 (d, J = 8.1 Hz, 2H), 6.71 (d, J = 8.1 Hz, 2H), 2.18 (s, 3H); ^{13}C NMR (100 MHz, DMSO-d_6): δ 166.7, 147.7, 133.5, 132.0, 129.6, 128.9, 127.7, 113.0, 20.6. It is a known compound and the spectral data are identical to those reported in the literature [1].

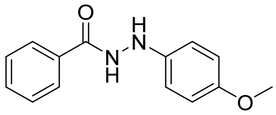

N'-(4-methoxyphenyl)benzohydrazide (**B₈**). Yield: 67%; white solid; m.p. 132.6–133.4 °C; ^{1}H NMR (400 MHz, DMSO-d_6): δ 10.37 (d, J = 3.6 Hz, 1H), 7.92 (d, J = 6.7 Hz, 1H), 7.61 (d, J = 3.6 Hz, 1H), 7.57 (d, J = 7.4 Hz, 1H), 7.50 (t, J = 7.5 Hz, 2H), 6.78 (s, 4H), 3.67 (s, 3H); ^{13}C NMR (100 MHz, DMSO-d_6): δ 166.7, 153.2, 143.8, 133.5, 132.0, 128.9, 127.7, 114.7, 114.3, 55.7. This is a known compound and the spectral data are identical to those reported in the literature [1].

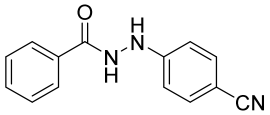

N'-(4-cyanophenyl)benzohydrazide (**B_9**). Yield: 62%; white solid; m.p. 190.2–190.7 °C; ^{1}H NMR (400 MHz, DMSO-d_6): δ 10.54 (d, J = 1.8 Hz, 1H), 8.84 (d, J = 1.8 Hz, 1H), 7.93 (d, J = 7.6 Hz, 2H), 7.57 (tt, J = 15.1, 7.4 Hz, 6H), 6.84 (d, J = 8.5 Hz, 2H); ^{13}C NMR (100 MHz, DMSO-d_6): δ 166.8, 153.5, 133.9, 132.9, 132.4, 129.0, 127.8, 120.5, 112.2, 99.4. This is a known compound and the spectral data are identical to those reported in the literature [12].

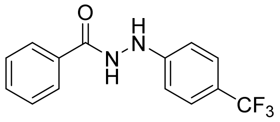

N'-(4-(trifluoromethyl)phenyl)benzohydrazide (**B_{10}**). Yield: 56%; white needle like solid; m.p. 153.4–154.6 °C; ^{1}H NMR (400 MHz, DMSO-d_6): δ 10.53 ((d, J = 2.0 Hz, 1H), 8.61 (d, J = 2.2 Hz, 1H), 7.94 (d, J = 7.5 Hz, 2H), 7.60 (t, J = 7.3 Hz, 1H), 7.52 (dd, J = 16.9, 8.2 Hz, 4H), 6.89 (d, J = 8.4 Hz, 2H); ^{13}C NMR (100 MHz, DMSO-d_6): δ 166.8, 153.1, 133.1, 132.3, 129.0, 127.8, 126.7 (q, J = 3.8 Hz), 125.5(q, J = 271.69 Hz), 118.7 (q, J = 31.7 Hz), 112.0. This is a known compound and the spectral data are identical to those reported in the literature [9].

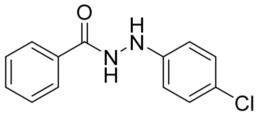

N'-(4-chlorophenyl)benzohydrazide (**B_{11}**). Yield: 74%; Brown crystal; m.p. 140.3–141.3 °C; ^{1}H NMR (400 MHz, DMSO-d_6): δ 10.41 (d, J = 2.7 Hz, 1H), 8.12 (d, J = 2.7 Hz, 1H), 7.92 (d, J = 7.0 Hz, 2H), 7.59 (t, J = 7.3 Hz, 1H), 7.51 (t, J = 7.5 Hz, 2H), 7.22 – 7.16 (m, 2H), 6.82 – 6.75 (m, 2H); ^{13}C NMR (100 MHz, DMSO-d_6): δ 166.7, 148.9, 133.2, 132.2, 129.0, 128.9, 127.7, 122.3, 114.2. This

is a known compound and the spectral data are identical to those reported in the literature [1].

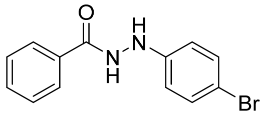

N'-(4-bromophenyl)benzohydrazide (**B12**). Yield: 68%; Brown crystal; m.p. 138.2–139.6 °C; ^{1}H NMR (400 MHz, DMSO-*d6*): *δ* 10.41 (d, *J* = 2.7 Hz, 1H), 8.14 (d, *J* = 2.6 Hz, 1H), 7.98 – 7.83 (m, 2H), 7.61 – 7.55 (m, 1H), 7.51 (t, *J* = 7.5 Hz, 2H), 7.34 – 7.27 (m, 2H), 6.77 – 6.70 (m, 2H); ^{13}C NMR (100 MHz, DMSO-*d6*): *δ* 166.7, 149.3, 133.2, 132.2, 131.8, 128.9, 127.7, 114.7, 109.8. This is a known compound and the spectral data are identical to those reported in the literature [1].

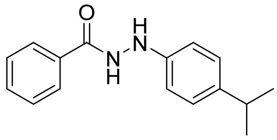

N'-(4-isopropylphenyl)benzohydrazide (**B13**). Yield: 62%; white solid; m.p. 142.8–143.5 °C; ^{1}H NMR (400 MHz, DMSO-*d6*): *δ* 10.35 (s, 1H), 7.92 (s, 1H), 7.90 (d, *J* = 1.6 Hz, 1H), 7.74 (s, 1H), 7.60 – 7.55 (m, 1H), 7.50 (t, *J* = 7.4 Hz, 2H), 7.06 – 6.99 (m, 2H), 6.76 – 6.69 (m, 2H), 2.76 (p, *J* = 6.9 Hz, 1H), 1.16 (s, 3H), 1.14 (s, 3H); ^{13}C NMR (100 MHz, DMSO-*d6*): *δ* 166.7, 148.0, 139.1, 133.5, 132.0, 128.9, 127.7, 126.9, 112.9, 33.1, 24.7. HRMS (ESI, positive) *m*/*z*: calcd for $C_{16}H_{18}N_2O^+$ [M+H]$^+$, 255.1497, found, 255.1493. This is a known compound and the spectral data are identical to those reported in the literature [5]

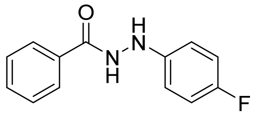

N'-(4-fluorophenyl)benzohydrazide (**B14**). Yield: 74%; white solid; m.p. 139.2–139.6 °C; ^{1}H NMR (400 MHz, DMSO-*d6*): *δ* 10.40 (d, *J* = 3.2 Hz, 1H), 7.91 (dd, *J* = 10.7, 5.4 Hz, 4H), 7.62 – 7.54 (m, 1H), 7.50 (t, *J* = 7.4 Hz, 2H), 7.00 (t, *J* = 8.9 Hz, 2H), 6.81 (dd, *J* = 8.9, 4.6 Hz, 2H); ^{13}C NMR (100 MHz, DMSO-*d6*): *δ* 166.8, 156.4 (d, *J* = 233.4 Hz), 146.5, 133.4, 132.1, 128.97, 127.7, 115.6 (d, *J* = 22.4 Hz), 114.0 (d, *J* = 7.5 Hz). This is a known compound and the spectral data are identical to those reported in the literature [1].

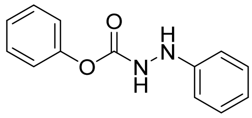

phenyl 2-phenylhydrazine-1-carboxylate (**C1**). Yield: 62%; white solid; m.p. 112.5–114.3 °C，^{1}H NMR (400 MHz, DMSO-*d6*) *δ* 9.59 (d, *J* = 2.8 Hz, 1H), 7.66 (d, *J* = 2.9 Hz, 1H), 7.12 (dd, *J* = 8.7, 7.1 Hz, 2H), 6.71 – 6.66 (m, 3H), 2.15 (t, *J* = 7.4 Hz, 2H), 1.55 (p, *J* = 7.3 Hz, 2H), 1.28 (tdt, *J* =

10.3, 7.1, 4.2 Hz, 4H), 0.88 (t, *J* = 6.9 Hz, 3H). ^{13}C NMR (101 MHz, DMSO-d_6) δ 155.69, 151.29, 149.48, 129.90, 129.36, 125.73, 122.13, 119.17, 112.39, 112.20. This is a known compound and the spectral data are identical to those reported in the literature [13].

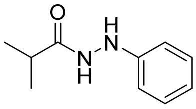

N'-phenylisobutyrohydrazide (**C2**). Yield: 90%; white crystal; m.p. 134.9-135.7 °C, ^{1}H NMR (400 MHz, DMSO-d_6): *δ* 9.58 (d, *J* = 2.4 Hz, 1H), 7.64 (d, *J* = 2.7 Hz, 1H), 7.12 (t, *J* = 7.8 Hz, 2H), 6.70 – 6.65 (m, 3H), 2.49 – 2.43 (m, 1H), 1.06 (d, *J* = 6.9 Hz, 6H). ^{13}C NMR (101 MHz, DMSO-d_6) δ 177.16, 149.68, 129.22, 119.26, 112.64, 32.76, 19.74. This is a known compound and the spectral data are identical to those reported in the literature [14].

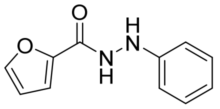

N'-phenylfuran-2-carbohydrazide (**C3**). Yield: 62%; white needle like solid; m.p. 141.2–142.3 °C; ^{1}H NMR (400 MHz, DMSO-d_6): *δ* 10.28 (s, 1H), 7.91 (d, *J* = 4.8 Hz, 3H), 7.25 (d, *J* = 3.5 Hz, 1H), 7.15 (t, *J* = 7.7 Hz, 2H), 6.72 (dd, *J* = 14.1, 7.5 Hz, 3H), 6.67 (dd, *J* = 3.4, 1.7 Hz, 1H); ^{13}C NMR (100 MHz, DMSO-d_6): *δ* 158.4, 149.8, 147.0, 146.0, 129.2, 119.0, 114.5, 112.6, 112.2. This is a known compound and the spectral data are identical to those reported in the literature [15].

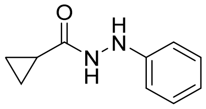

N'-phenylcyclopropanecarbohydrazide (**C4**). Yield: 85%; white crystal; m.p. 181.9–183.6 °C; ^{1}H NMR (400 MHz, DMSO-d_6): *δ* 9.83 (s, 1H), 7.69 (s, 1H), 7.13 (t, *J* = 7.7 Hz, 2H), 6.71 – 6.63 (m, 3H), 1.64 (ddd, *J* = 10.5, 7.9, 4.7 Hz, 1H), 0.78 – 0.62 (m, 4H); ^{13}C NMR (100 MHz, DMSO-d_6): *δ* 173.1, 149.9, 129.1, 118.8, 112.5, 12.4, 6.7. This is a known compound and the spectral data are identical to those reported in the literature [16].

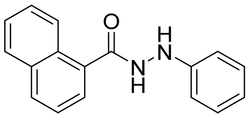

N'-phenyl-1-naphthohydrazide (**C5**). Yield: 61%; White flocculent solid; m.p. 184.1–184.7 °C; ^{1}H NMR (400 MHz, DMSO-d_6): *δ* 10.36 (s, 1H), 8.24 – 8.17 (m, 1H), 8.08 (d, *J* = 8.3 Hz, 1H), 8.05 – 7.98 (m, 2H), 7.78 (d, *J* = 6.9 Hz, 1H), 7.65 – 7.50 (m, 3H), 7.21 (t, *J* = 7.7 Hz, 2H), 6.89 (d, *J* = 7.9 Hz, 2H), 6.76 (t, *J* = 7.3 Hz, 1H); ^{13}C NMR (100 MHz, DMSO-d_6): *δ* 168.9, 149.9, 133.6, 133.1, 130.8, 130.4, 129.3, 128.8, 127.4, 126.8, 126.1, 125.5, 119.1, 112.7. This is a known compound and the spectral data are identical to those reported in the literature [9].

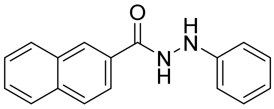

N'-phenyl-2-naphthohydrazide (**C_6**). Yield: 68%; white needle like solid; m.p. 172.4–173.6 °C; ^{1}H NMR (400 MHz, DMSO-d_6): δ 10.56 (s, 1H), 8.59 (s, 1H), 8.04 (dt, J = 15.1, 8.4 Hz, 5H), 7.63 (p, J = 6.6 Hz, 2H), 7.19 (t, J = 7.6 Hz, 2H), 6.87 (d, J = 8.0 Hz, 2H), 6.75 (t, J = 7.3 Hz, 1H); ^{13}C NMR (100 MHz, DMSO-d_6): δ 166.9, 150.0, 134.8, 132.6, 130.8, 129.4, 129.2, 128.5, 128.2, 128.1, 127.3, 124.5, 119.1, 112.8. HRMS (ESI, positive) *m/z*: calcd for $C_{17}H_{15}N_2O^+$ [M+H]$^+$, 263.1184, found, 263.1187. This is a known compound and the spectral data are identical to those reported in the literature [5].

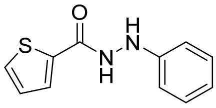

N'-phenylthiophene-2-carbohydrazide (**C_7**). Yield: 65%; white needle like solid; m.p. 138.5–139.6 °C; ^{1}H NMR (400 MHz, DMSO-d_6): δ 10.41 (s, 1H), 7.98 (s, 1H), 7.92 (d, J = 3.8 Hz, 1H), 7.83 (d, J = 4.9 Hz, 1H), 7.20 (t, J = 4.4 Hz, 1H), 7.16 (t, J = 7.7 Hz, 2H), 6.77 (d, J = 8.0 Hz, 2H), 6.72 (t, J = 7.3 Hz, 1H); ^{13}C NMR (100 MHz, DMSO-d_6): δ 161.8, 149.8, 138.3, 131.8, 129.28, 128.9, 128.6, 119.1, 112.7. HRMS (ESI, positive) *m/z*: calcd for $C_{11}H_{11}N_2OS^+$ [M+H]$^+$, 219.0592, found, 219.0590. This is a known compound and the spectral data are identical to those reported in the literature [5].

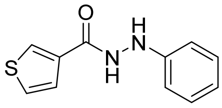

N'-phenylthiophene-3-carbohydrazide (**C_8**). Yield: 61%; white needle like solid; m.p. 182.4–183.6 °C; ^{1}H NMR (400 MHz, DMSO-d_6): δ 10.22 (s, 1H), 8.26 (dd, J = 3.0, 1.3 Hz, 1H), 7.91 (s, 1H), 7.63 (dd, J = 5.1, 2.9 Hz, 1H), 7.59 (dd, J = 5.1, 1.3 Hz, 1H), 7.15 (dd, J = 8.6, 7.2 Hz, 2H), 6.77 (d, J = 7.6 Hz, 2H), 6.71 (td, J = 7.3, 1.3 Hz, 1H); ^{13}C NMR (100 MHz, DMSO-d_6): δ 162.5, 149.9, 136.3, 129.8, 129.2, 127.5, 127.1, 119.0, 112.7. HRMS (ESI, positive) *m/z*: calcd for $C_{11}H_{11}N_2OS^+$ [M+H]$^+$, 219.0592, found, 219.0596. This is a known compound and the spectral data are identical to those reported in the literature [5].

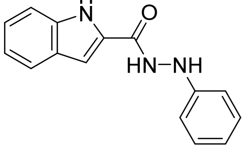

N'-phenyl-1H-indole-2-carbohydrazide (**C_9**). Yield: 55%; brown solid; m.p. 231.6–232.3 °C; ^{1}H NMR (400 MHz, DMSO-d_6): δ 11.68 (s, 1H), 10.38 (d, J = 2.7 Hz, 1H), 7.99 (d, J = 2.6 Hz, 1H),

7.65 (d, *J* = 8.0 Hz, 1H), 7.45 (d, *J* = 8.2 Hz, 1H), 7.29 (s, 1H), 7.18 (dt, *J* = 15.9, 7.5 Hz, 3H), 7.06 (t, J = 7.5 Hz, 1H), 6.80 (d, J = 8.0 Hz, 2H), 6.72 (t, J = 7.3 Hz, 1H); ^{13}C NMR (100 MHz, DMSO-d6): *δ* 161.8, 149.9, 137.0, 130.3, 129.2, 124.0, 122.0, 120.3, 119.0, 112.8, 112.6, 103.3. HRMS (ESI, positive) *m/z*: calcd for $C_{15}H_{14}N_3O^+$ [M+H]$^+$, 252.1137, found, 252.1134. This is a known compound and the spectral data are identical to those reported in the literature [5].

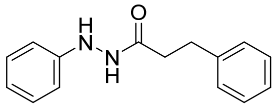

N',3-diphenylpropanehydrazide (**C**$_{10}$). Yield: 73%; light yellow solid; m.p. 91.6–92.7 °C; ^{1}H NMR (400 MHz, DMSO-d_6): *δ* 9.58 (d, *J* = 2.3 Hz, 1H), 7.65 (d, *J* = 2.6 Hz, 1H), 7.34 – 7.26 (m, 2H), 7.26 – 7.18 (m, 3H), 7.06 (dd, *J* = 8.5, 7.2 Hz, 2H), 6.66 (t, *J* = 7.3 Hz, 1H), 6.57 – 6.50 (m, 2H), 2.87 (t, *J* = 7.5 Hz, 2H), 2.49 (d, *J* = 7.5 Hz, 2H); ^{13}C NMR (100 MHz, DMSO-d_6): *δ* 171.6, 149.7, 141.4, 129.0, 128.8, 128.7, 126.4, 118.7, 112.5, 35.3, 31.3. This is a known compound and the spectral data are identical to those reported in the literature [17].

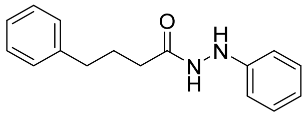

N',4-diphenylbutanehydrazide (**C**$_{11}$). Yield: 69%; yellow crystal; m.p. 99.8–100.8 °C; ^{1}H NMR (400 MHz, DMSO-d_6): *δ* 9.62 (d, *J* = 3.0 Hz, 1H), 7.68 (d, *J* = 2.9 Hz, 1H), 7.34 – 7.23 (m, 2H), 7.23 – 7.17 (m, 3H), 7.13 (dd, *J* = 8.5, 7.1 Hz, 2H), 6.68 (t, *J* = 8.0 Hz, 3H), 2.64 – 2.53 (m, 2H), 2.19 (t, *J* = 7.4 Hz, 2H), 1.85 (p, *J* = .07.6 Hz, 2H). ^{13}C NMR (101 MHz, DMSO-d_6) δ 172.25, 149.97, 142.13, 129.14, 128.80, 126.30, 118.83, 112.55, 35.14, 33.33, 27.48. This is a known compound and the spectral data are identical to those reported in the literature [18].

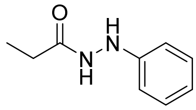

N'-phenylacetohydrazide (**C**$_{12}$). Yield: 88%; white crystal; m.p. 122.7–123.4 °C; ^{1}H NMR (400 MHz, DMSO-d_6): *δ* 9.59 (s, 1H), 7.64 (s, 1H), 7.12 (dd, *J* = 8.7, 7.0 Hz, 2H), 6.72 – 6.65 (m, 3H), 1.89 (s, 3H). ^{13}C NMR (101 MHz, DMSO-d_6) δ 173.41, 149.92, 129.14, 118.88, 112.57, 27.12, 10.32. This is a known compound and the spectral data are identical to those reported in the literature [19].

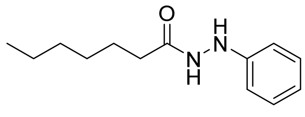

N'-phenylheptanehydrazide (**C**$_{13}$). Yield: 84%; white crystal; m.p. 97.3–98.2 °C; ^{1}H NMR (400 MHz, DMSO-d_6): *δ* 9.58 (s, 1H), 7.66 (s, 1H), 7.11 (t, *J* = 7.9 Hz, 2H), 6.71 – 6.65 (m, 3H), 2.15 (t, *J*

= 7.3 Hz, 2H), 1.54 (t, *J* = 7.1 Hz, 2H), 1.28 (s, 6H), 0.91 – 0.84 (m, 3H). ^{13}C NMR (101 MHz, DMSO-d_6) δ 172.49, 150.00, 129.09, 118.80, 112.54, 33.75, 31.34, 25.31, 24.28, 22.33, 14.34. This is a known compound and the spectral data are identical to those reported in the literature [20].

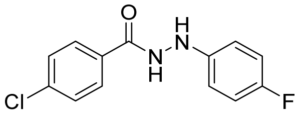

4-chloro-N'-(4-fluorophenyl)benzohydrazide (**D$_1$**). Yield: 62%; white solid; m.p. 158.4–159.1 °C; ^{1}H NMR (400 MHz, DMSO-d_6): *δ* 10.49 (d, *J* = 3.1 Hz, 1H), 7.98 – 7.91 (m, 3H), 7.62 – 7.56 (m, 2H), 7.01 (t, *J* = 8.9 Hz, 2H), 6.86 – 6.77 (m, 2H); ^{13}C NMR (100 MHz, DMSO-d_6): *δ* 165.8, 156.4 (d, *J* = 233.8 Hz), 146.3, 137.0, 132.1, 129.7, 129.0, 115.6 (d, *J* = 22.3 Hz), 114.0 (d, *J* = 7.5 Hz). HRMS (ESI, positive) *m*/*z*: calcd for $C_{13}H_{11}ClFN_2OS^+$ [M+H]$^+$, 265.0538, found, 265.0535. This is a known compound and the spectral data are identical to those reported in the literature [5].

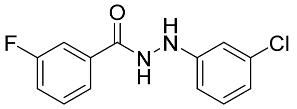

N'-(3-chlorophenyl)-3-fluorobenzohydrazide (**D$_2$**). Yield: 70%; white needle shaped crystal; m.p. 199.1–212.2 °C; ^{1}H NMR (400 MHz, DMSO-d_6) *δ* 10.54 (d, *J* = 2.6 Hz, 1H), 8.31 (d, *J* = 2.6 Hz, 1H), 7.83 – 7.69 (m, 2H), 7.59 (td, *J* = 8.0, 5.8 Hz, 1H), 7.46 (td, *J* = 8.6, 2.7 Hz, 1H), 7.18 (t, *J* = 7.9 Hz, 1H), 6.82 – 6.69 (m, 3H). ^{13}C NMR (100 MHz, DMSO-*d6*) *δ* 165.5 (d, *J* = 2.5 Hz), 163.6, 161.2, 151.4, 135.4 (d, *J* = 6.9 Hz), 133.9, 131.2 (d, *J* = 8.1 Hz), 130.9, 124.0 (d, *J* = 2.9 Hz), 119.2 (d, *J* = 21.1 Hz), 118.6, 114.6 (d, *J* = 22.9 Hz), 112.0, 111.4. HRMS (ESI, positive) *m*/*z*: calcd for $C_{13}H_{11}ClFN_2OS^+$ [M+H]$^+$, 265.0538, found, 265.0533 This is a known compound and the spectral data are identical to those reported in the literature [21].

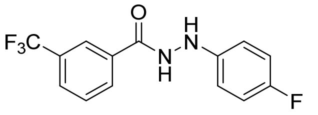

N'-(4-fluorophenyl)-3-(trifluoromethyl)benzohydrazide (**D$_3$**). Yield: 65%; white flaky solid; m.p. 180.4–186.5 °C; ^{1}H NMR (400 MHz, DMSO-d_6) *δ* 10.68 (d, *J* = 3.1 Hz, 1H), 8.26 (d, *J* = 2.3 Hz, 1H), 8.25 – 8.21 (m, 1H), 8.04 – 7.91 (m, 2H), 7.78 (t, *J* = 7.8 Hz, 1H), 7.08 – 6.95 (m, 2H), 6.88 – 6.77 (m, 2H). ^{13}C NMR (100 MHz, DMSO-*d6*) *δ* 165.3, 157.6, 155.3, 146.2, 146.2, 134.2, 131.9, 130.3, 129.7 (q, *J* = 32.0 Hz), 128.7 (d, *J* = 3.8 Hz), 125.8 – 122.8 (m), 115.7 (d, *J* = 22. Hz), 114.0 (d, *J* = 7.6 Hz). This is a known compound and the spectral data are identical to those reported in the literature [22].

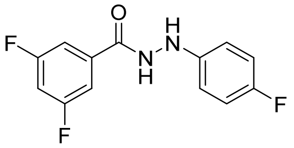

3,5-difluoro-N'-(4-fluorophenyl)benzohydrazide (**D4**). Yield: 71%; White needle shaped crystal; m.p. 189.6–197.9 °C; ^{1}H NMR (400 MHz, DMSO-*d*6) δ 10.59 (d, *J* = 3.1 Hz, 1H), 8.01 (d, *J* = 3.0 Hz, 1H), 7.69 – 7.59 (m, 2H), 7.53 (tt, *J* = 9.1, 2.4 Hz, 1H), 7.06 – 6.95 (m, 2H), 6.87 – 6.75 (m, 2H). ^{13}C NMR (100 MHz, DMSO-*d6*) δ 164.3 (t, *J* = 2.8 Hz), 163.9 (d, *J* = 12.7 Hz), 161.5 (d, *J* = 12.8 Hz), 156.4 (d, *J* = 233.6 Hz), 146.1 (d, *J* = 1.8 Hz), 136.7 (t, *J* = 8.6 Hz), 115.7 (d, *J* = 22.3 Hz), 114.1 (d, *J* = 7.7 Hz), 111.8 – 110.8 (m), 107.7 (t, *J* = 25.9 Hz). HRMS (ESI, positive) *m/z*: calcd for $C_{13}H_{10}F_3N_2OS^+$ [M+H]$^+$, 267.0740, found, 267.0735.

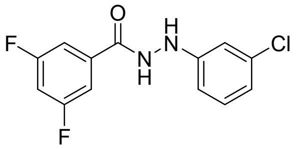

N'-(3-chlorophenyl)-3,5-difluorobenzohydrazide（**D5**）: Yield, 65%; white solid; m.p. 205.5–209.5 °C; ^{1}H NMR (400 MHz, DMSO-*d*6) δ 10.61 (d, *J* = 2.4 Hz, 1H), 8.33 (d, *J* = 2.4 Hz, 1H), 7.64 (dt, *J* = 6.6, 2.2 Hz, 2H), 7.55 (tt, *J* = 9.1, 2.4 Hz, 1H), 7.18 (t, *J* = 8.0 Hz, 1H), 6.82 – 6.71 (m, 3H). ^{13}C NMR (101 MHz, DMSO-*d6*) δ 164.3 (t, *J* = 2.8 Hz), 164.0 (d, *J* = 12.7 Hz), 16.54 (d, *J* = 12.5 Hz), 151.1, 136.5 (t, *J* = 8.6 Hz), 133.9, 130.9, 118.7, 112.1, 111.5 – 111.1 (m), 107.8 (t, *J* = 25.9 Hz). HRMS (ESI, positive) *m/z*: calcd for $C_{13}H_{10}ClF_2N_2OS^+$ [M+H]$^+$, 283.0444, found, 283.4489 [M+H]$^+$.

2. The spectra of all synthesized compounds

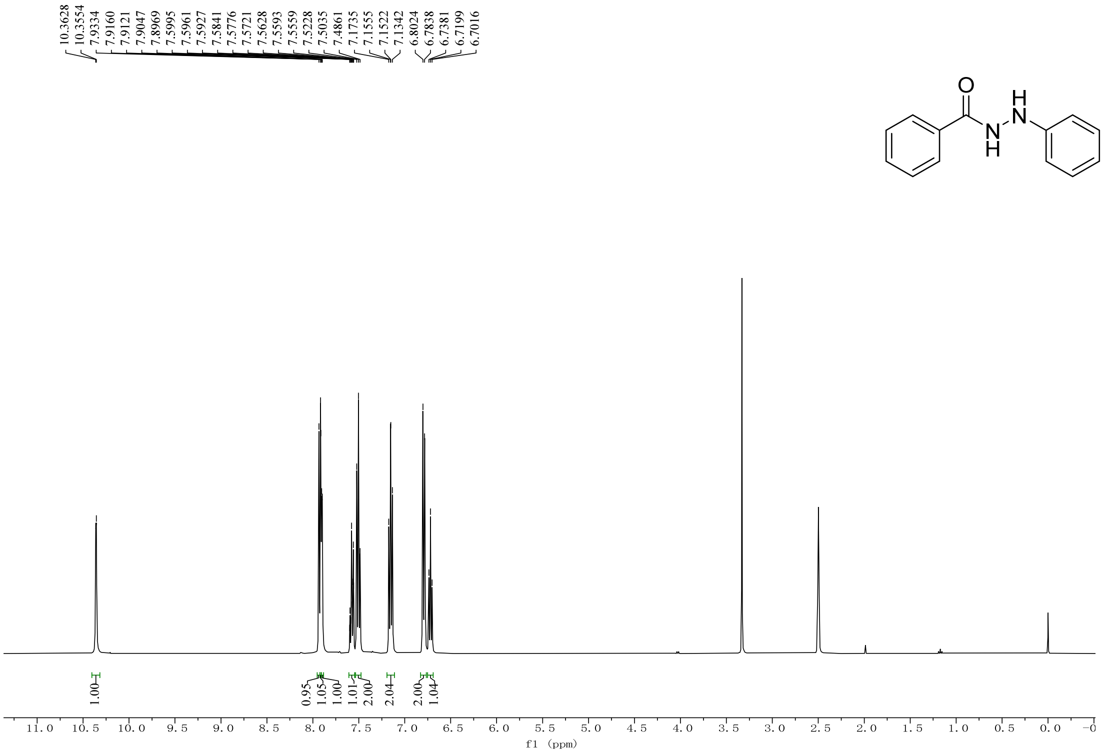

The ^{1}H NMR spectrum of **A**1.

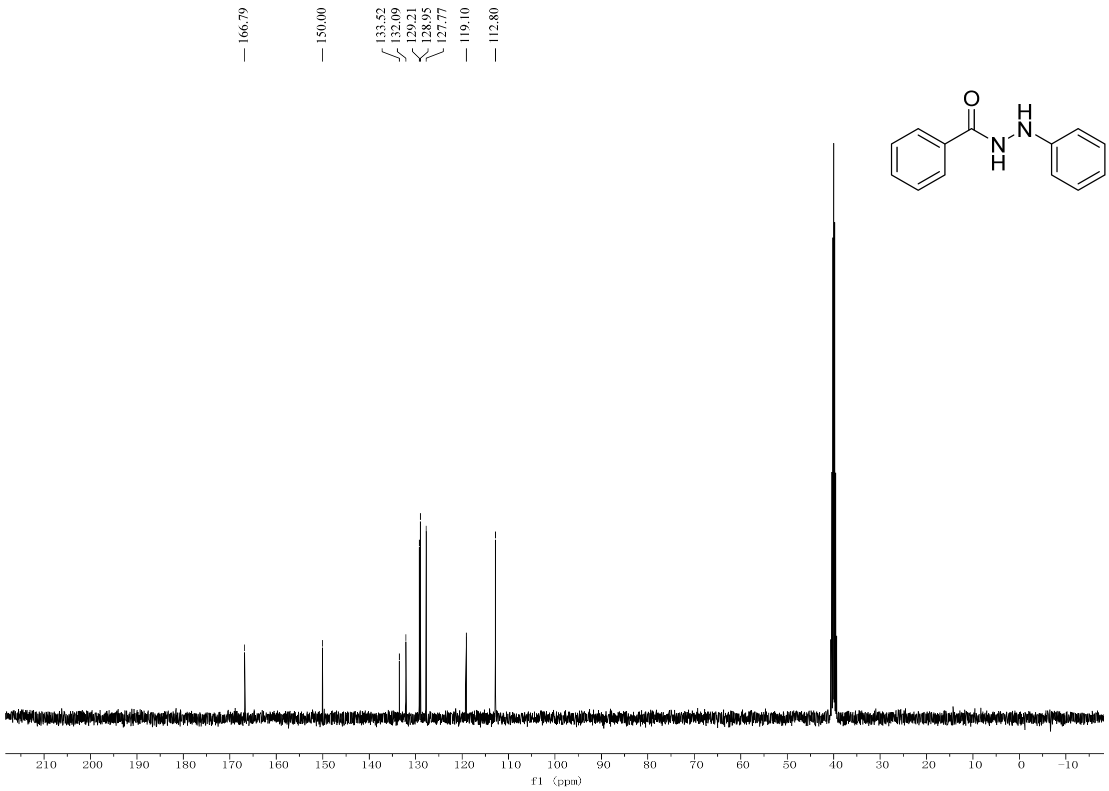

The ^{13}C NMR spectrum of **A1**.

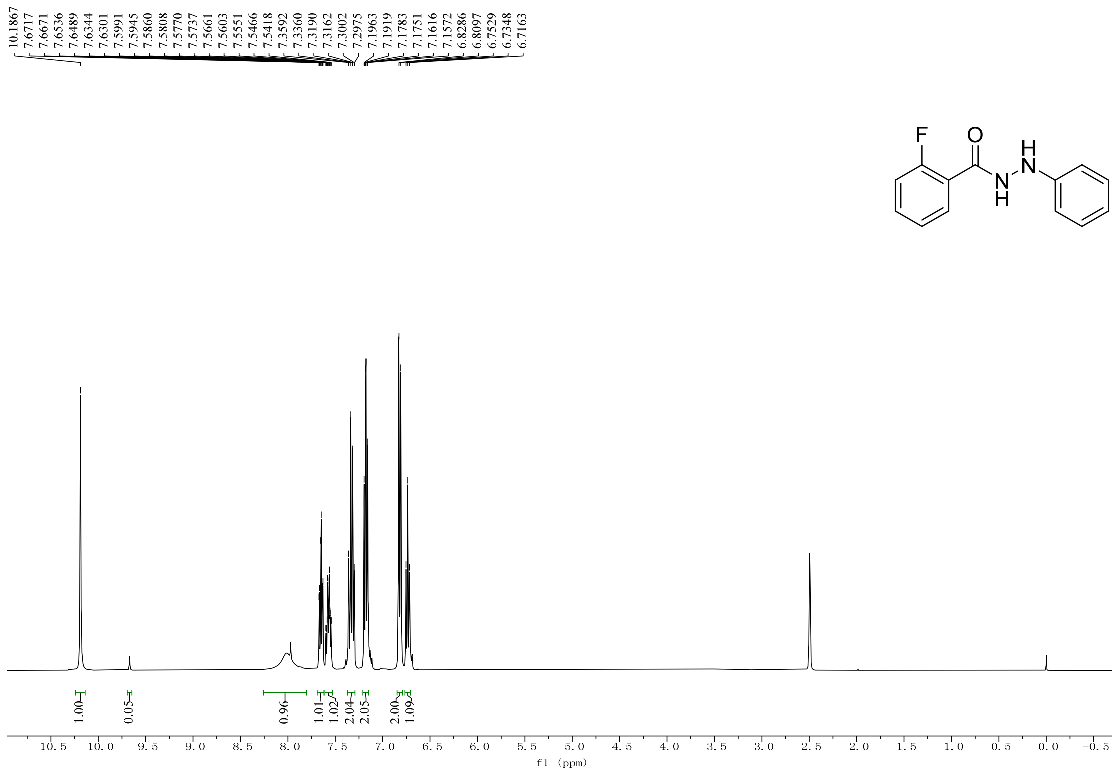

The ^{1}H NMR spectrum of **A2**.

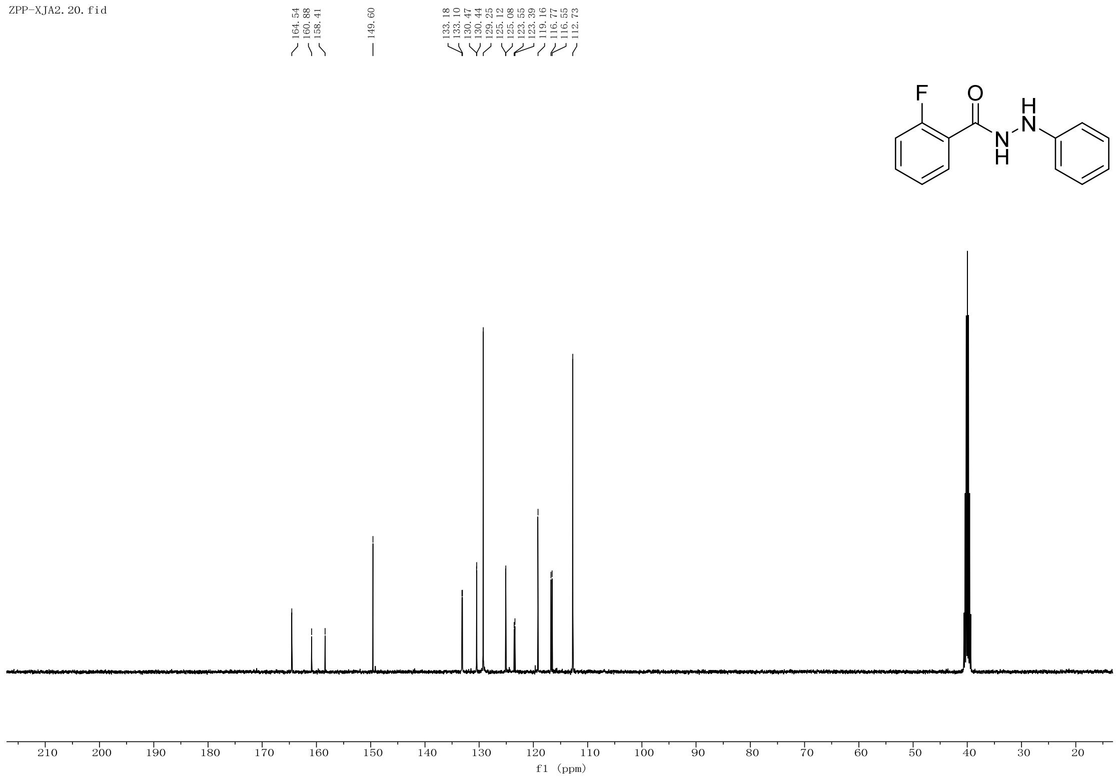

The ^{13}C NMR spectrum of **A2**.

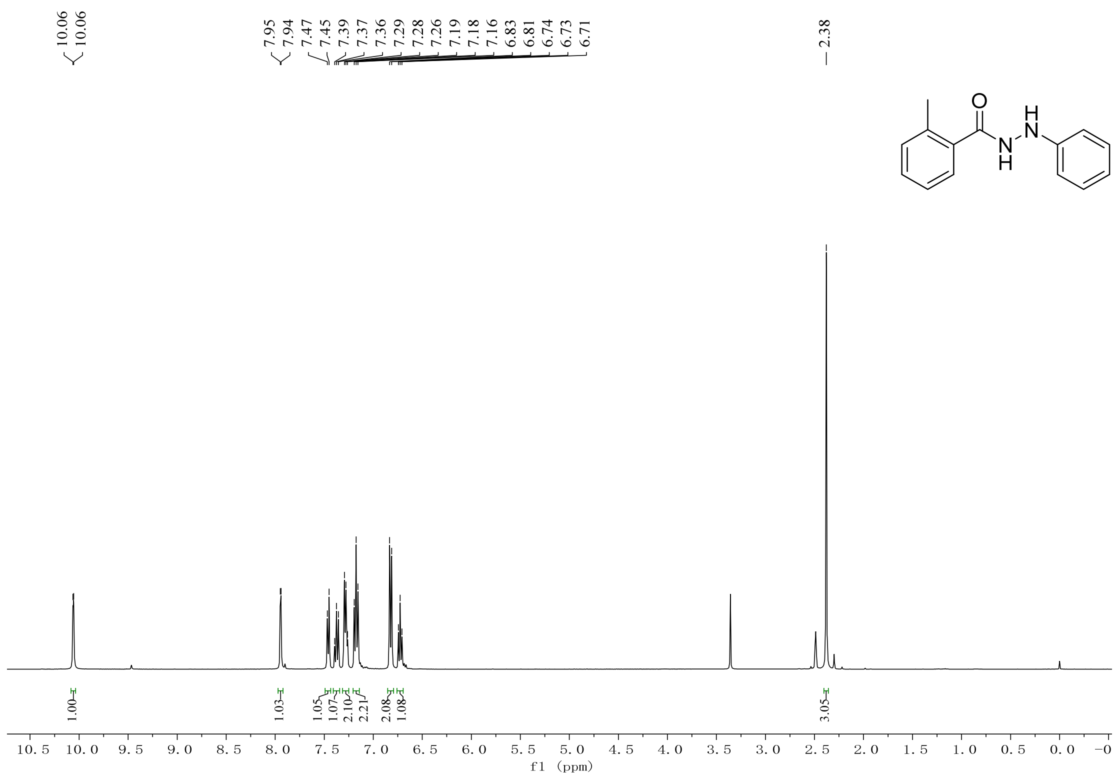

The ^{1}H NMR spectrum of **A**3.

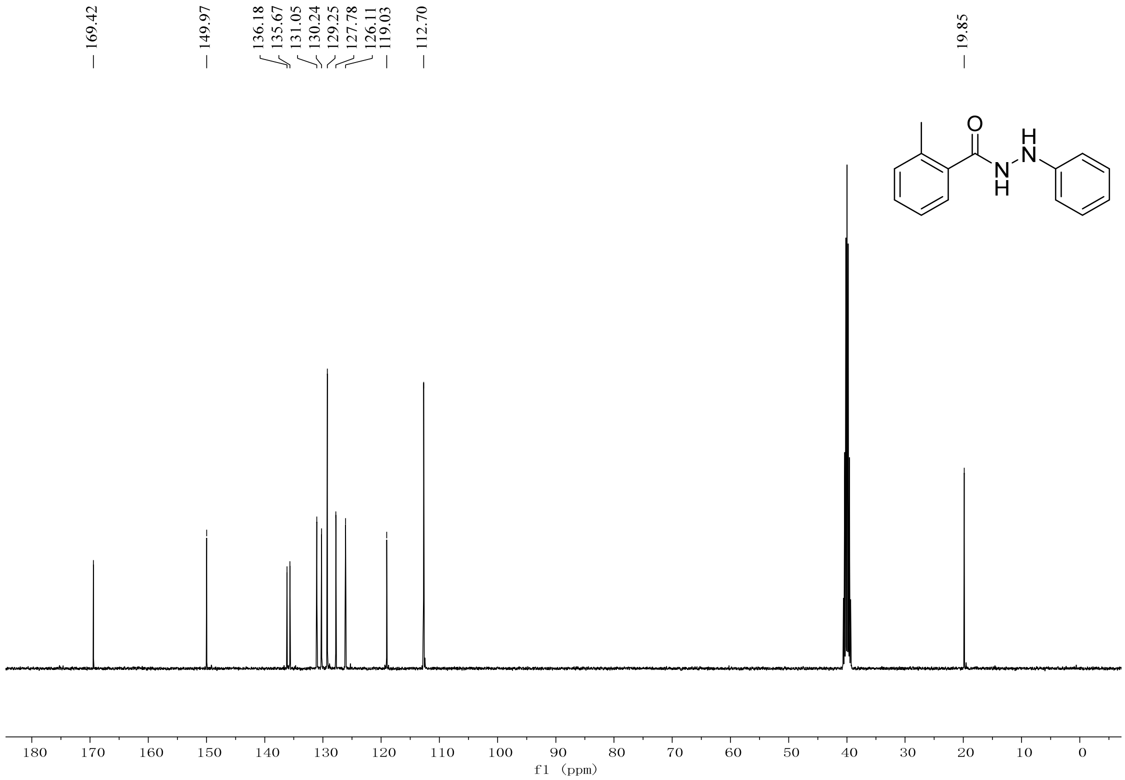

The ^{13}C NMR spectrum of **A**3.

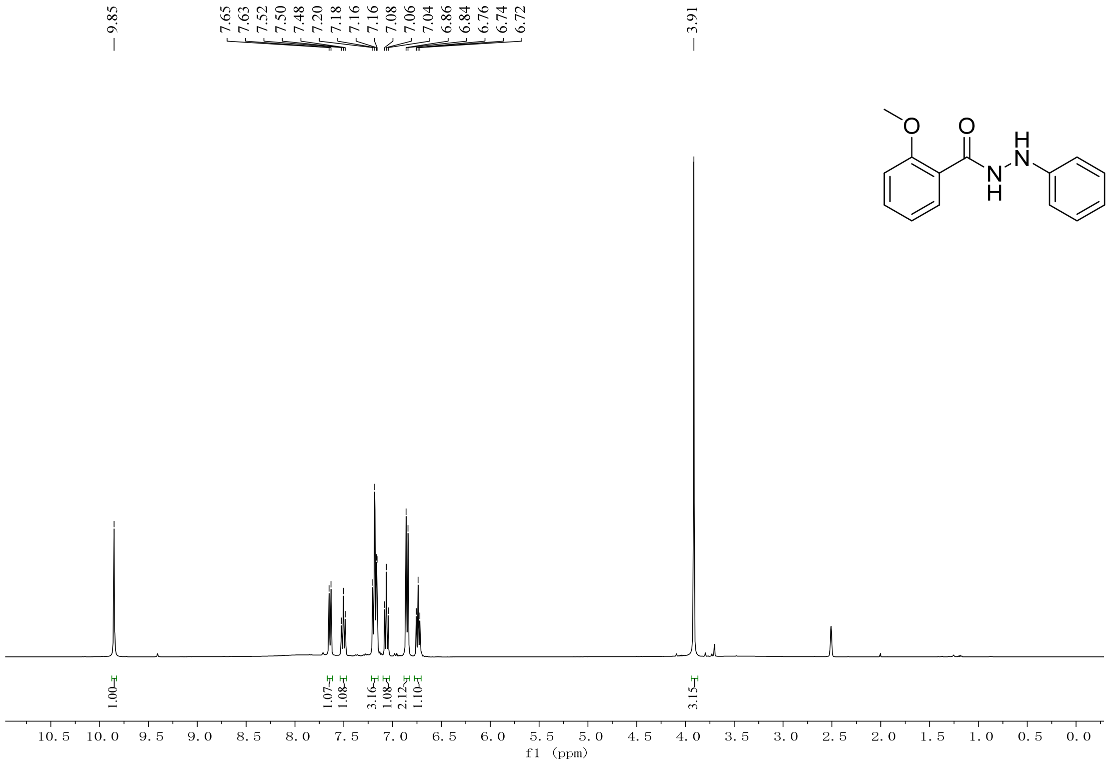

The ^{1}H NMR spectrum of **A4**.

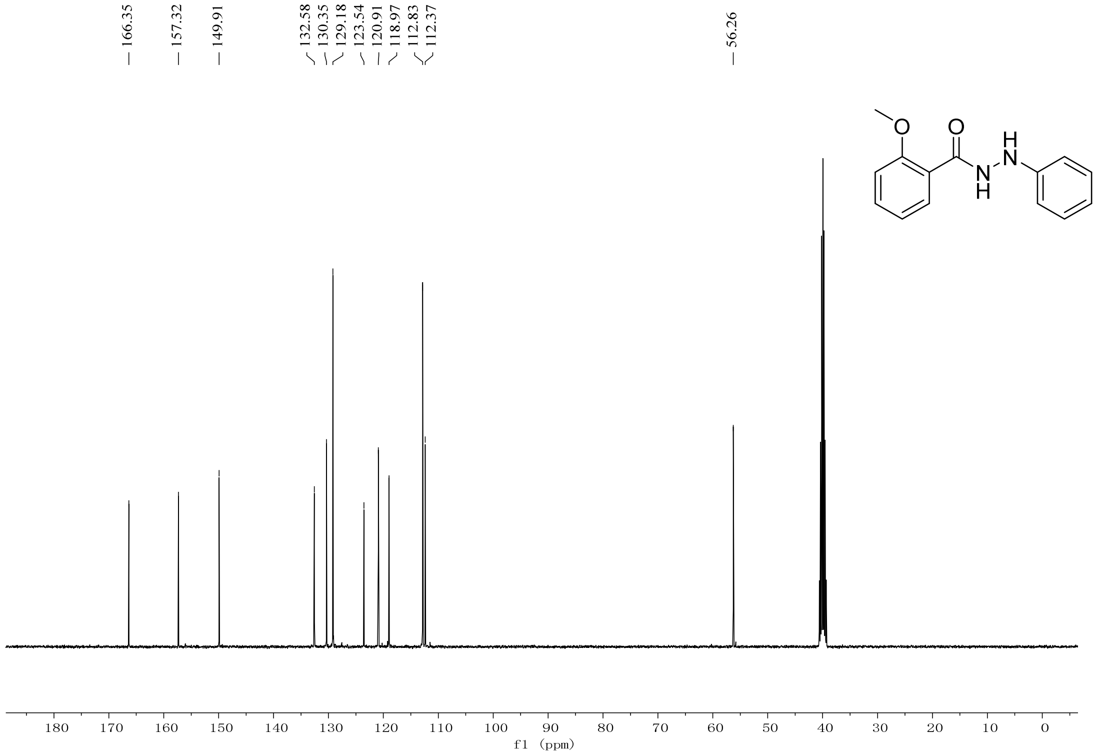

The ^{13}C NMR spectrum of **A4**.

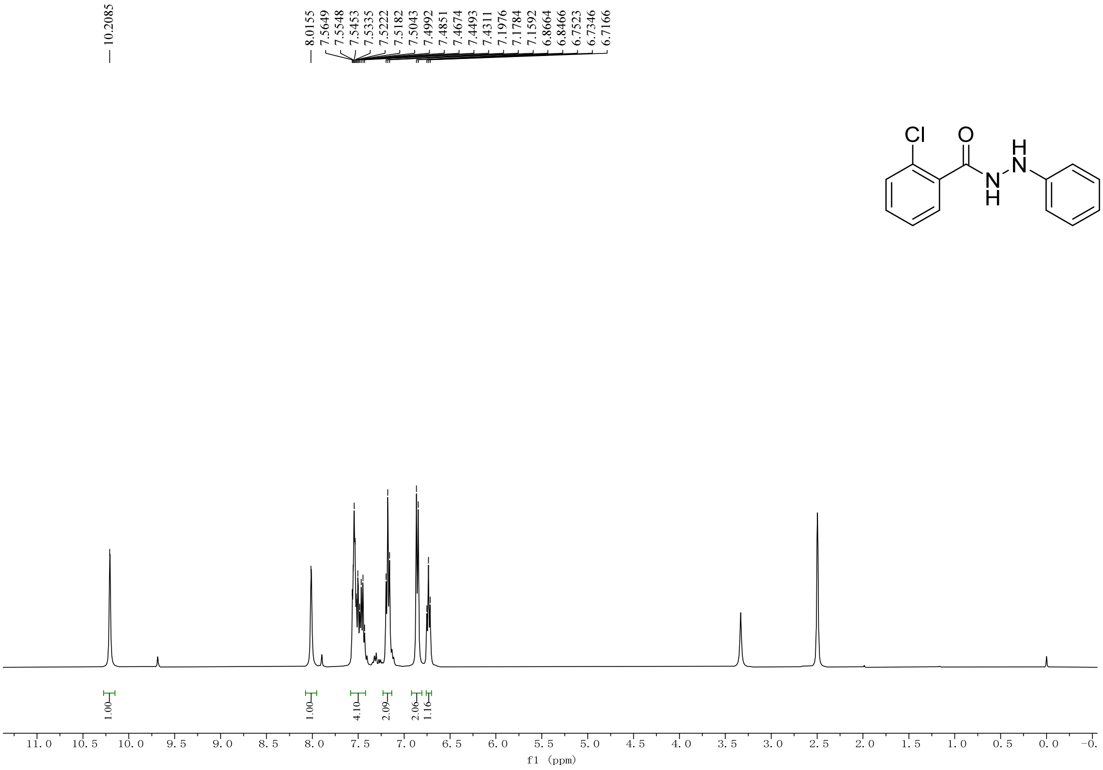

The ¹H NMR spectrum of **A5**.

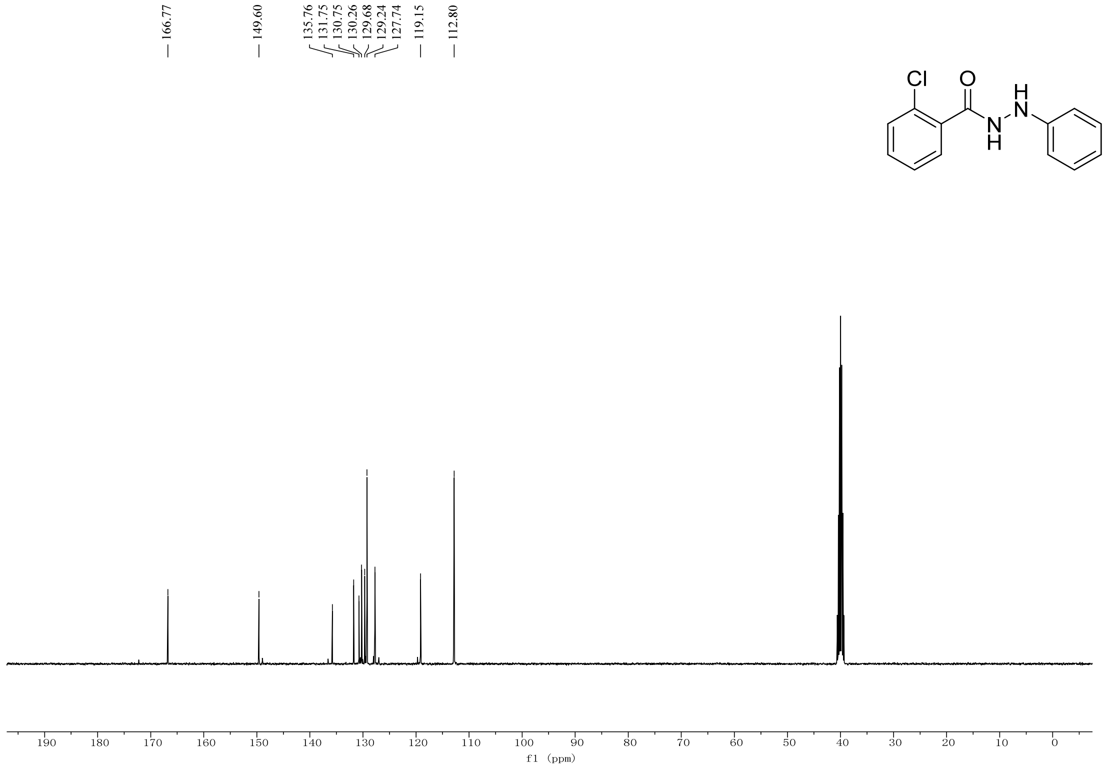

The ¹³C NMR spectrum of **A5**.

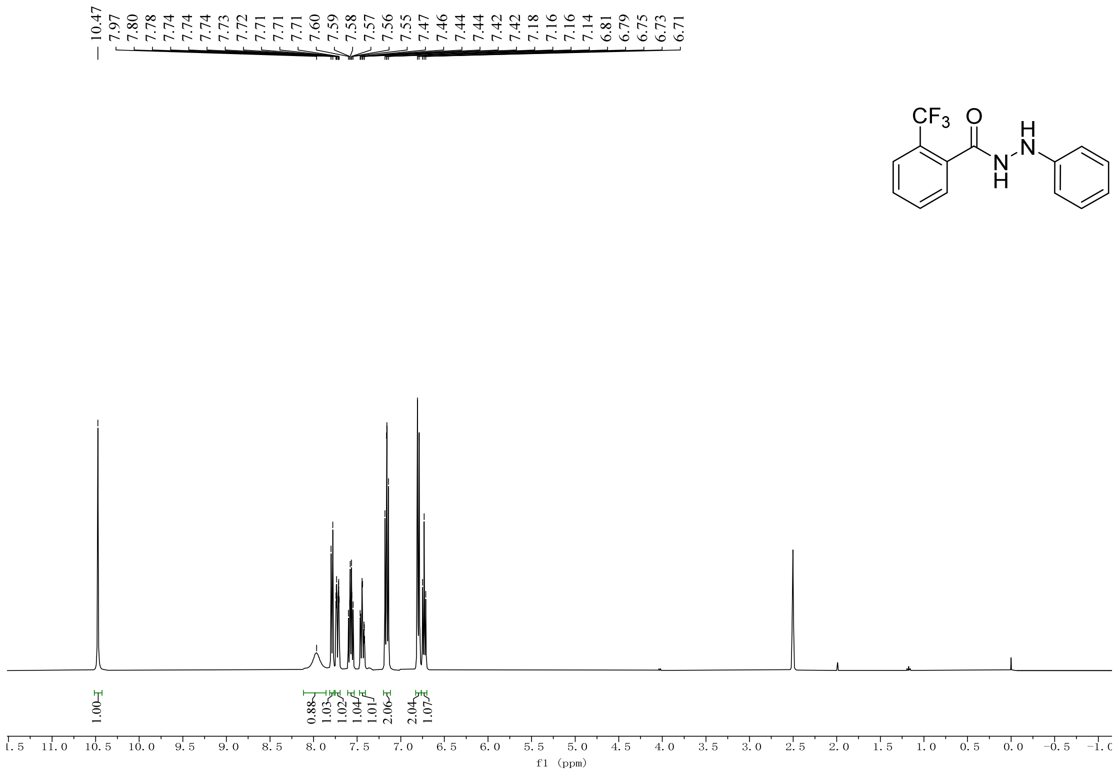

The ^{1}H NMR spectrum of **A6**.

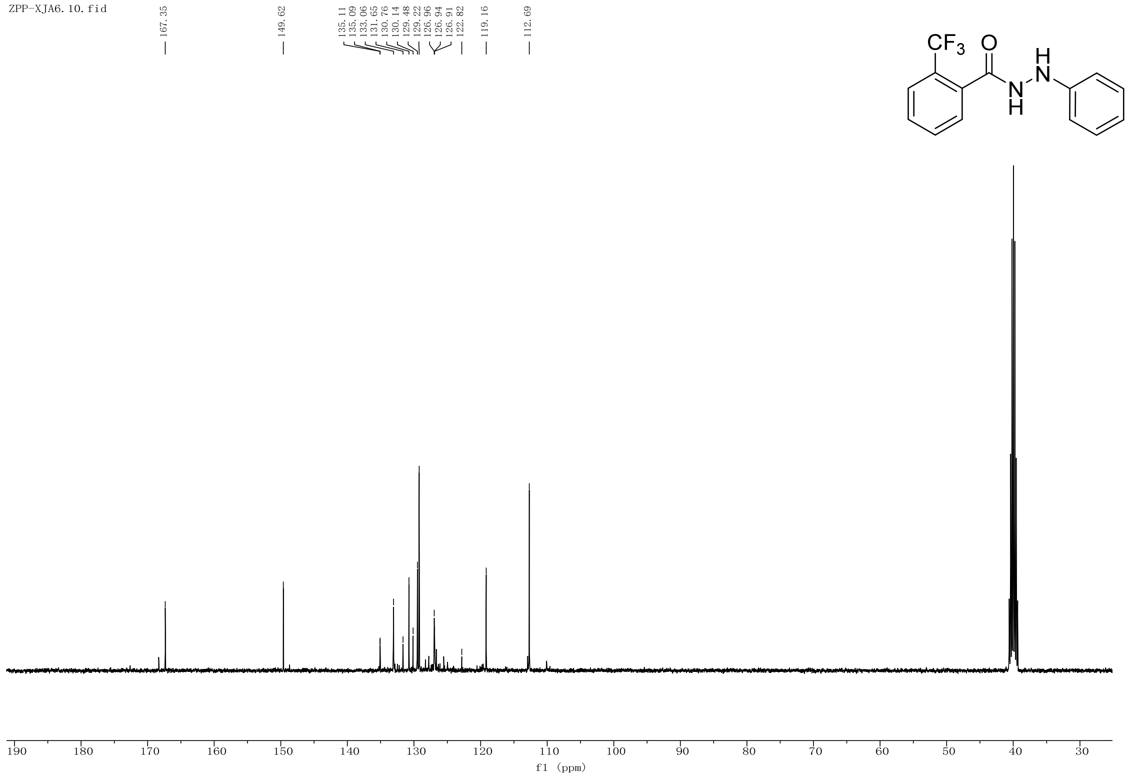

The ^{13}C NMR spectrum of **A6**.

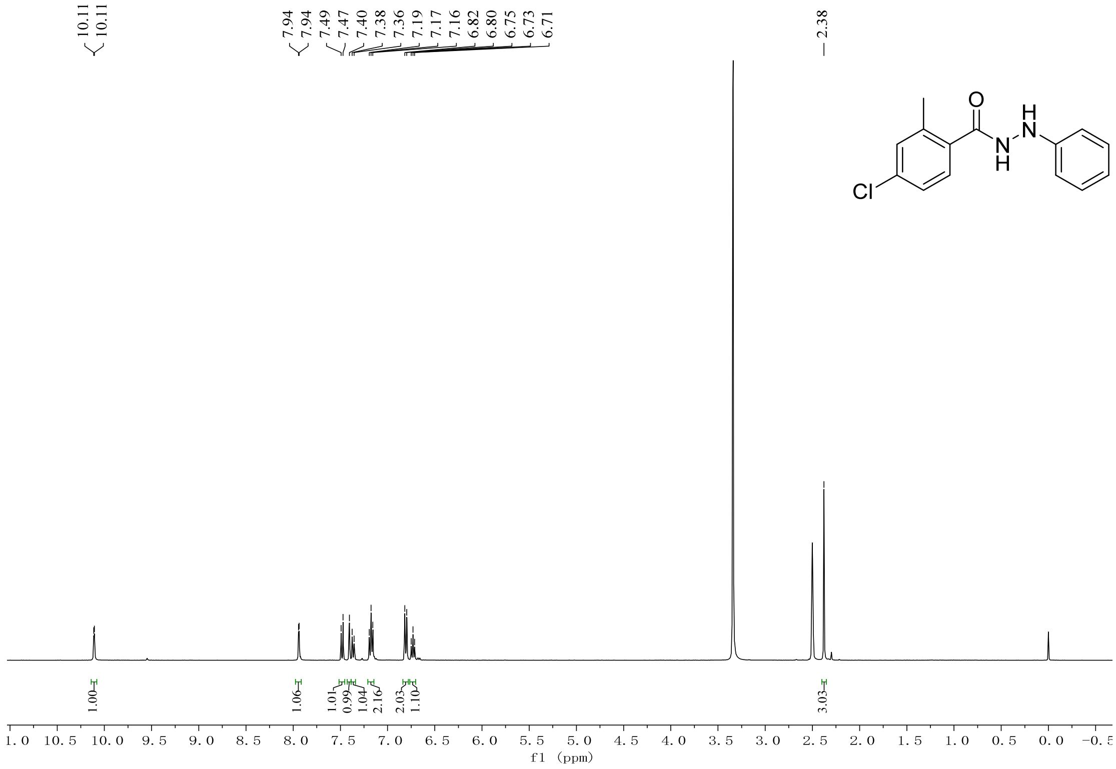

The ^{1}H NMR spectrum of **A**$_7$.

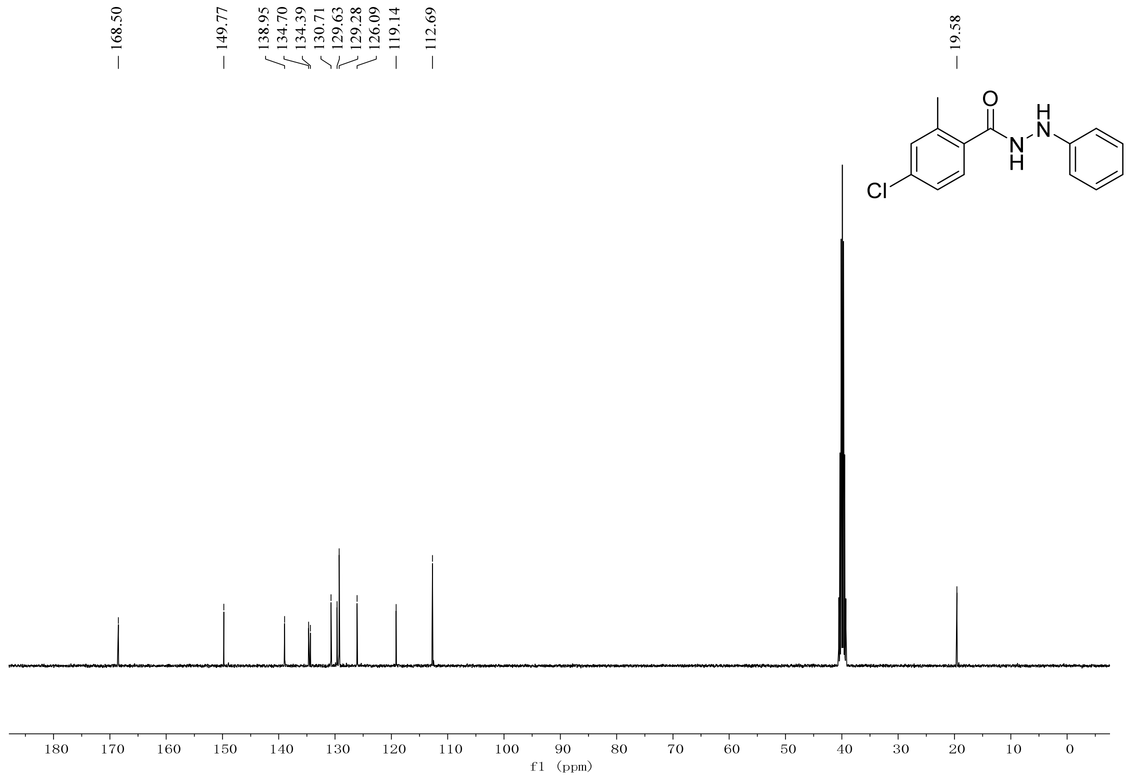

The ^{13}C NMR spectrum of **A**$_7$.

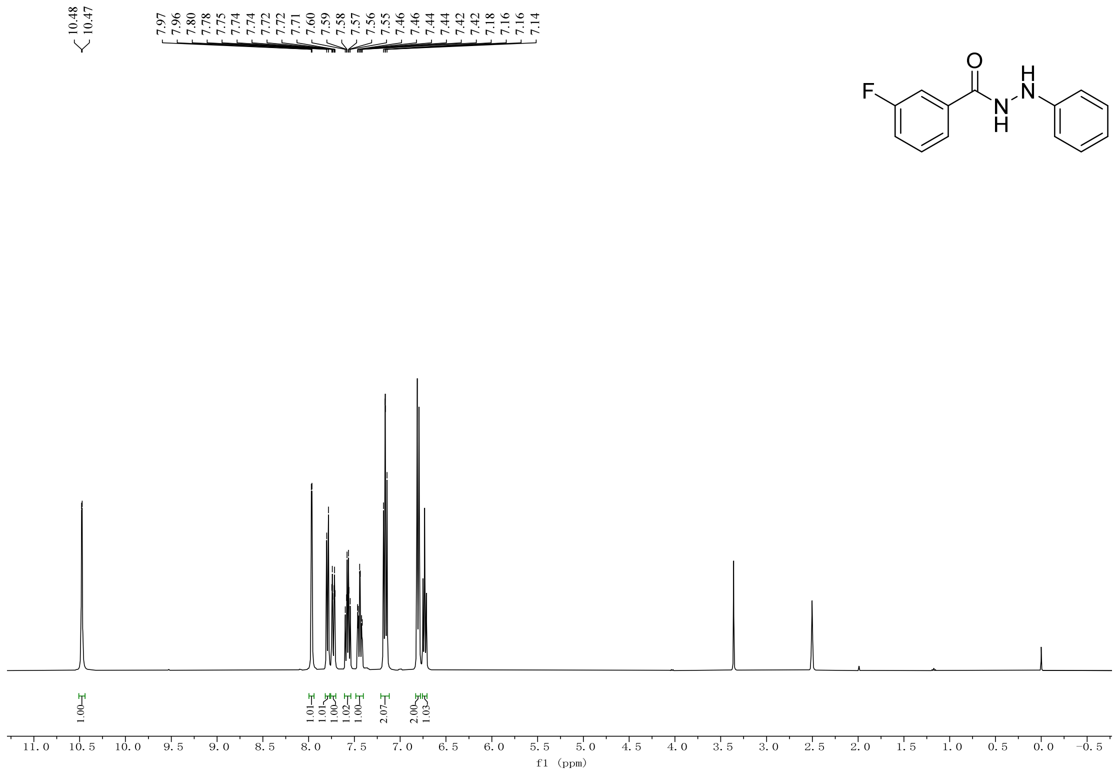

The ^{1}H NMR spectrum of **A**8.

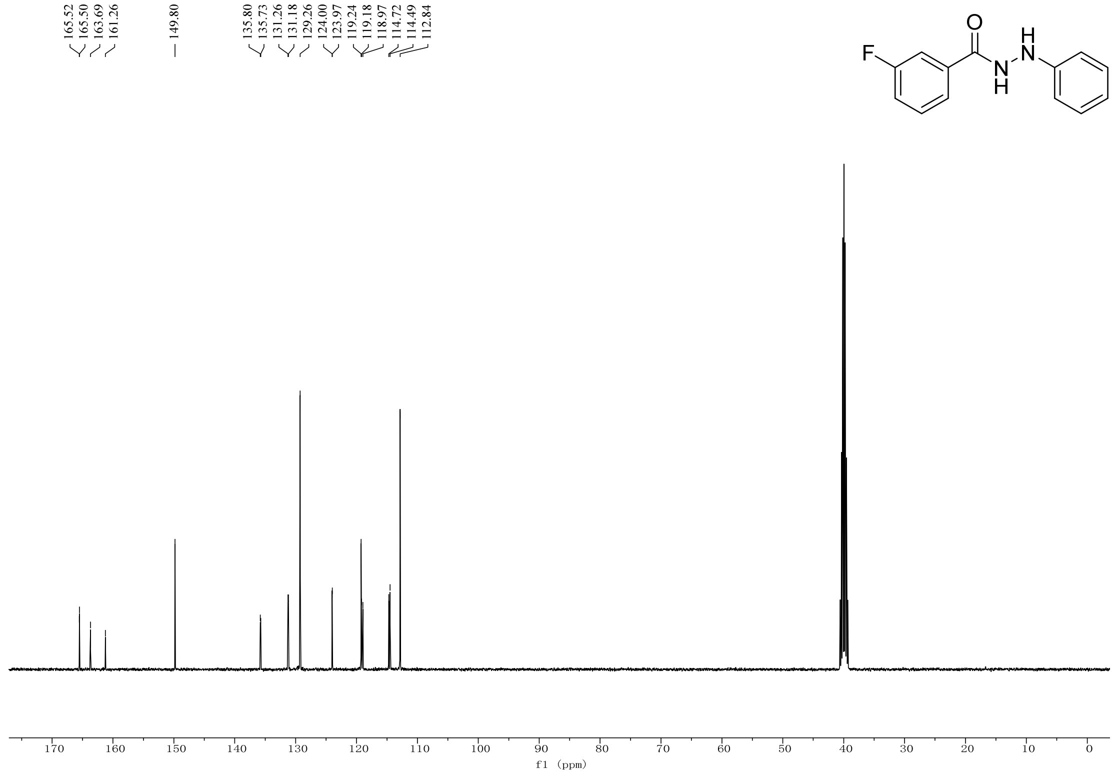

The ^{13}C NMR spectrum of **A**8.

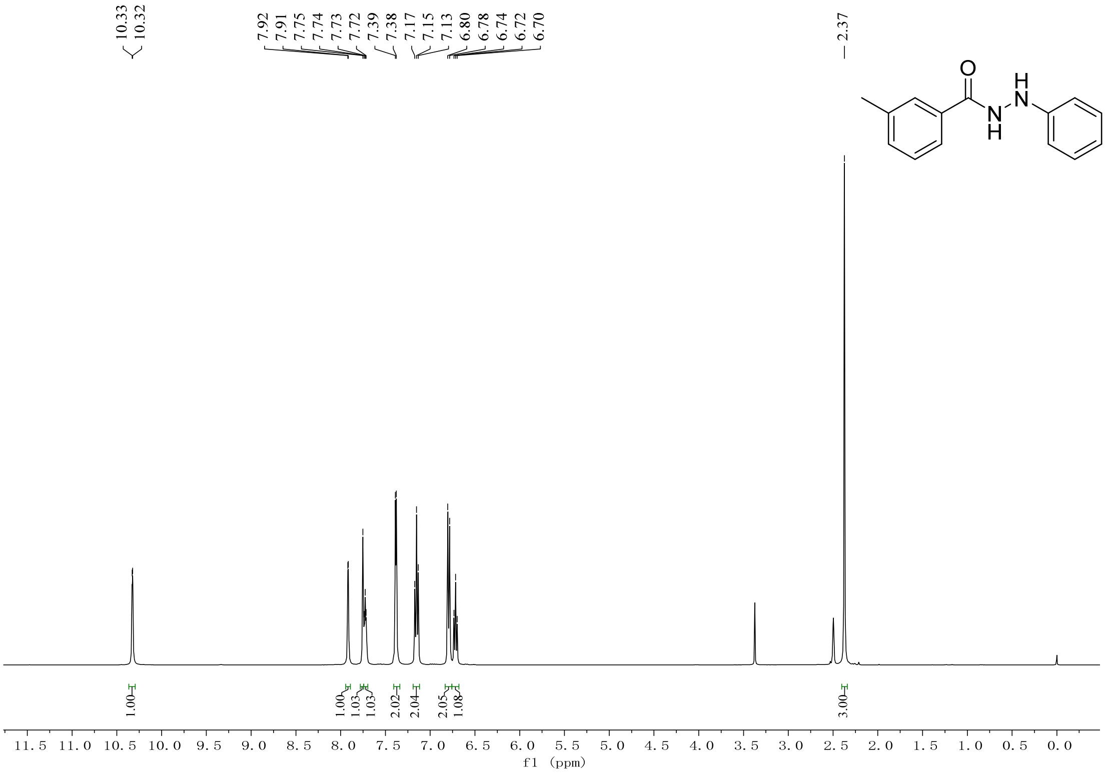

The ¹H NMR spectrum of **A9**.

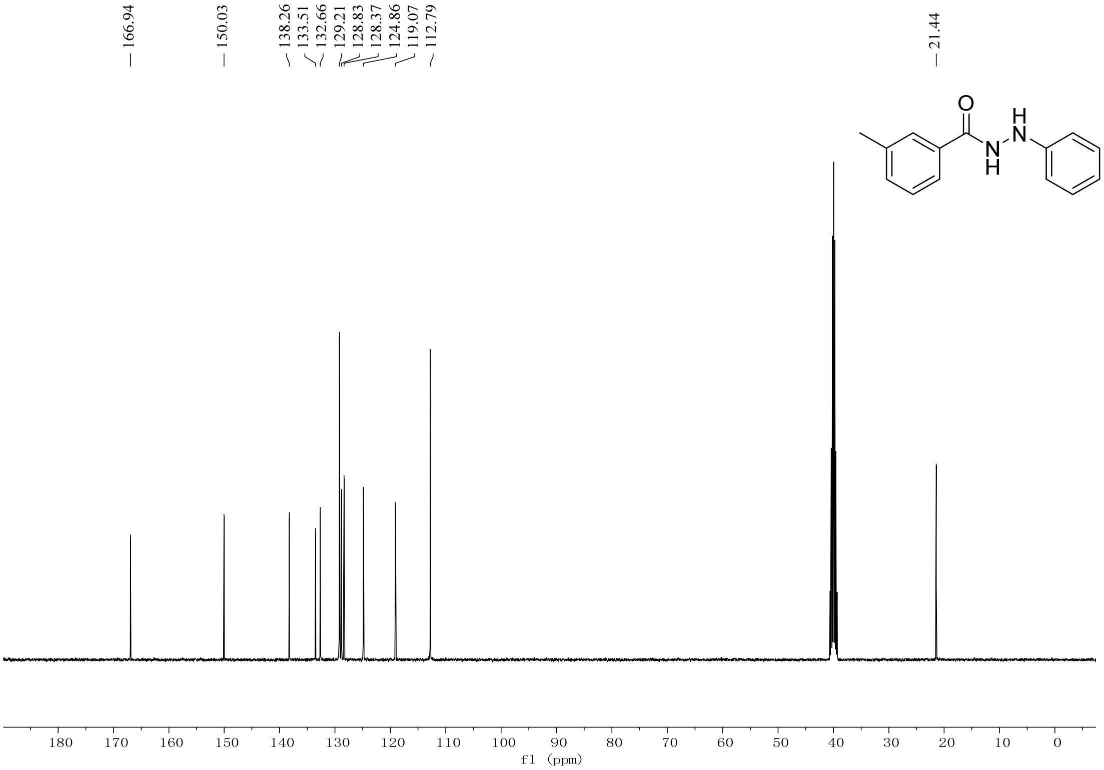

The ¹³C NMR spectrum of **A9**.

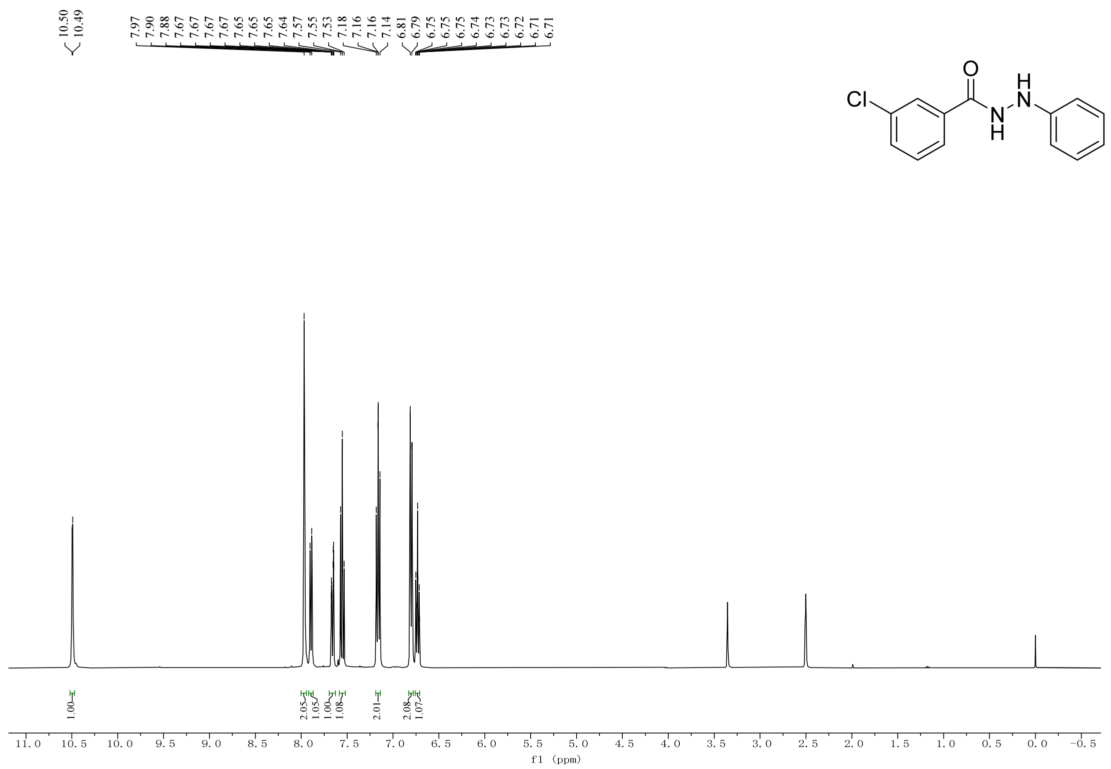

The ^{1}H NMR spectrum of **A10**.

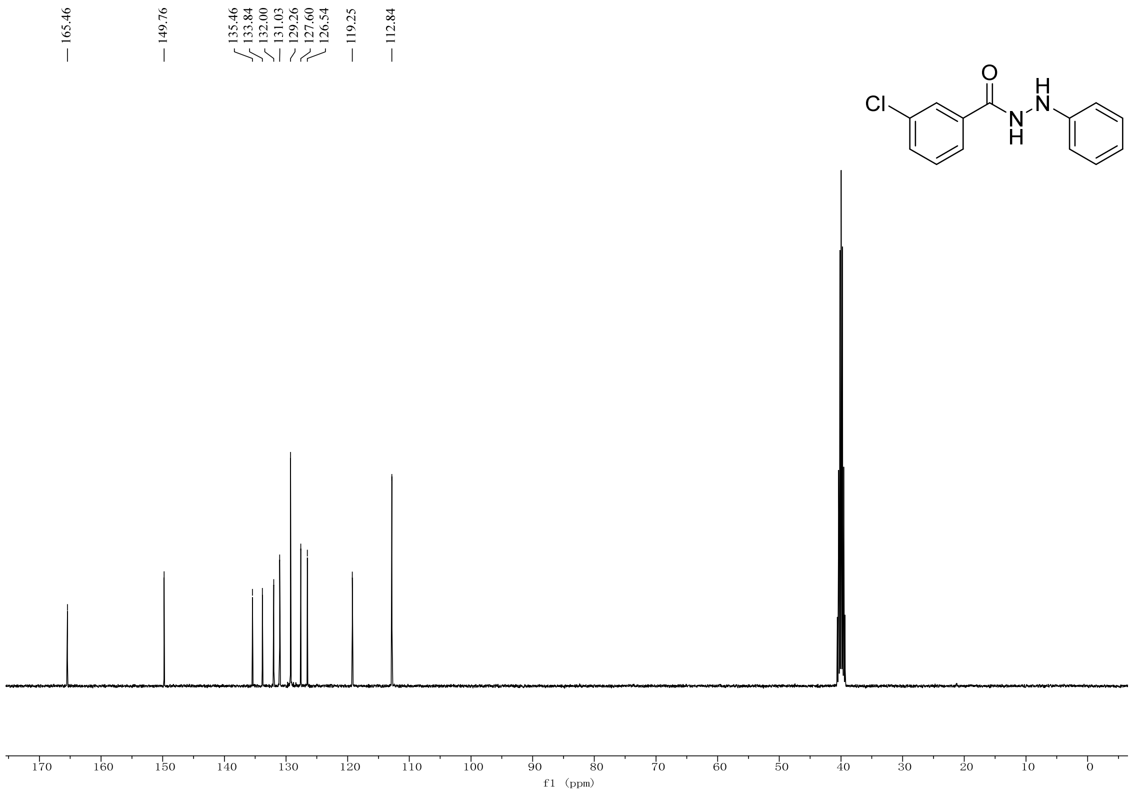

The ^{13}C NMR spectrum of **A10**.

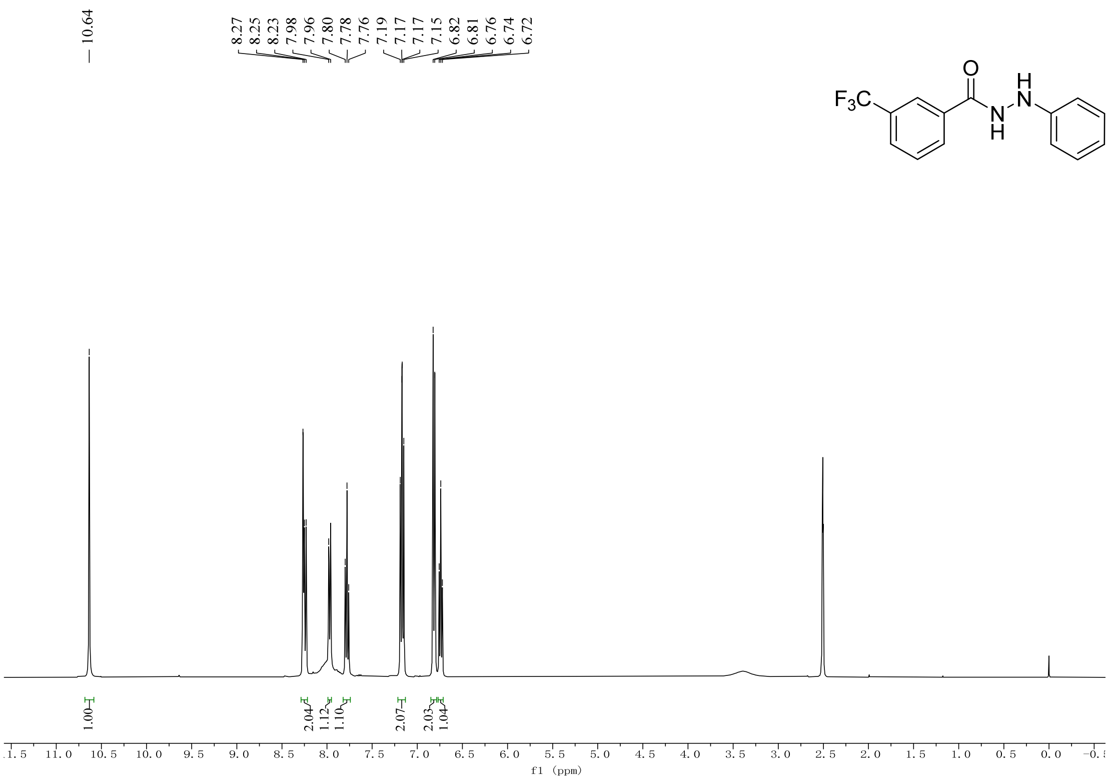

The ^{1}H NMR spectrum of **A11**.

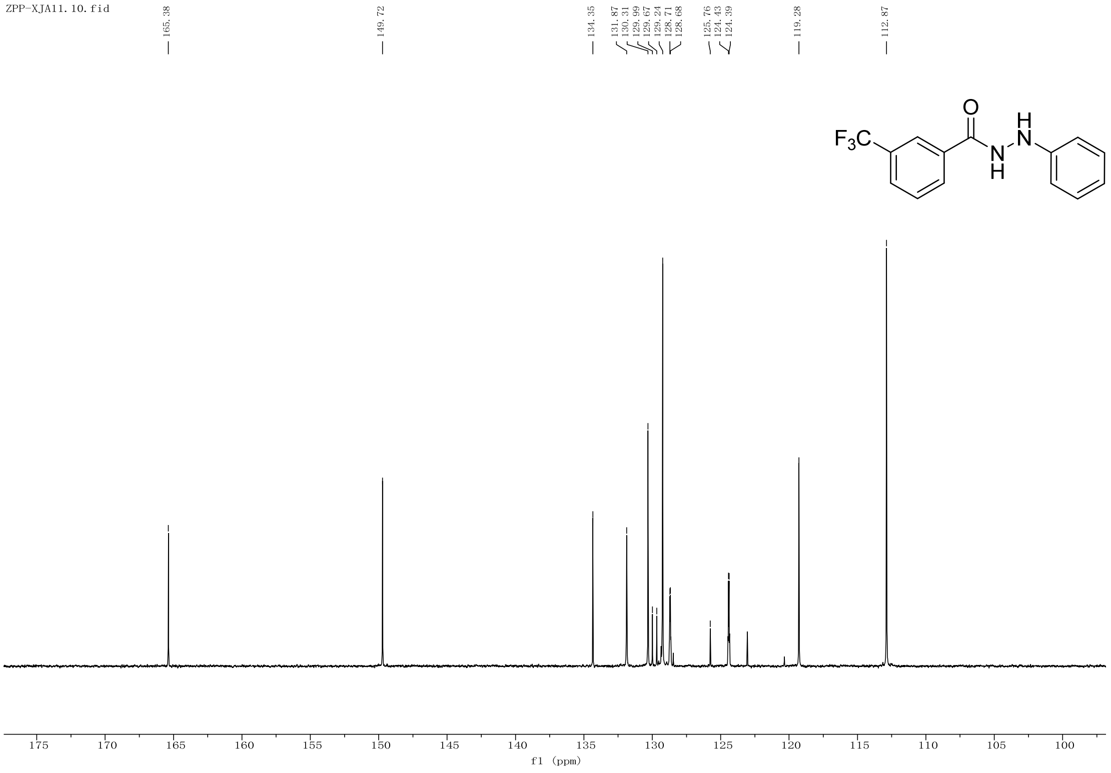

The ^{13}C NMR spectrum of **A11**.

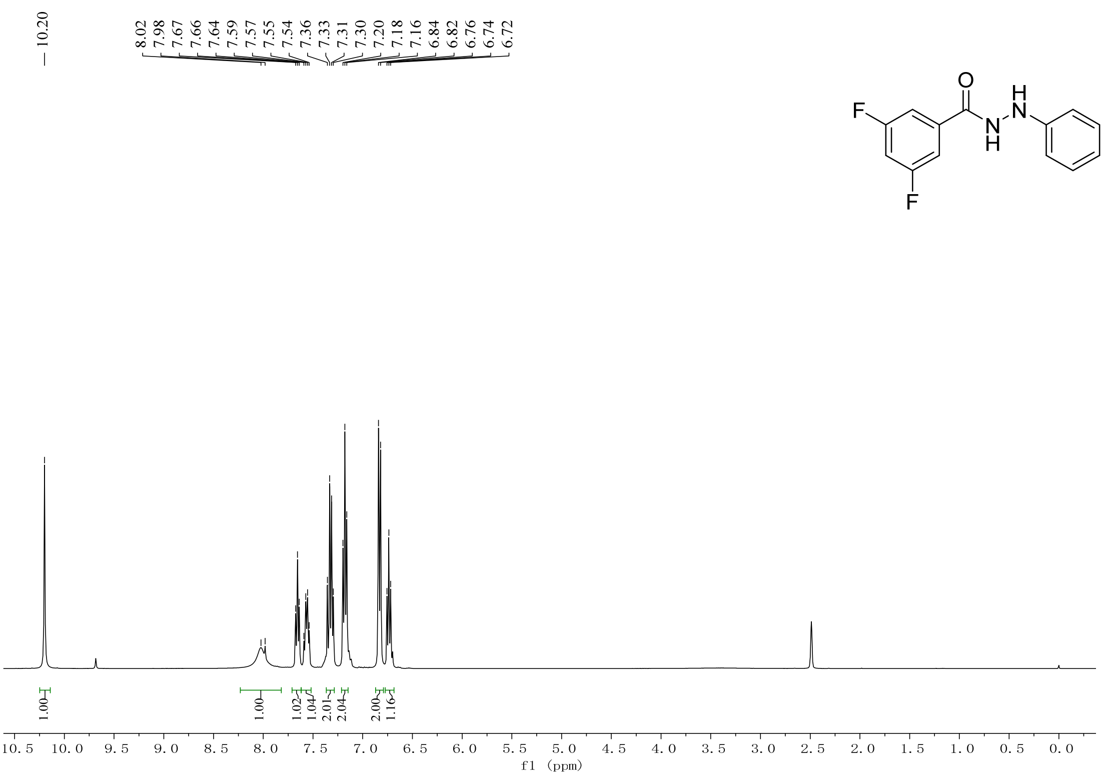

The ¹H NMR spectrum of **A12**.

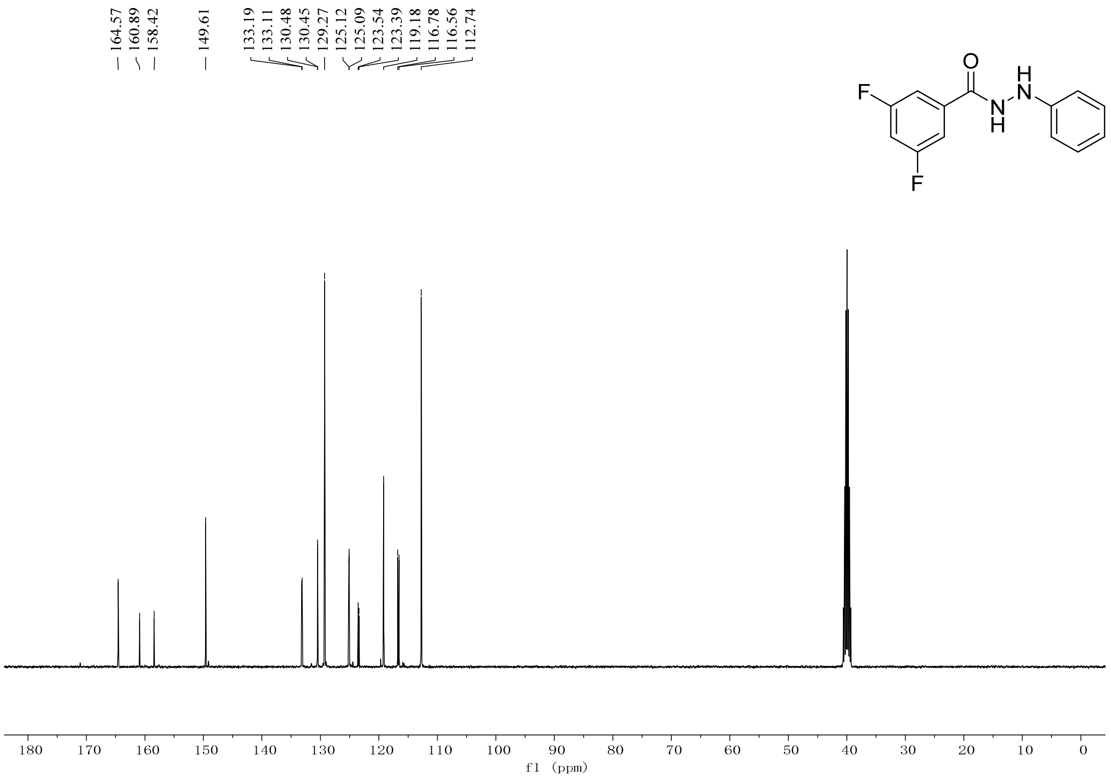

The ¹³C NMR spectrum of **A12**.

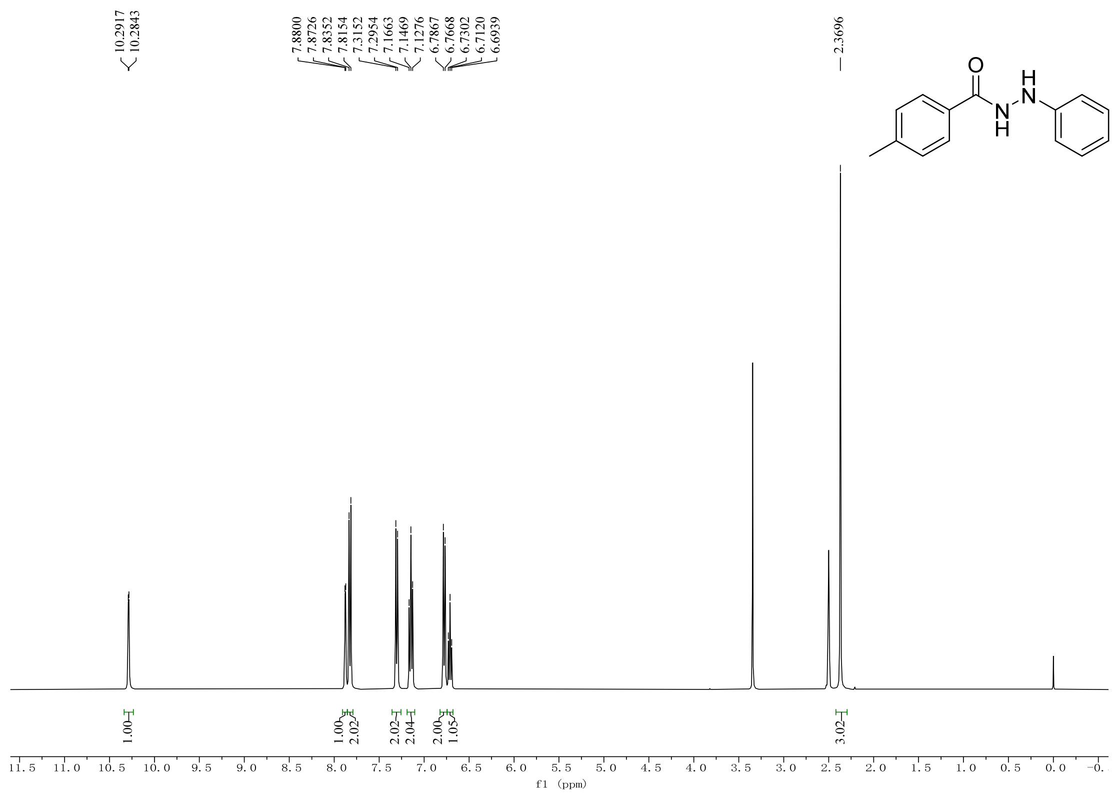

The ^{1}H NMR spectrum of **A13**.

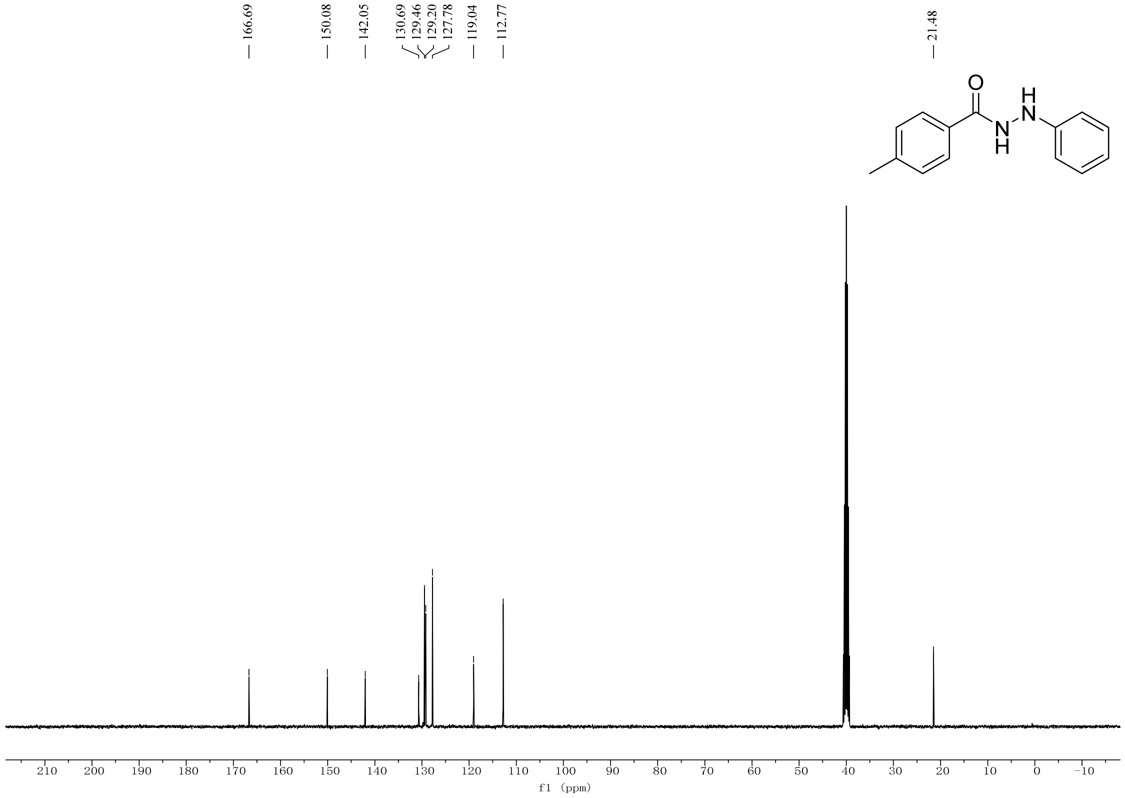

The ^{13}C NMR spectrum of **A13**.

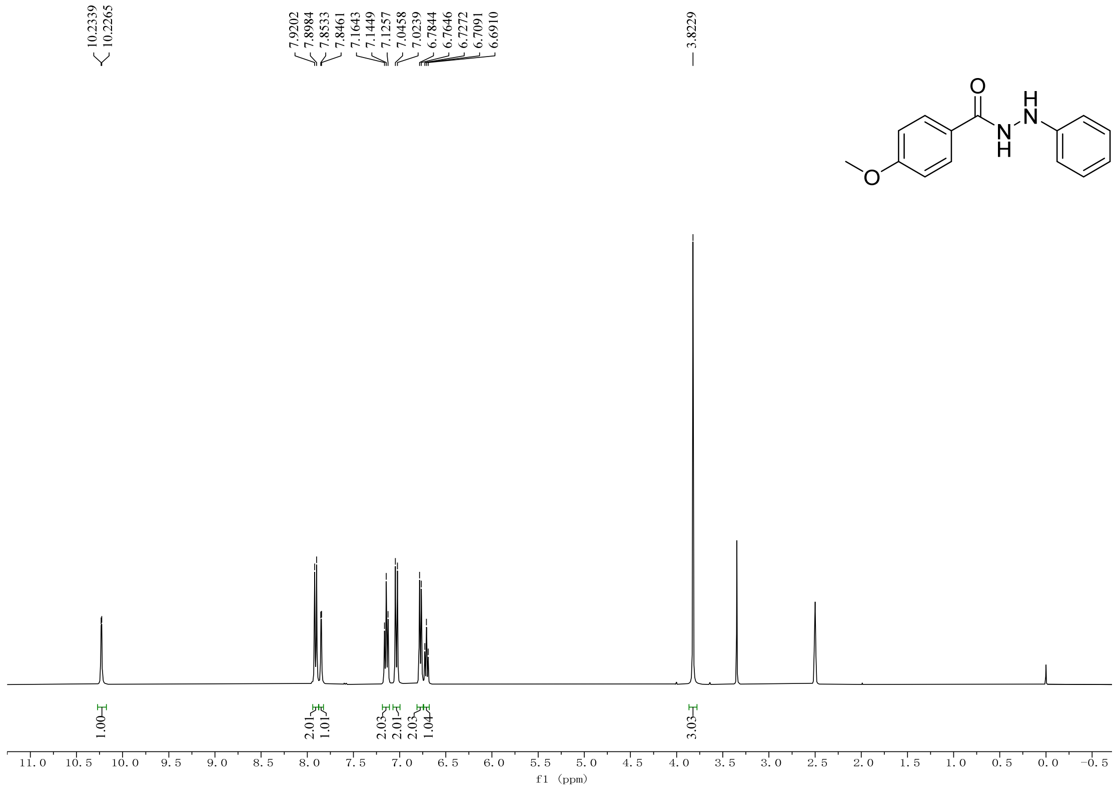

The ^{1}H NMR spectrum of **A14**.

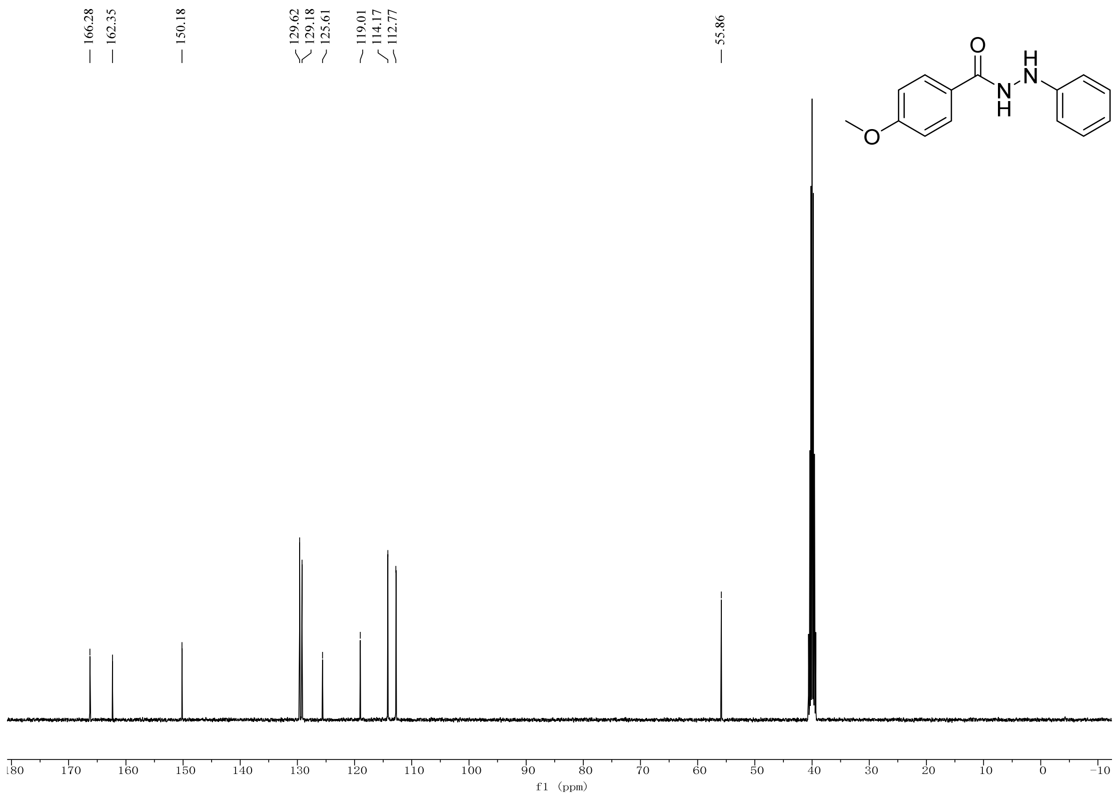

The ^{13}C NMR spectrum of **A14**.

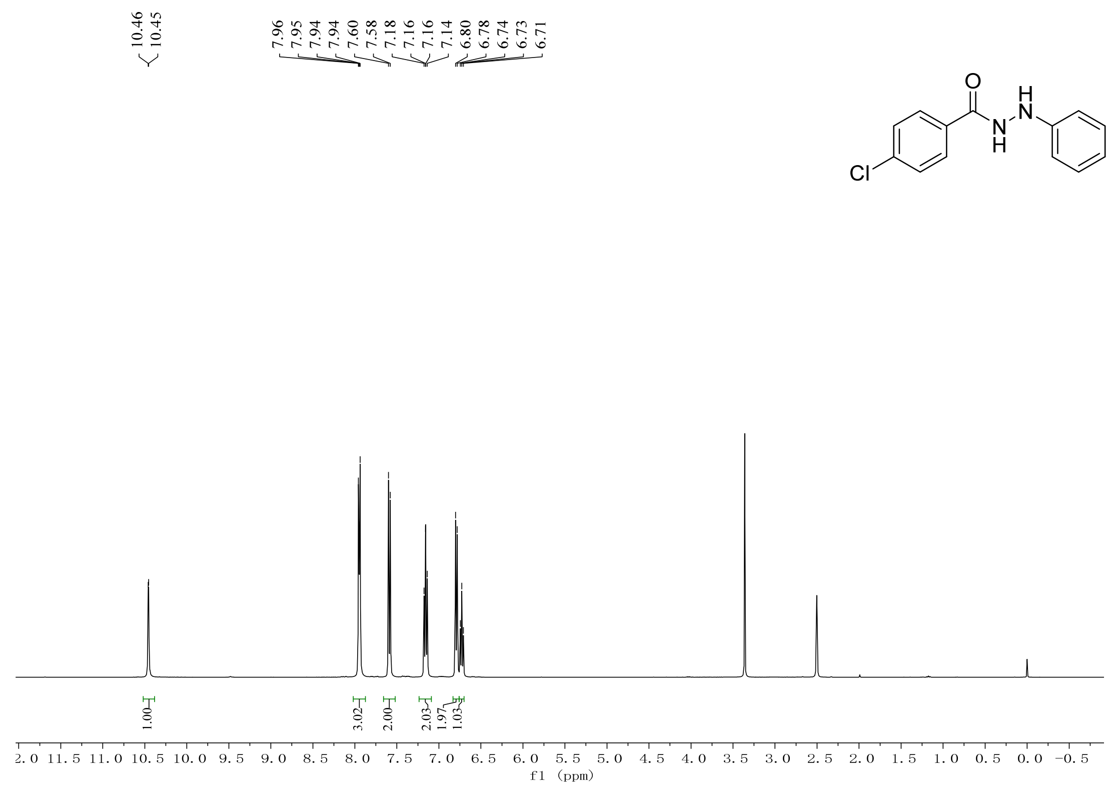

The ^{1}H NMR spectrum of **A15**.

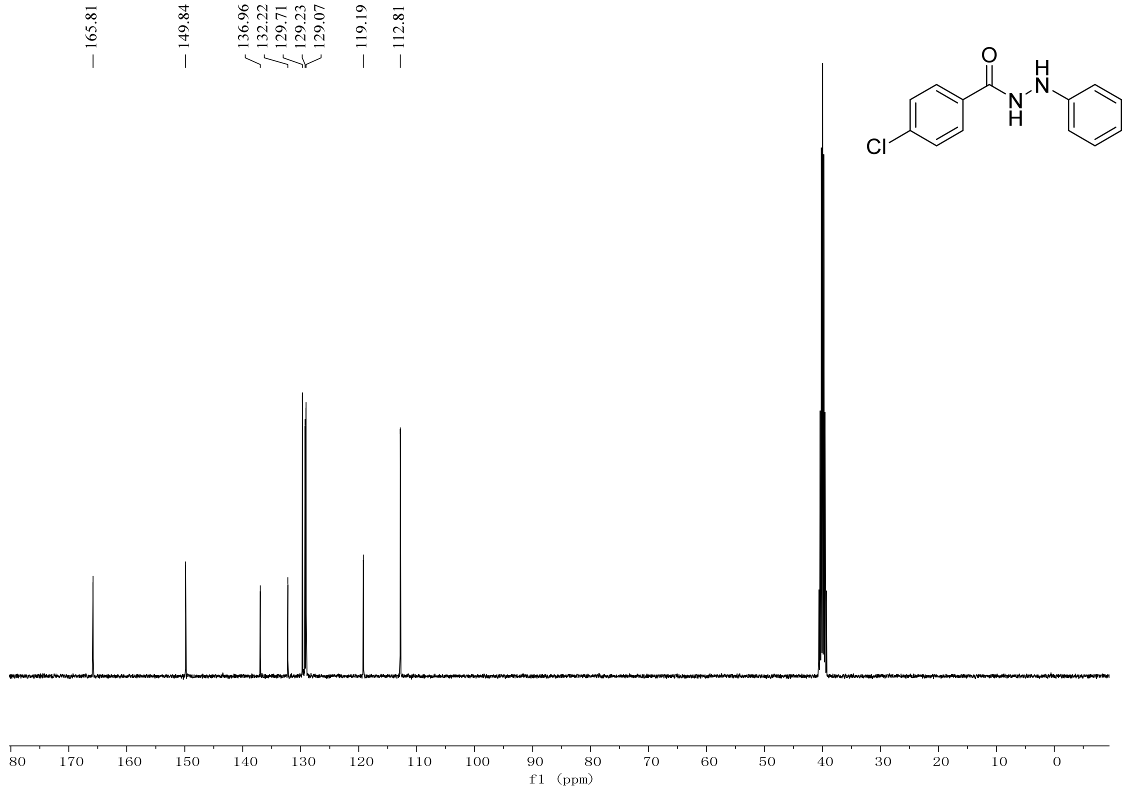

The ^{13}C NMR spectrum of **A15**.

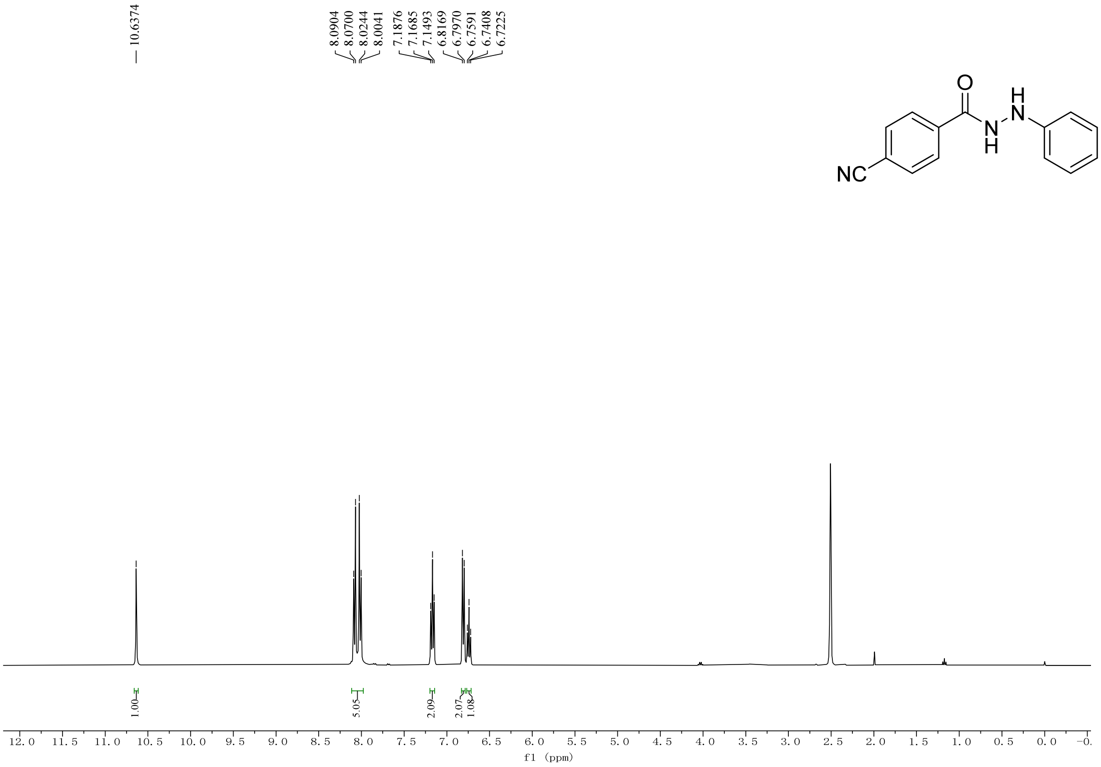

The ¹H NMR spectrum of **A16**.

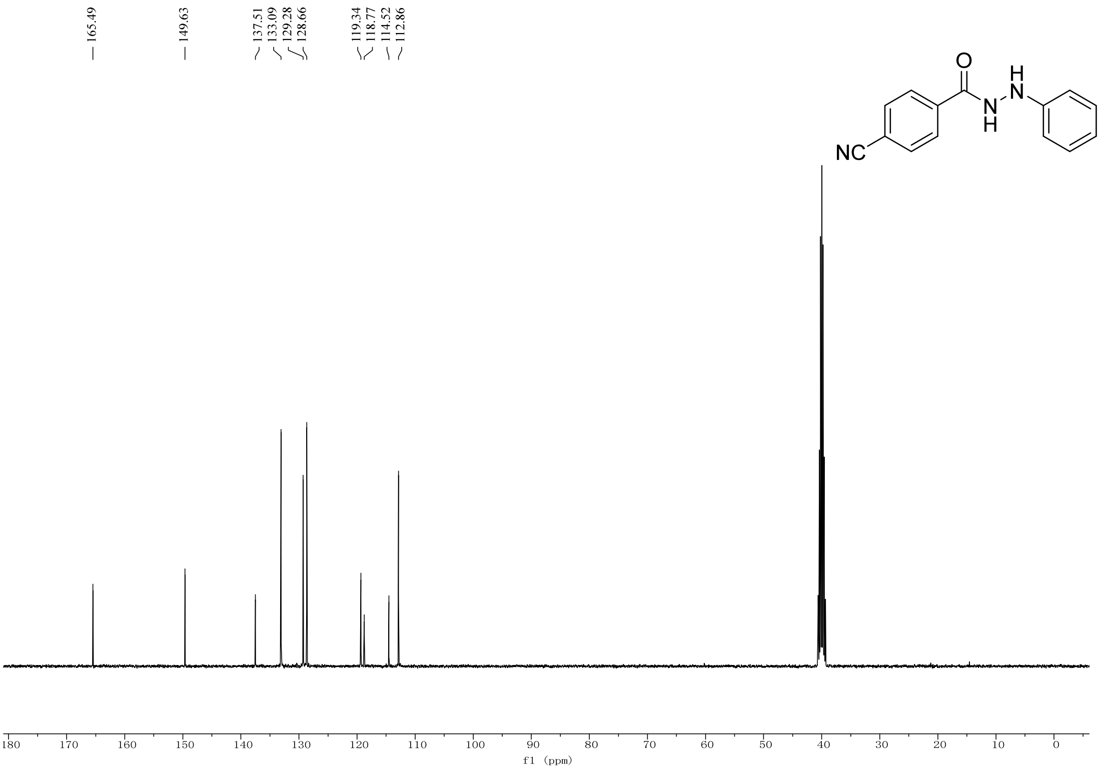

The ¹³C NMR spectrum of **A16**.

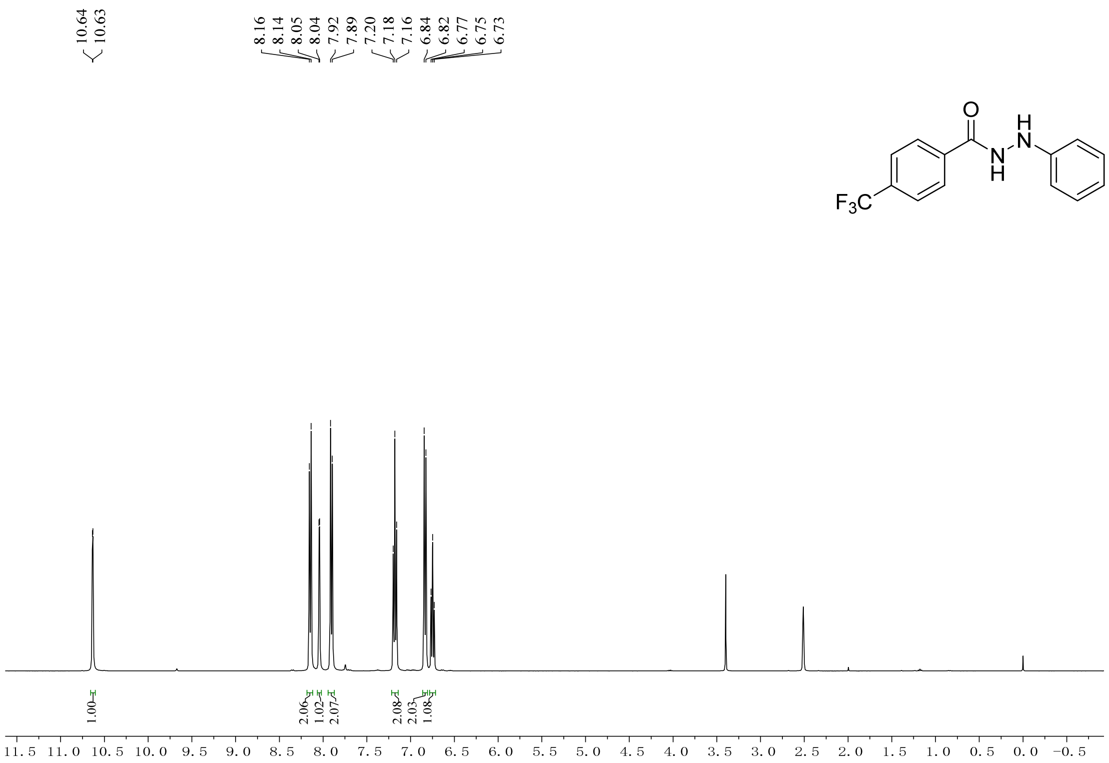

The ^{1}H NMR spectrum of **A**17.

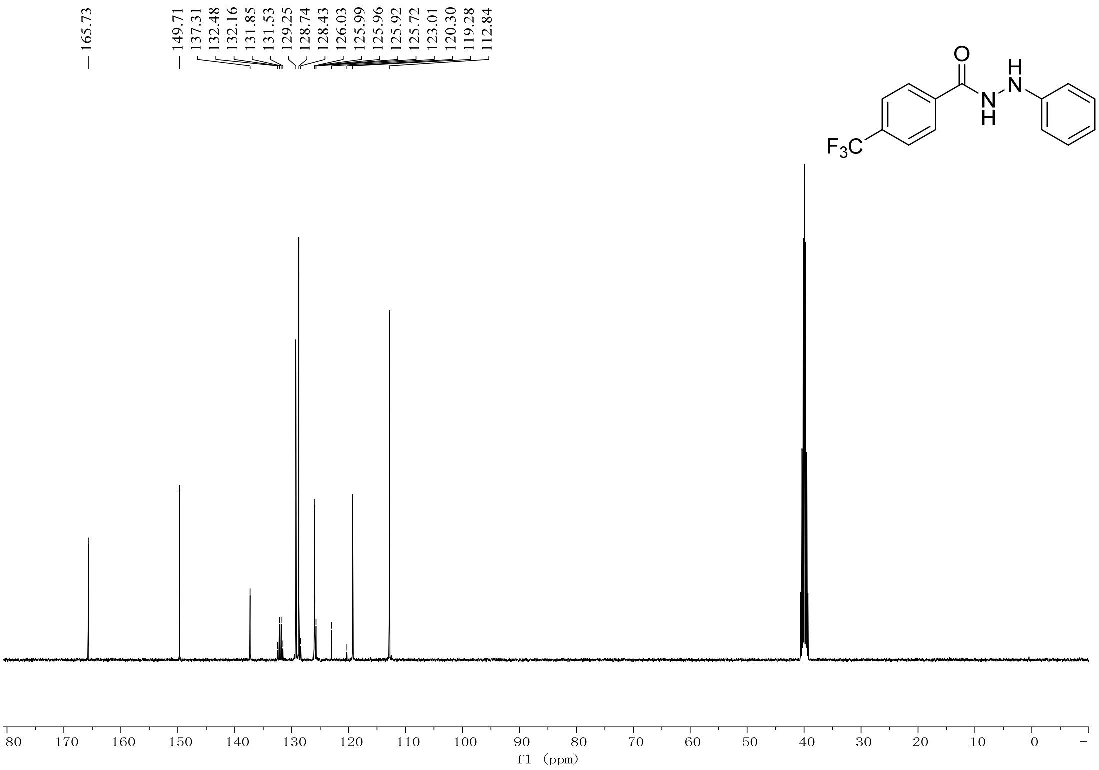

The ^{13}C NMR spectrum of **A**17.

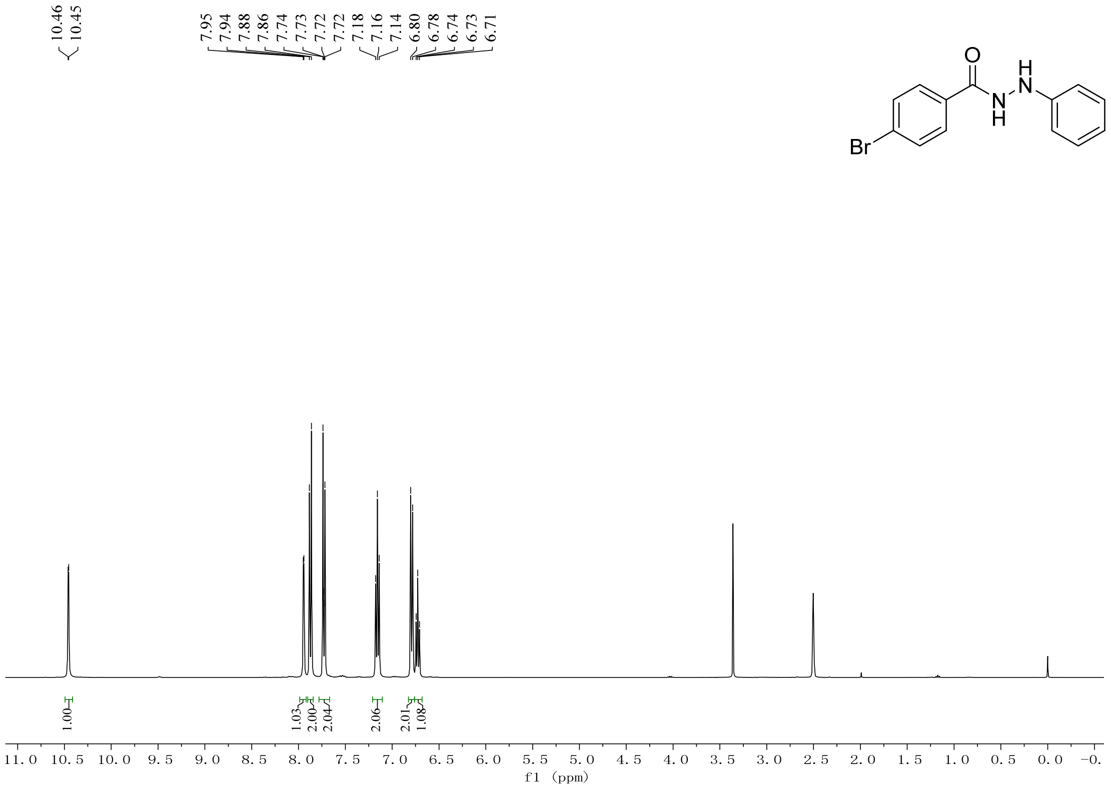

The ^{1}H NMR spectrum of **A18**.

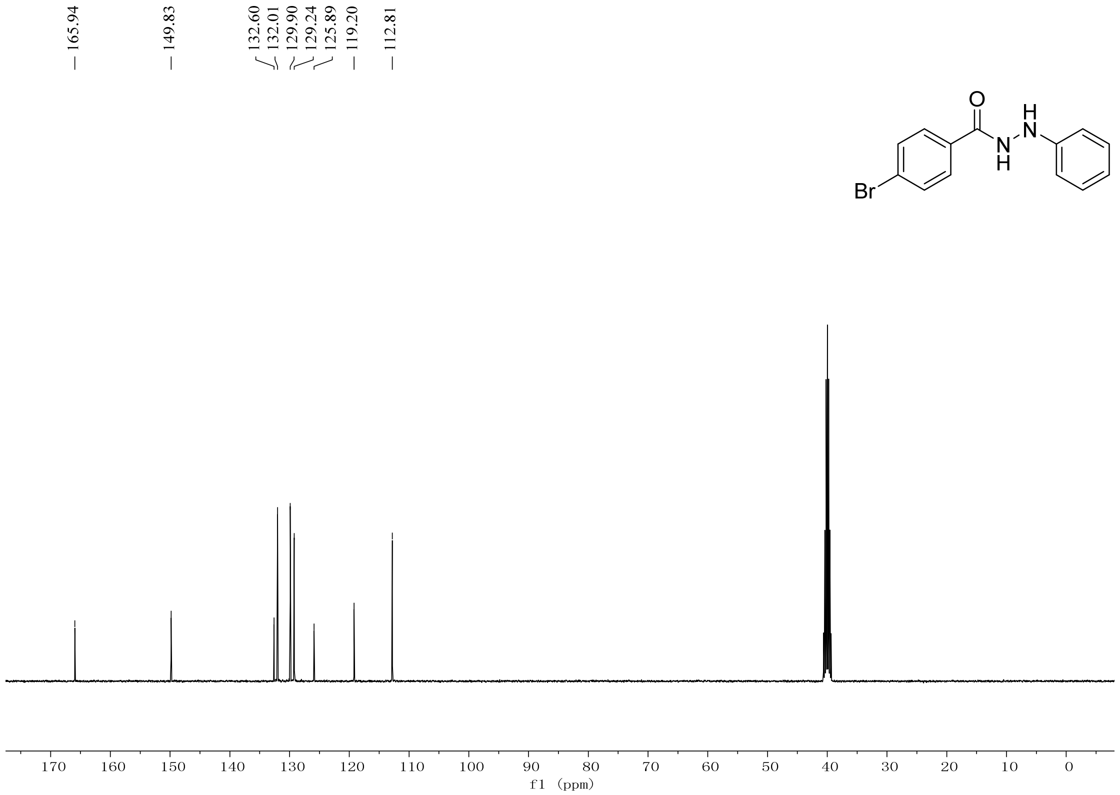

The ^{13}C NMR spectrum of **A18**.

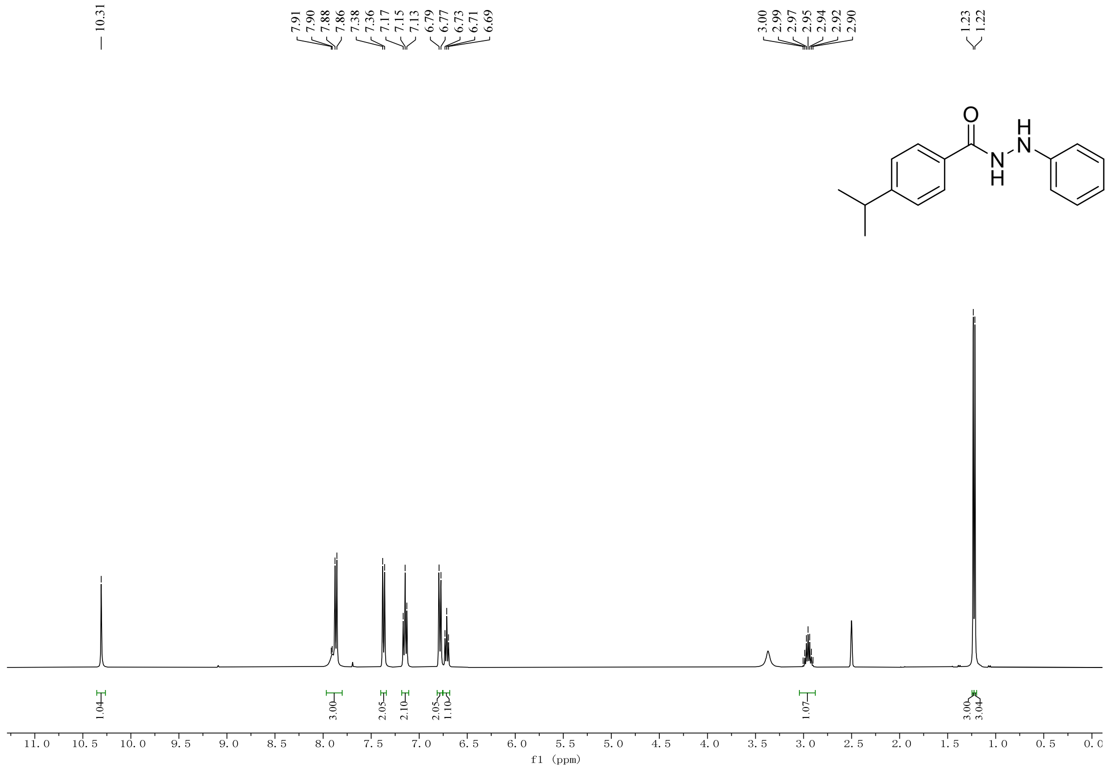

The ¹H NMR spectrum of **A19**.

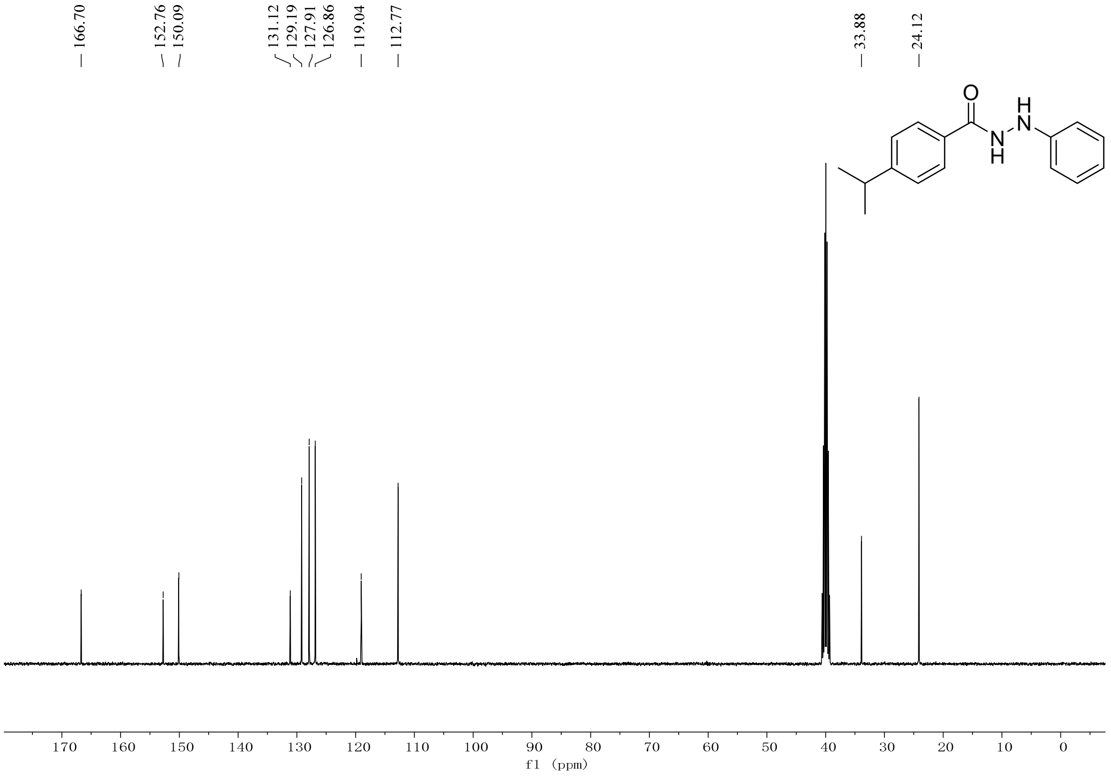

The ¹³C NMR spectrum of **A19**.

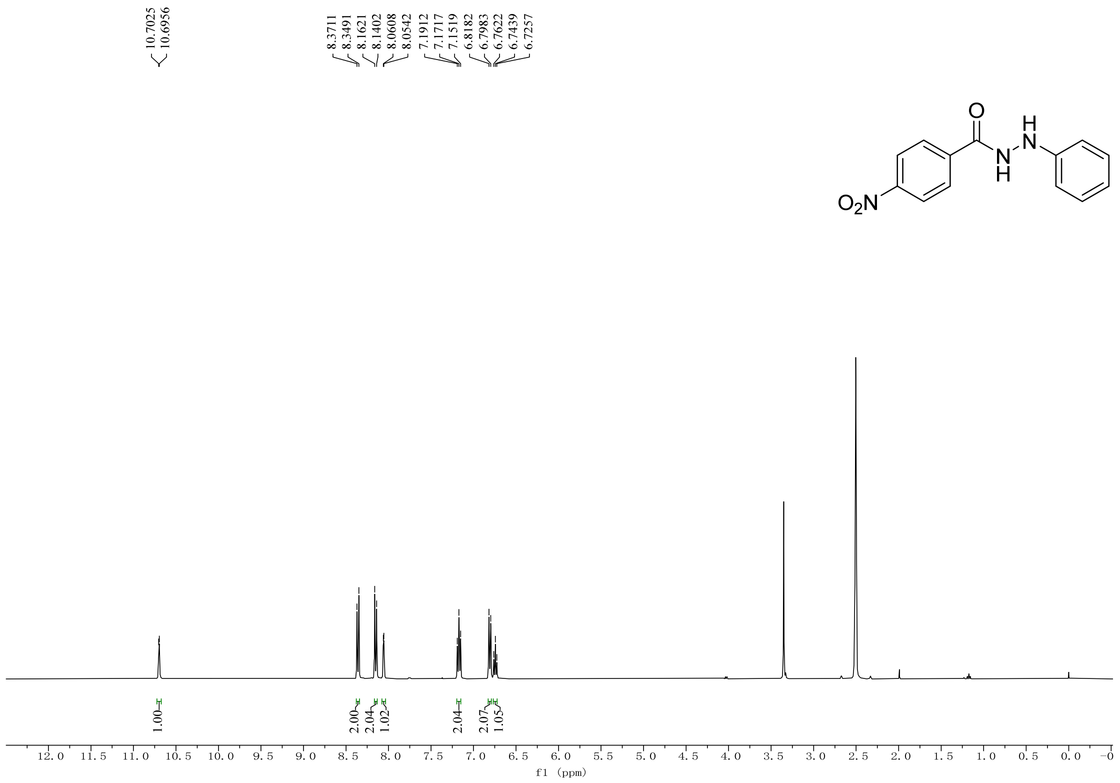

The ^{1}H NMR spectrum of **A20**.

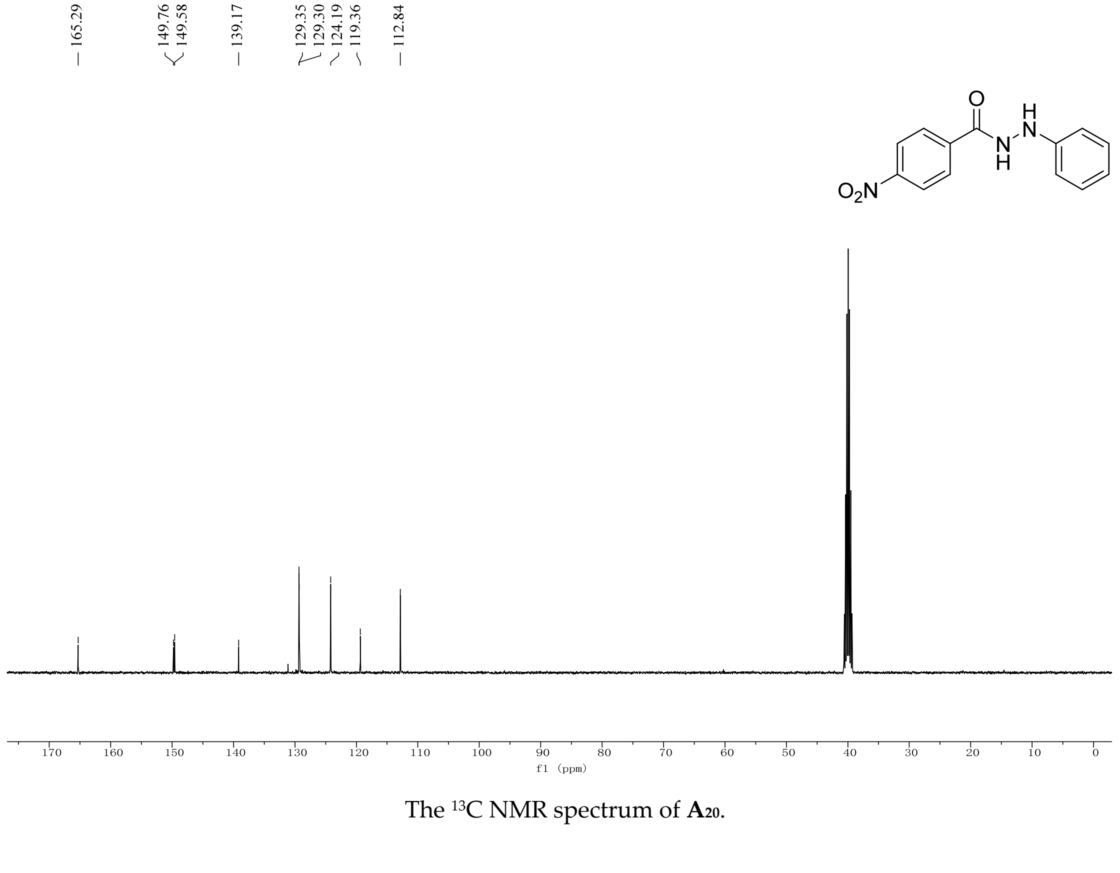

The ^{13}C NMR spectrum of **A20**.

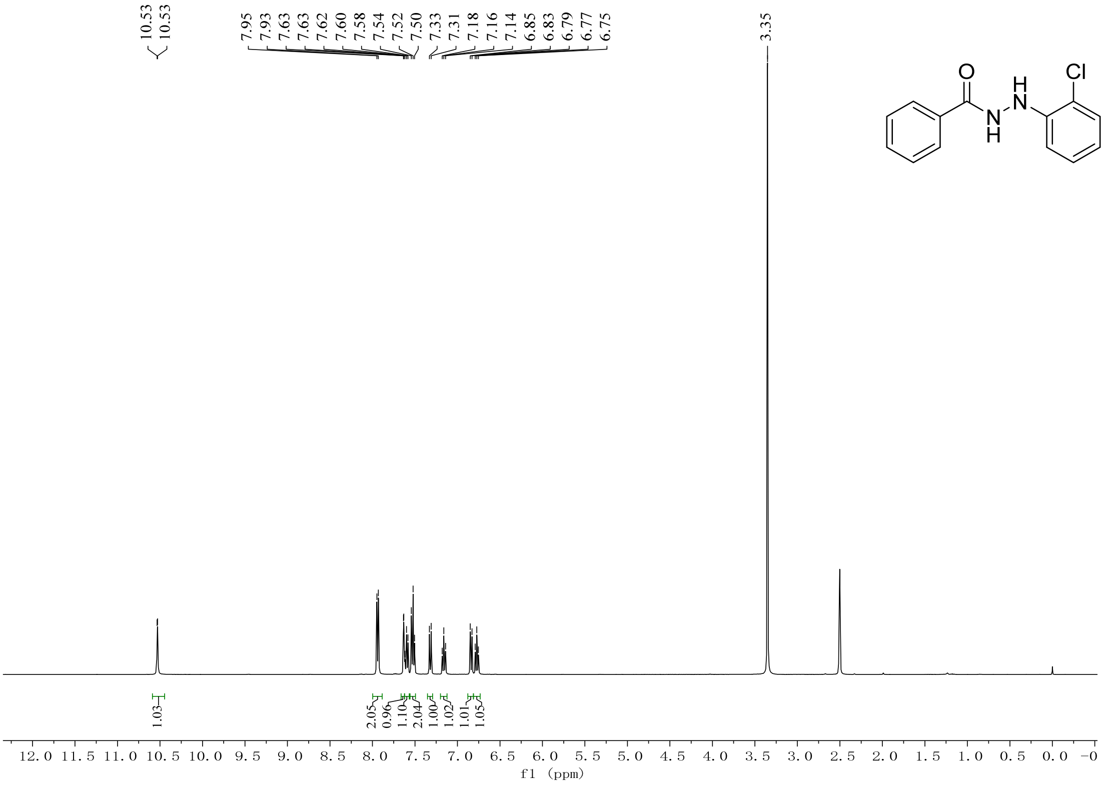

The ^{1}H NMR spectrum of **B1**.

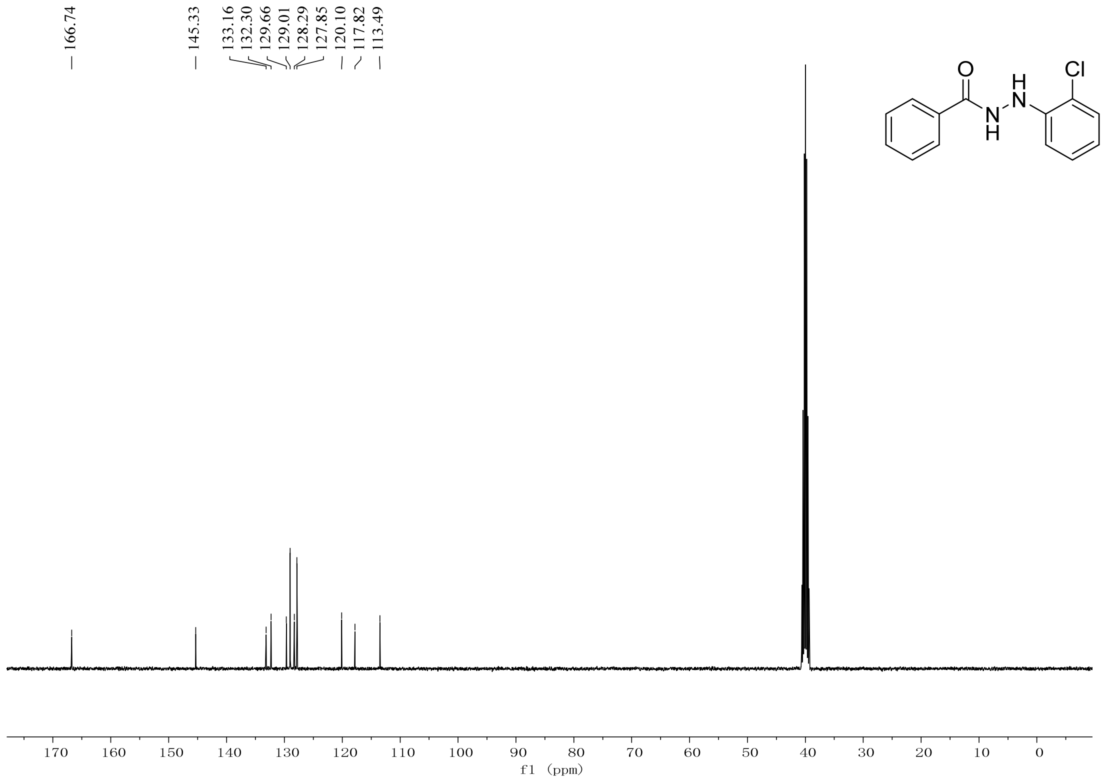

The ^{13}C NMR spectrum of **B1**.

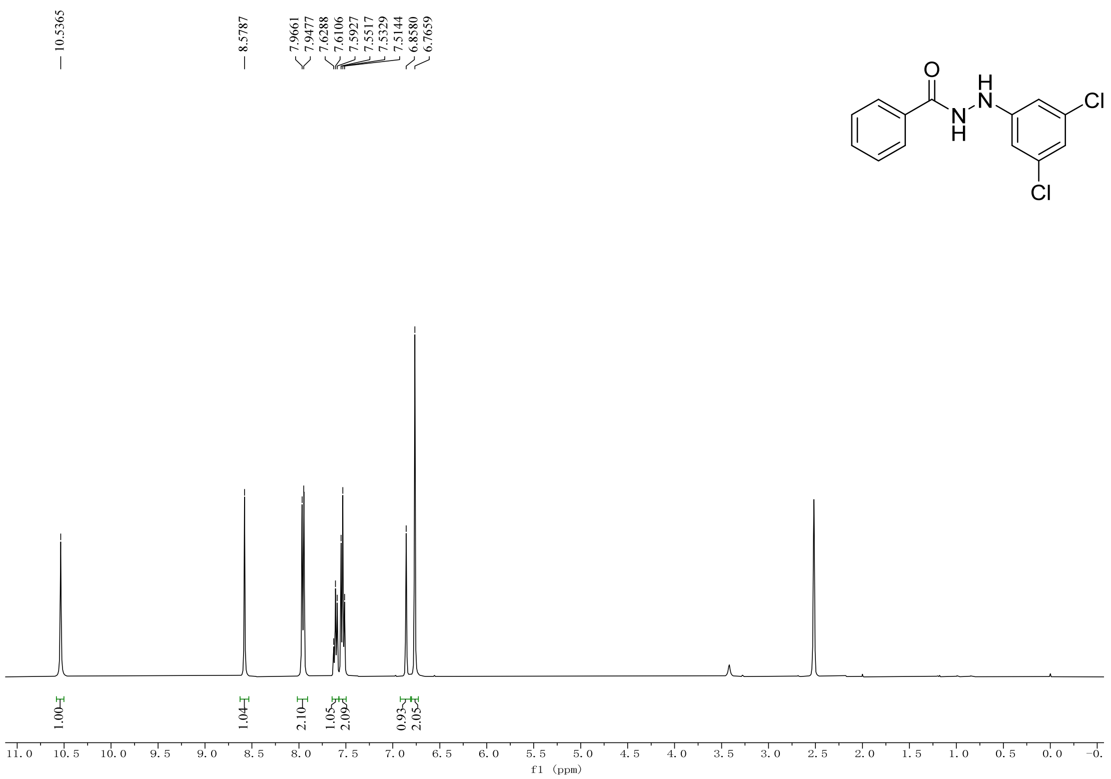

The ^{1}H NMR spectrum of **B2**.

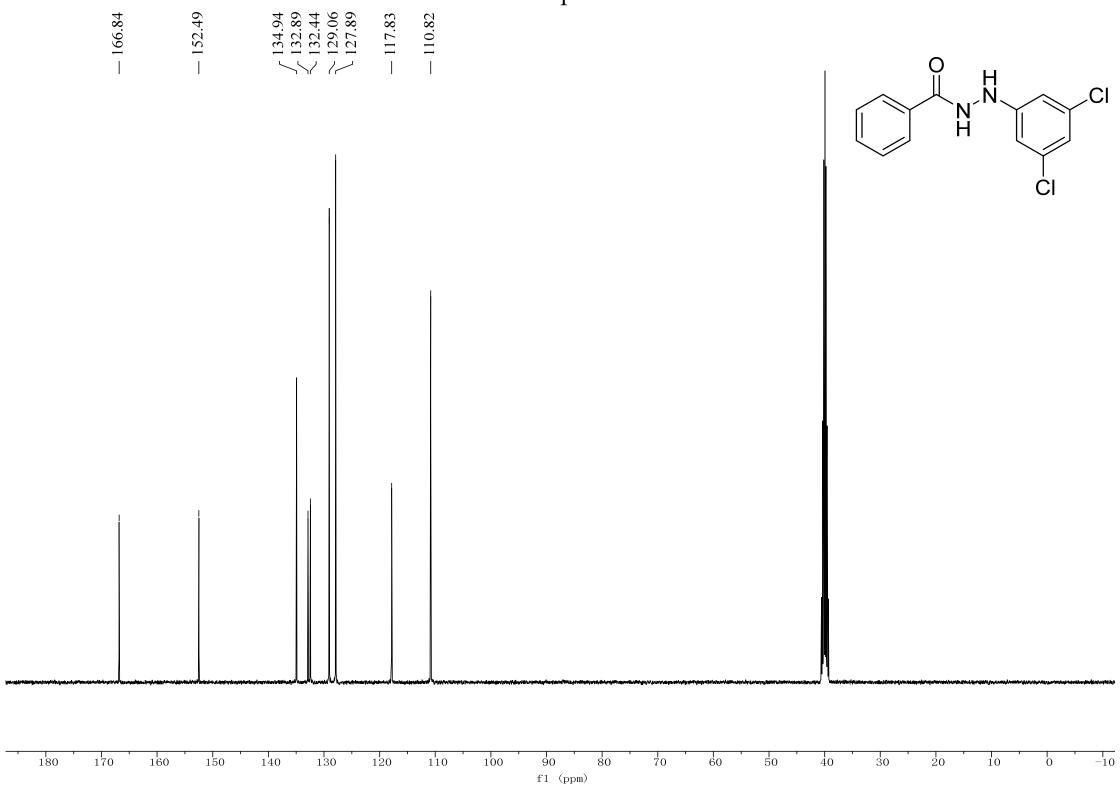

The ^{13}C NMR spectrum of **B2**.

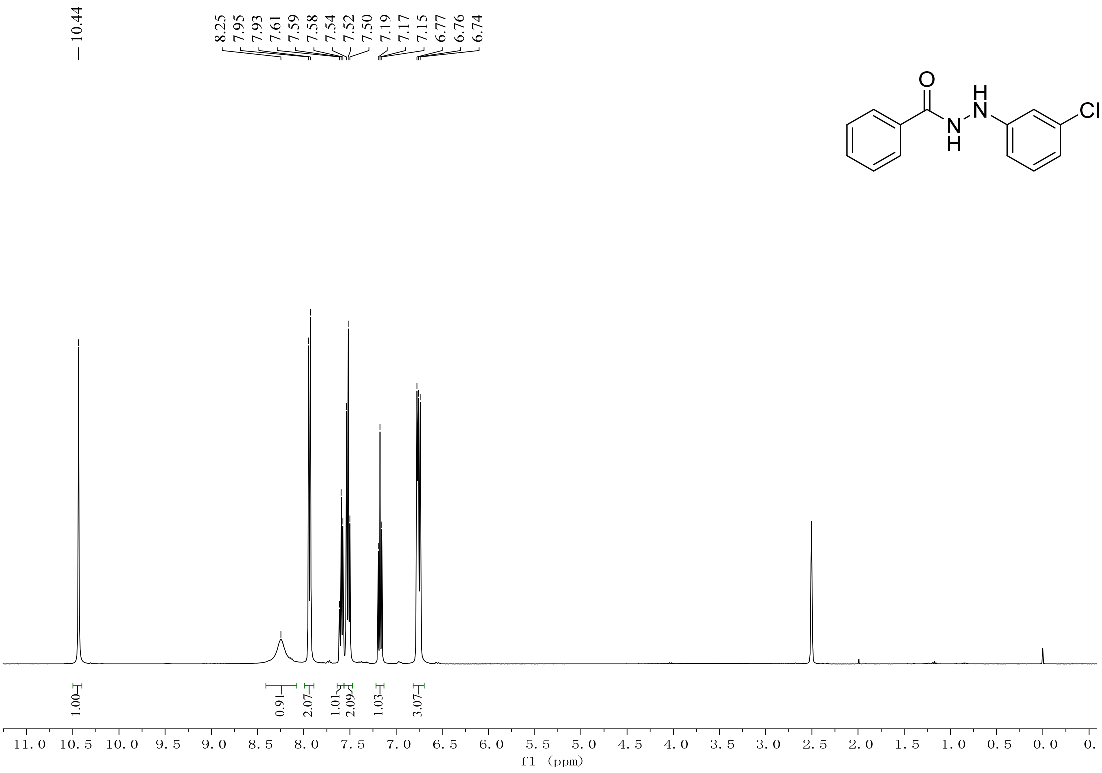

The ^{1}H NMR spectrum of **B**3.

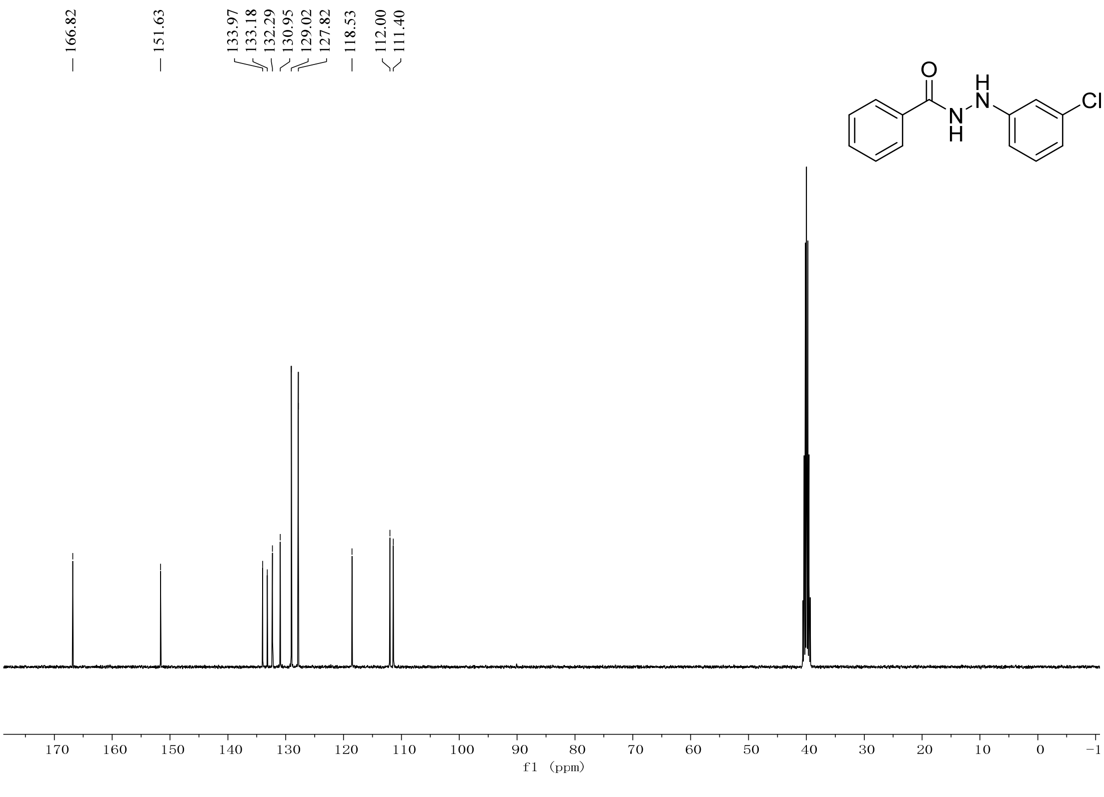

The ^{13}C NMR spectrum of **B**3.

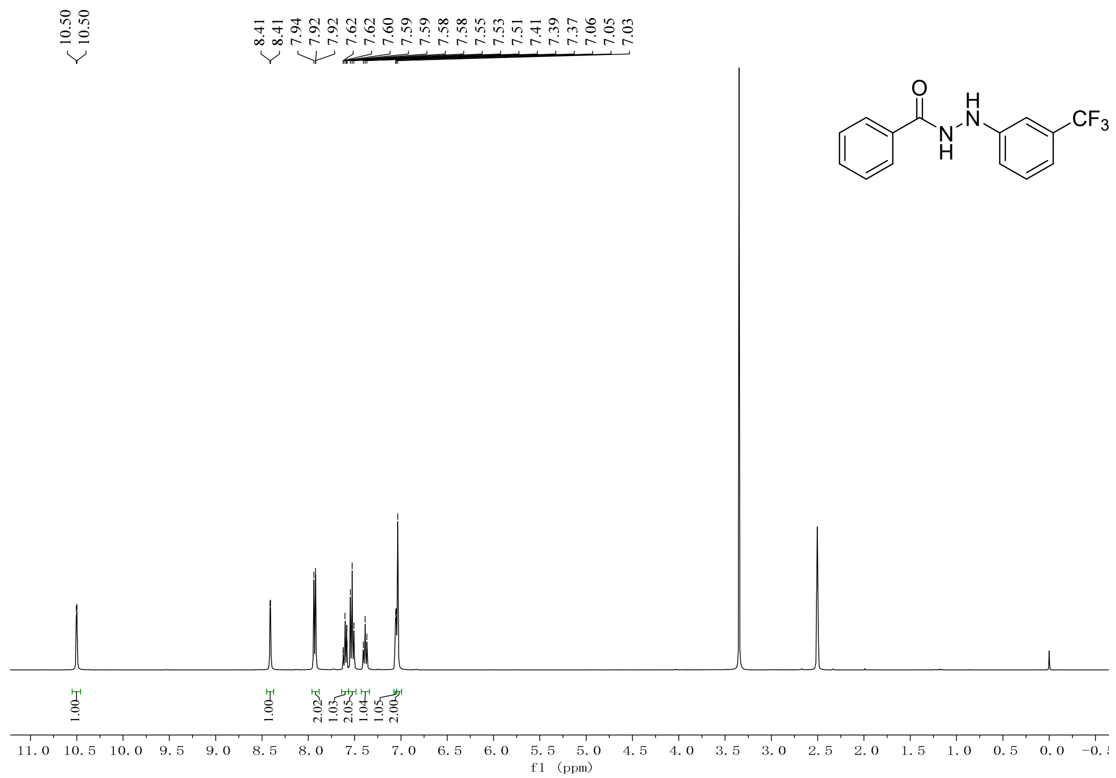

The ^{1}H NMR spectrum of **B4**.

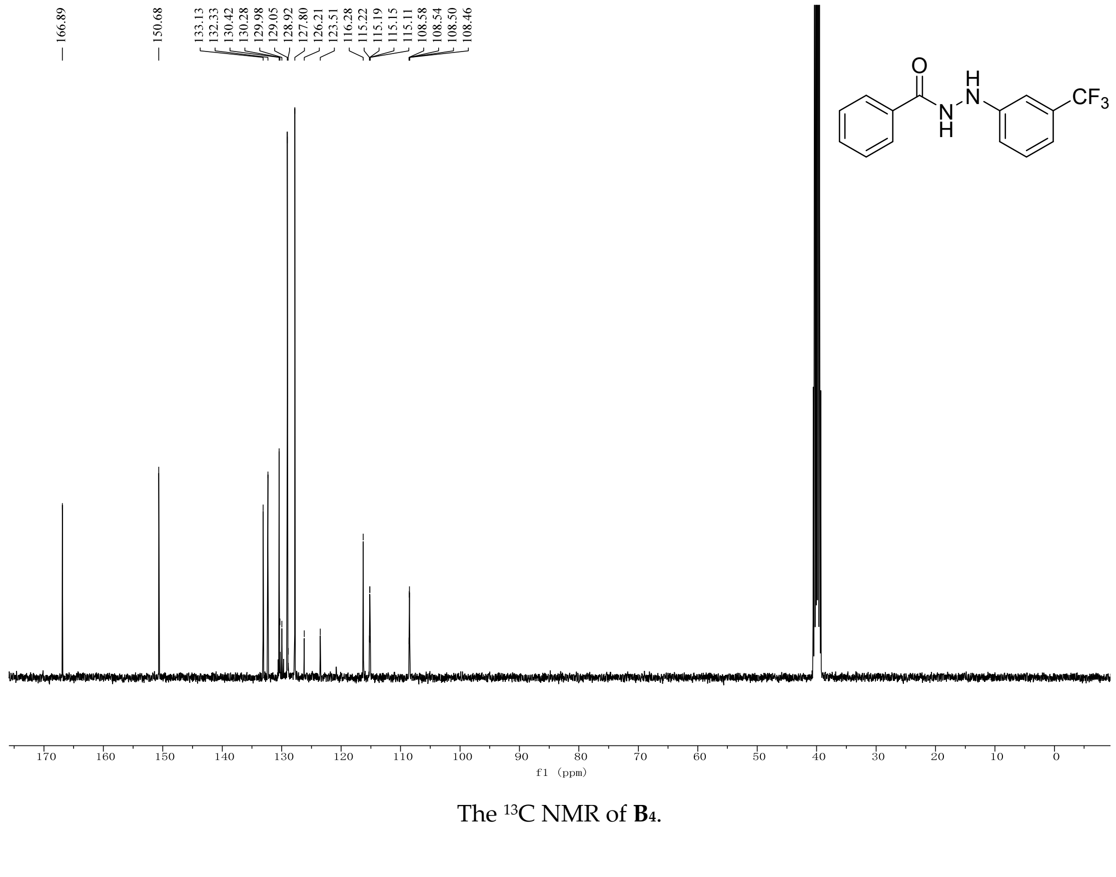

The ^{13}C NMR of **B4**.

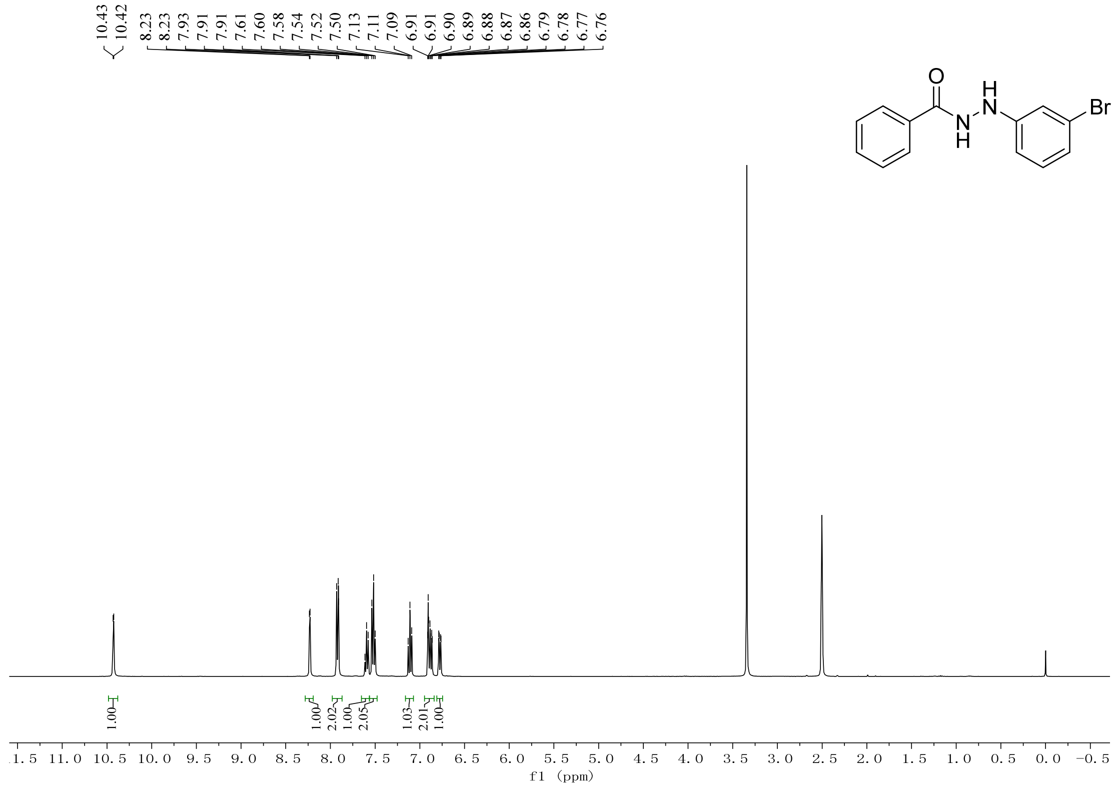

The ^{1}H NMR spectrum of **B**5.

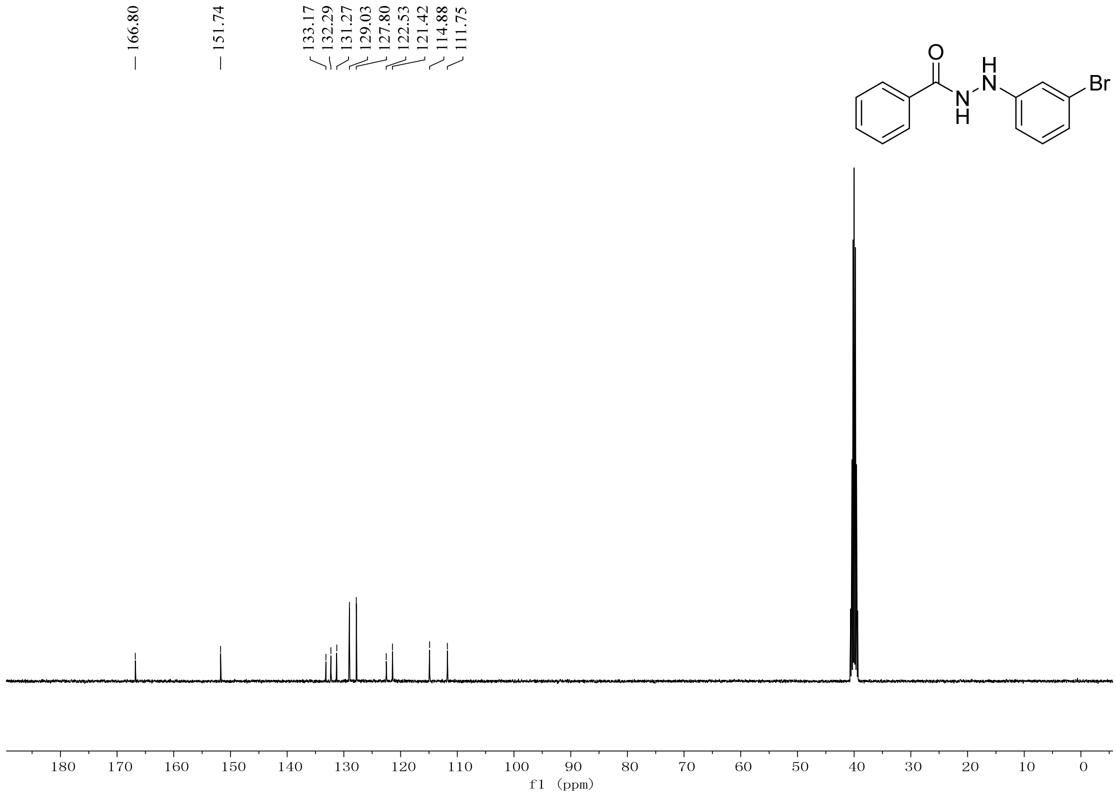

The ^{13}C NMR spectrum of **B**5.

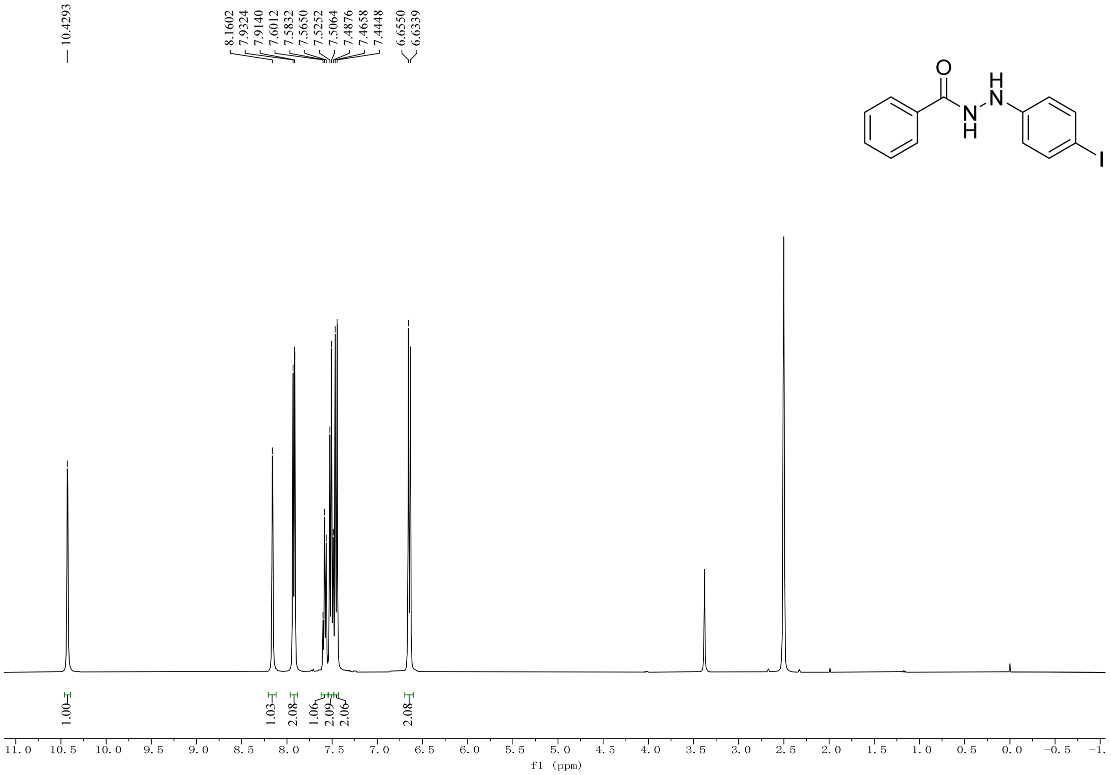

The ^{1}H NMR spectrum of **B6**.

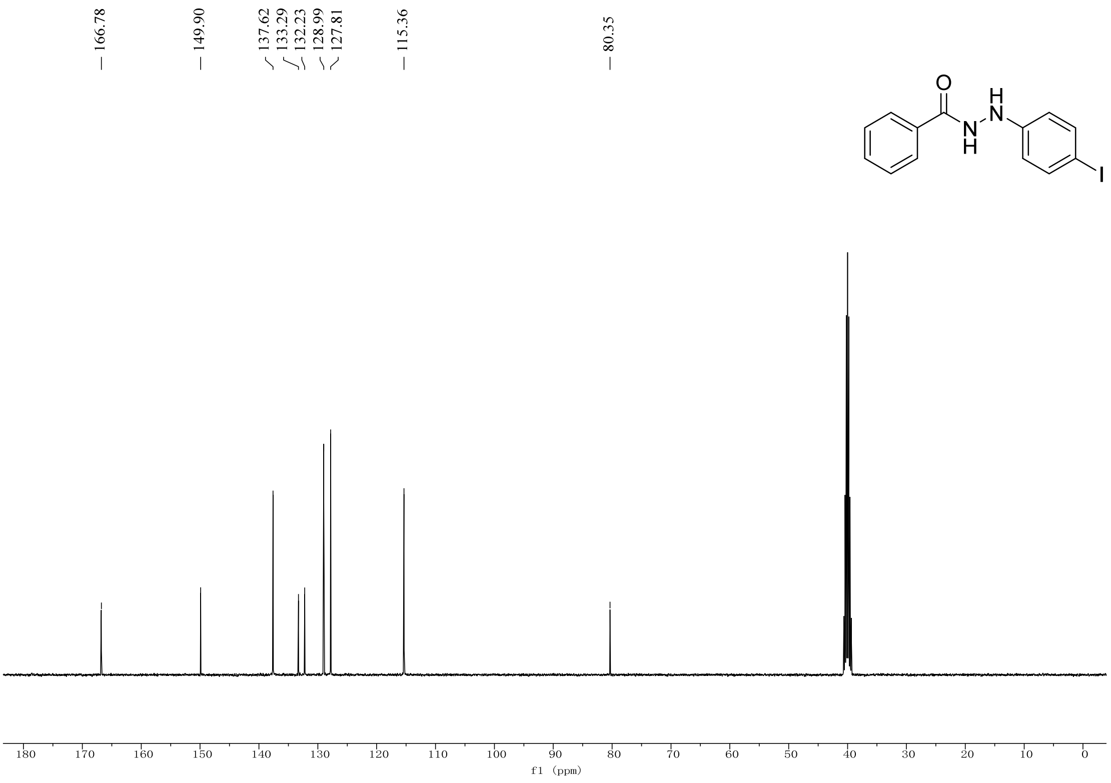

The ^{13}C NMR spectrum of **B6**.

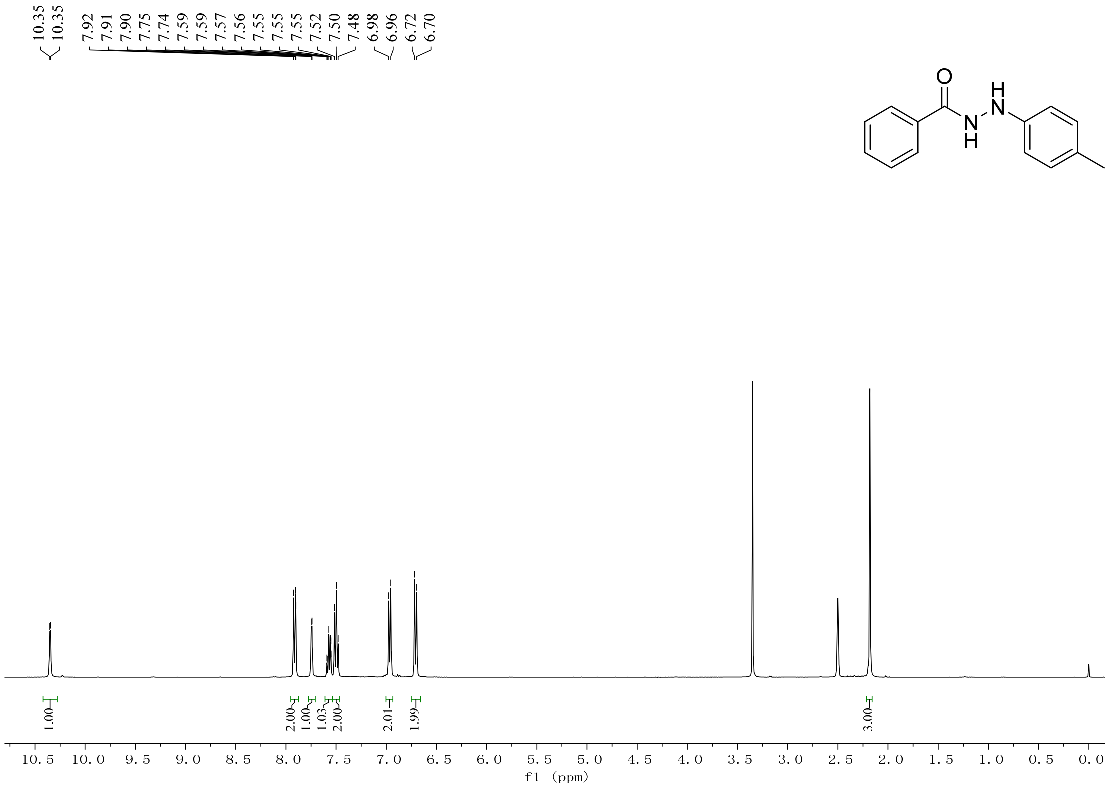

The ^{1}H NMR spectrum of **B**7.

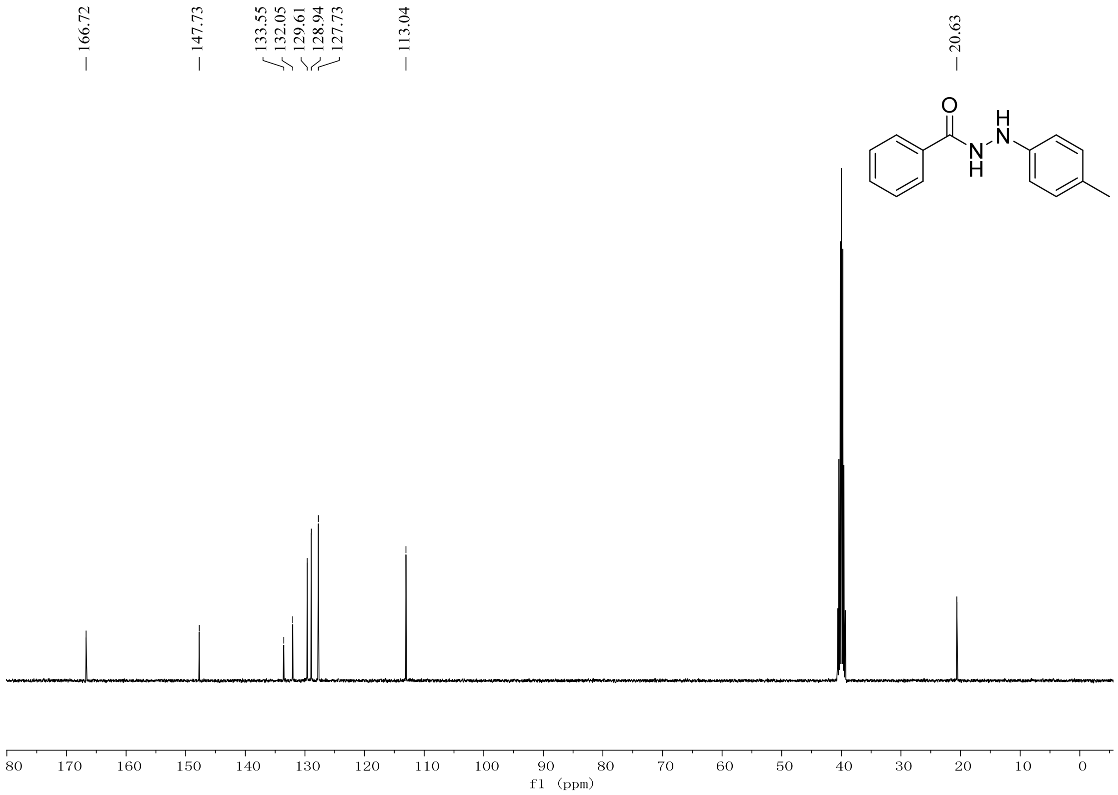

The ^{13}C NMR spectrum of **B**7.

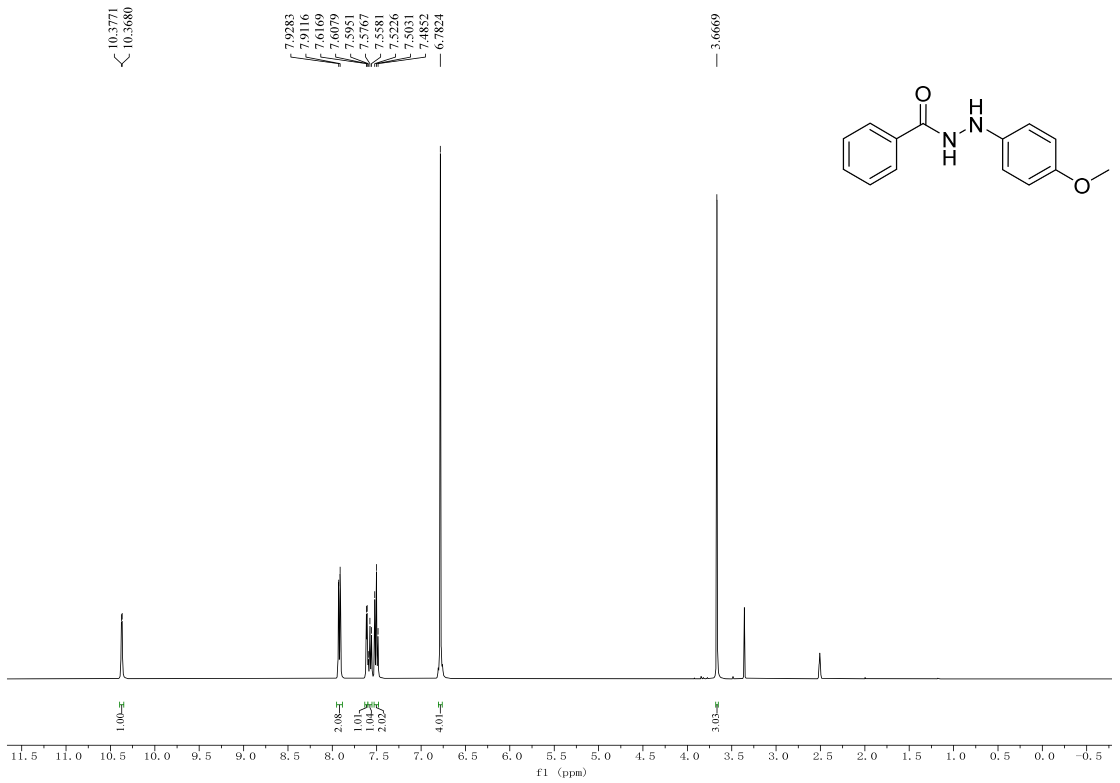

The ^{1}H NMR spectrum of **B8**.

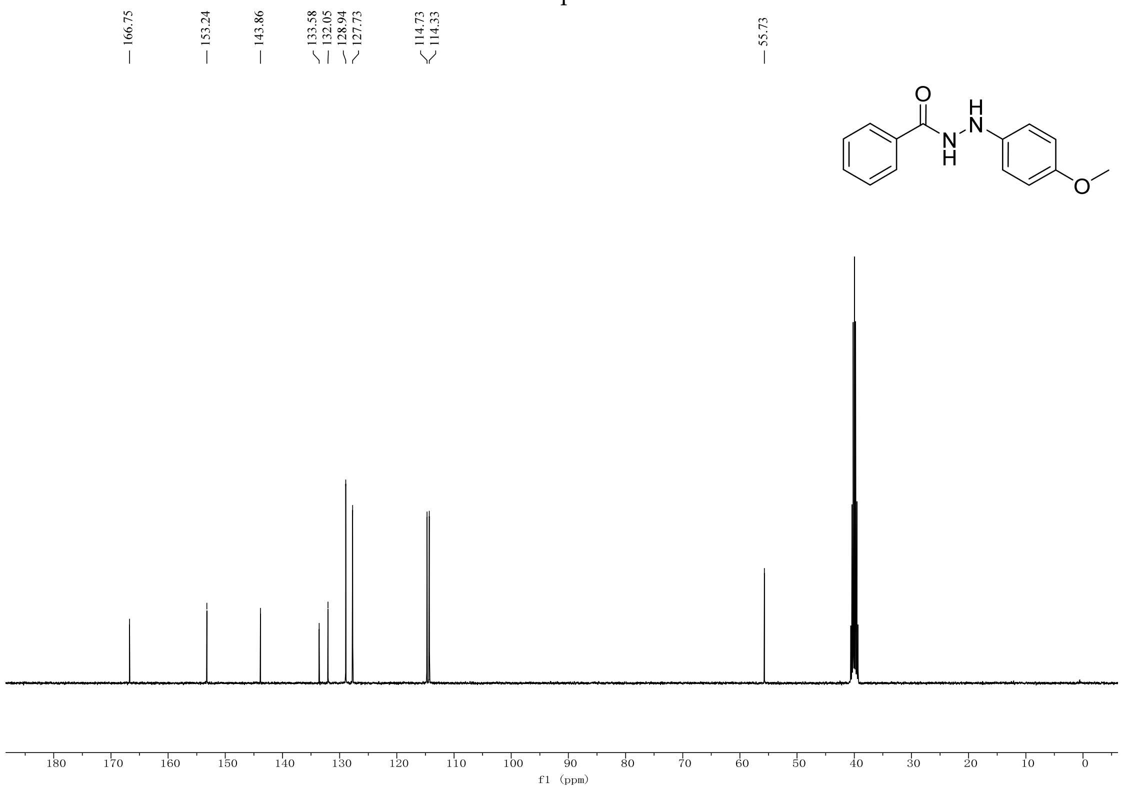

The ^{13}C NMR spectrum of **B8**.

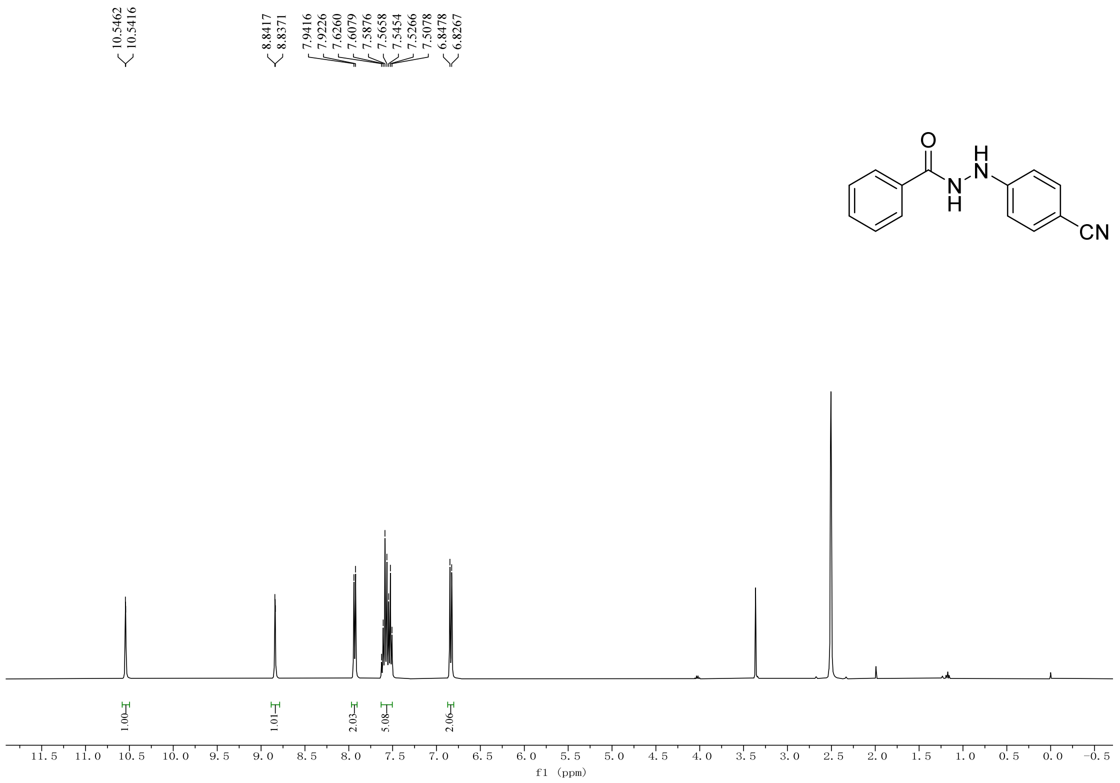

The ^{1}H NMR spectrum of **B9**.

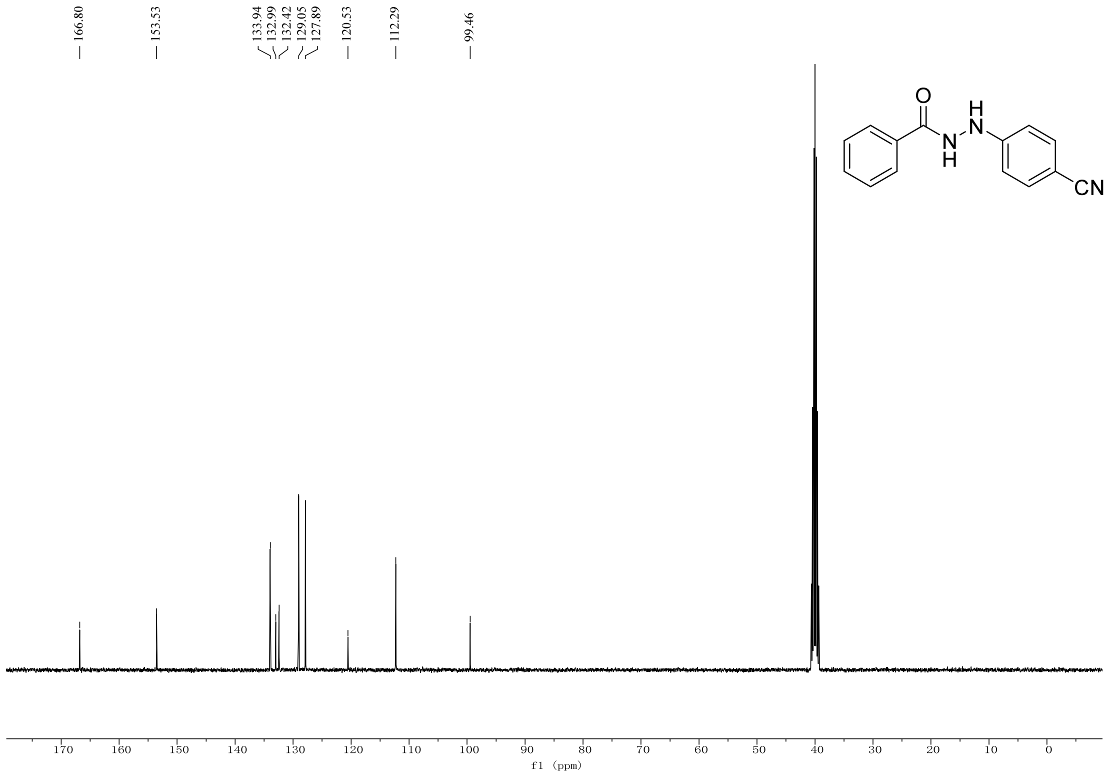

The ^{13}C NMR spectrum of **B9**.

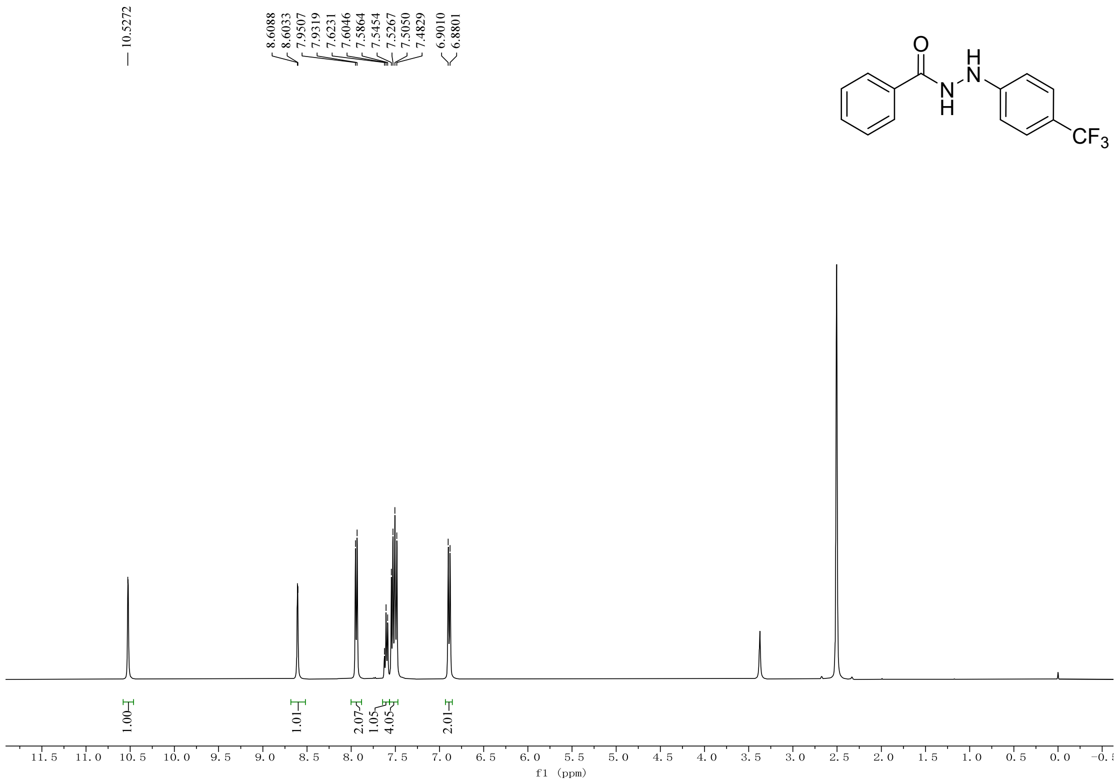

The ^{1}H NMR spectrum of **B10**.

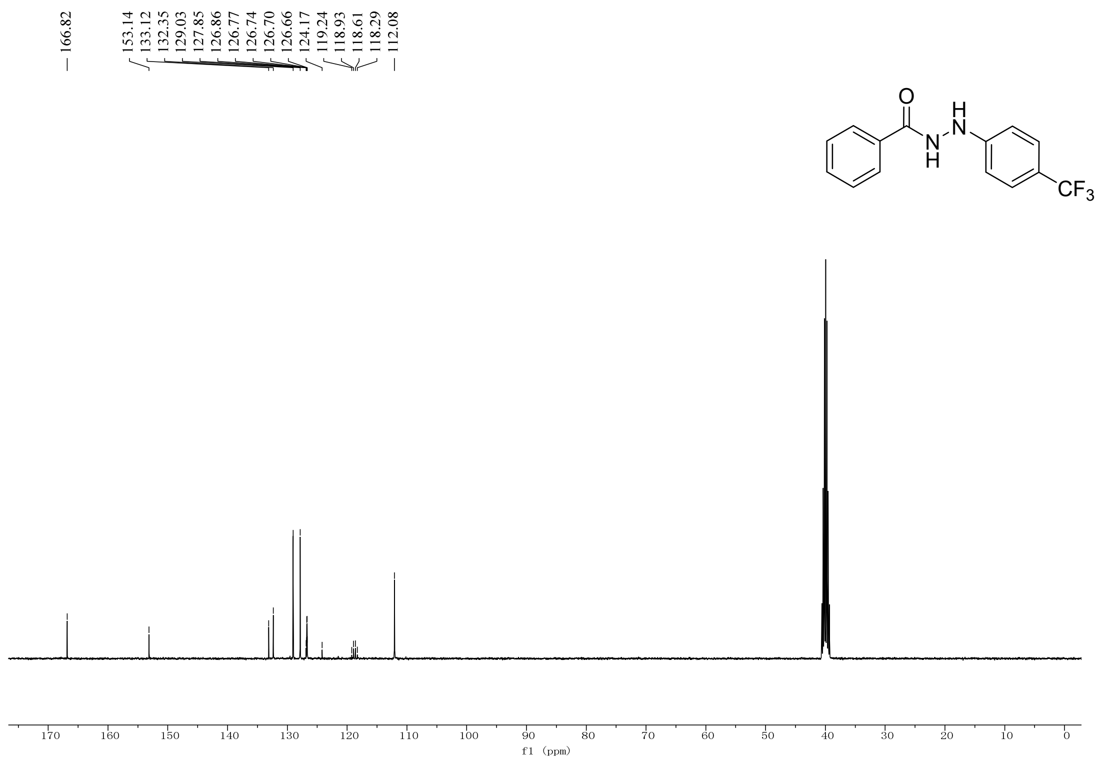

The ^{13}C NMR spectrum of **B10**.

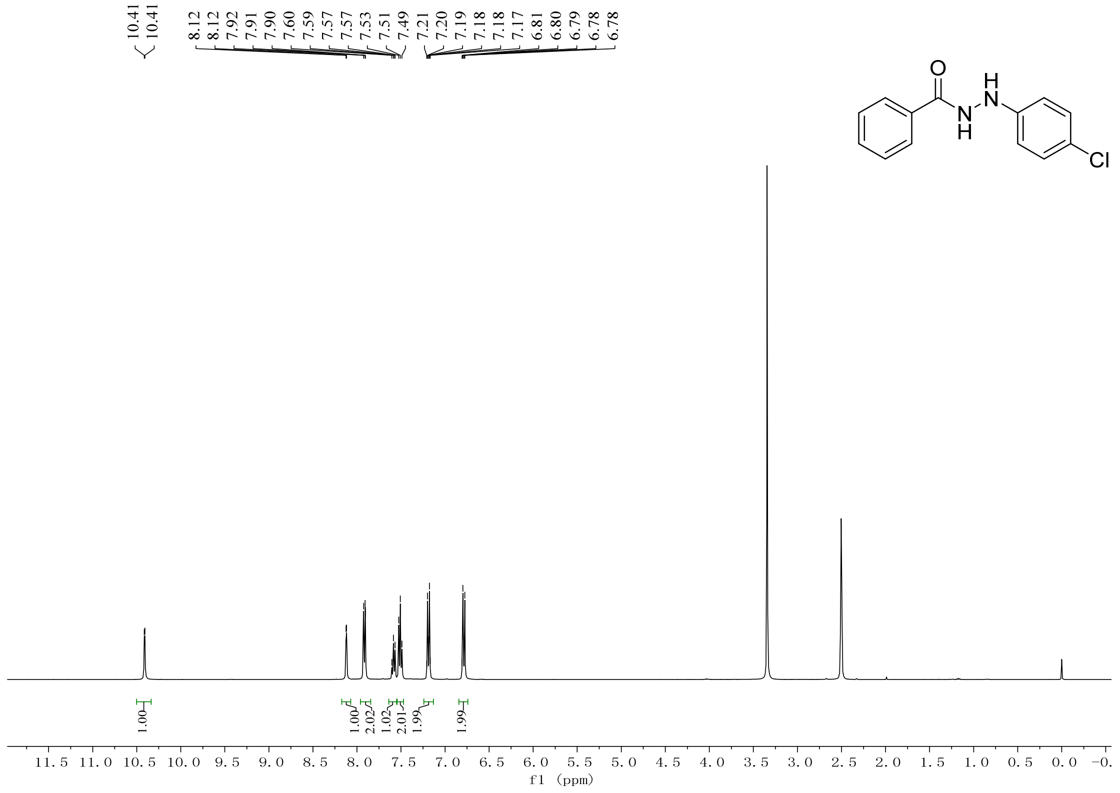

The ^{1}H NMR spectrum of **B11**.

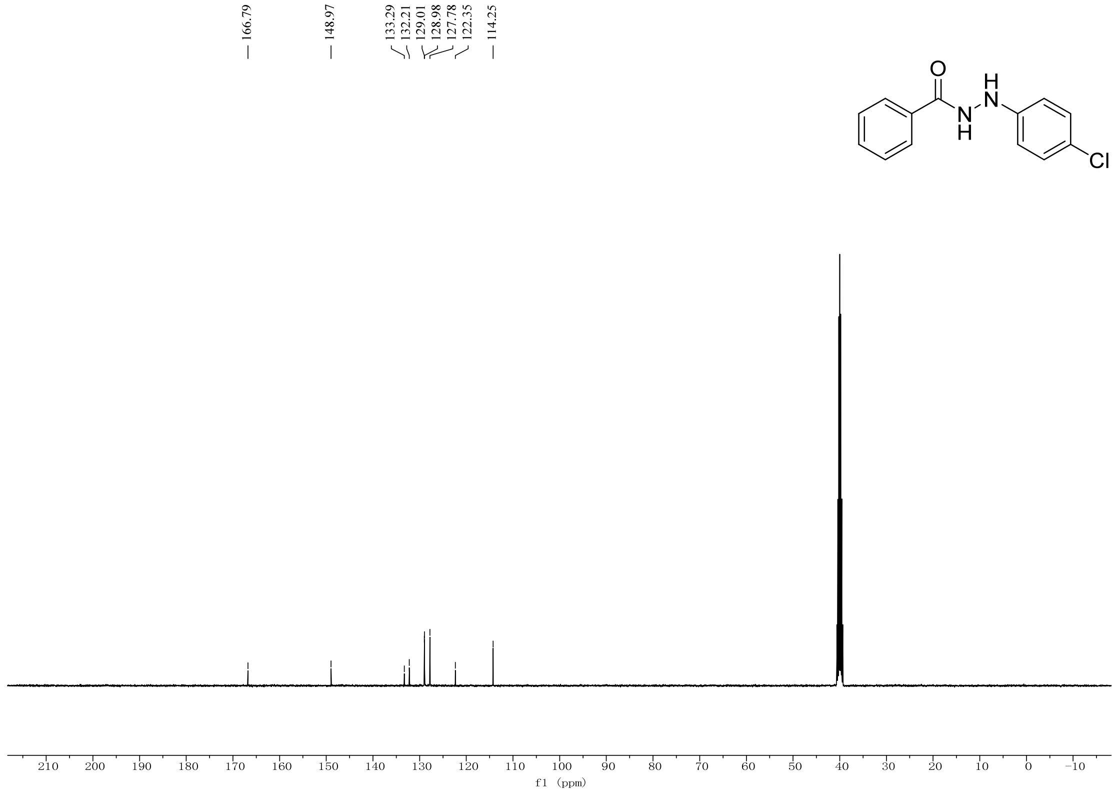

The ^{13}C NMR spectrum of **B11**.

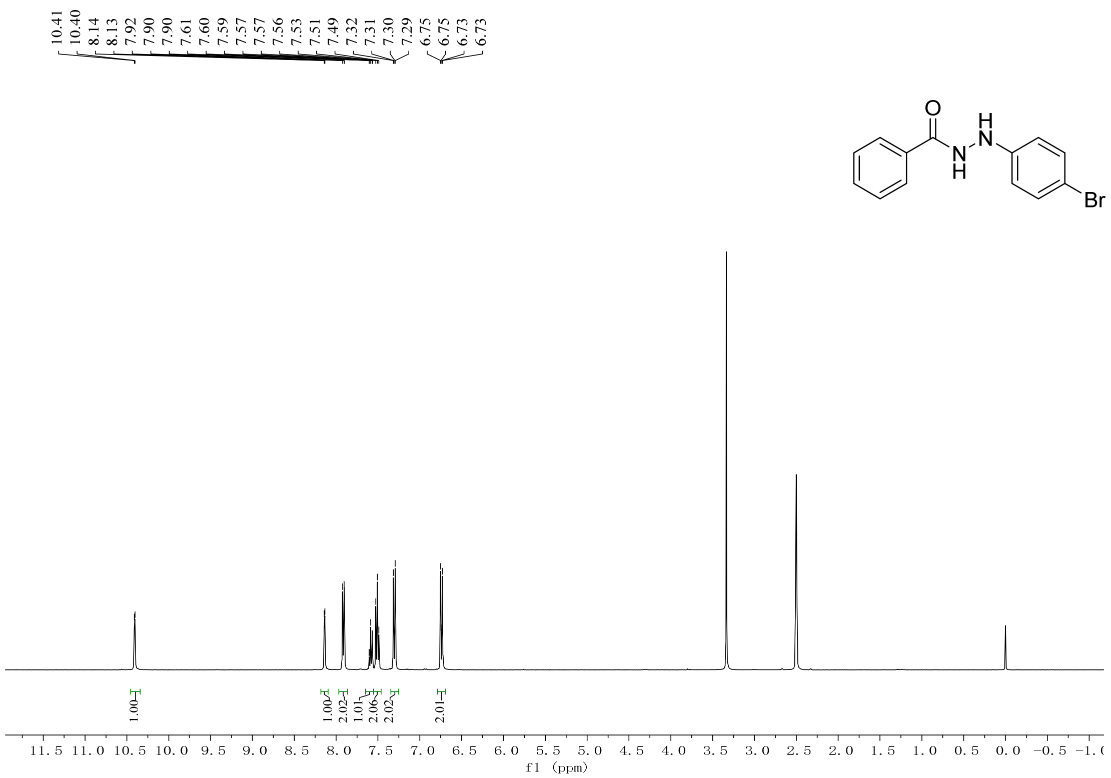

The ^{1}H NMR spectrum of **B12**.

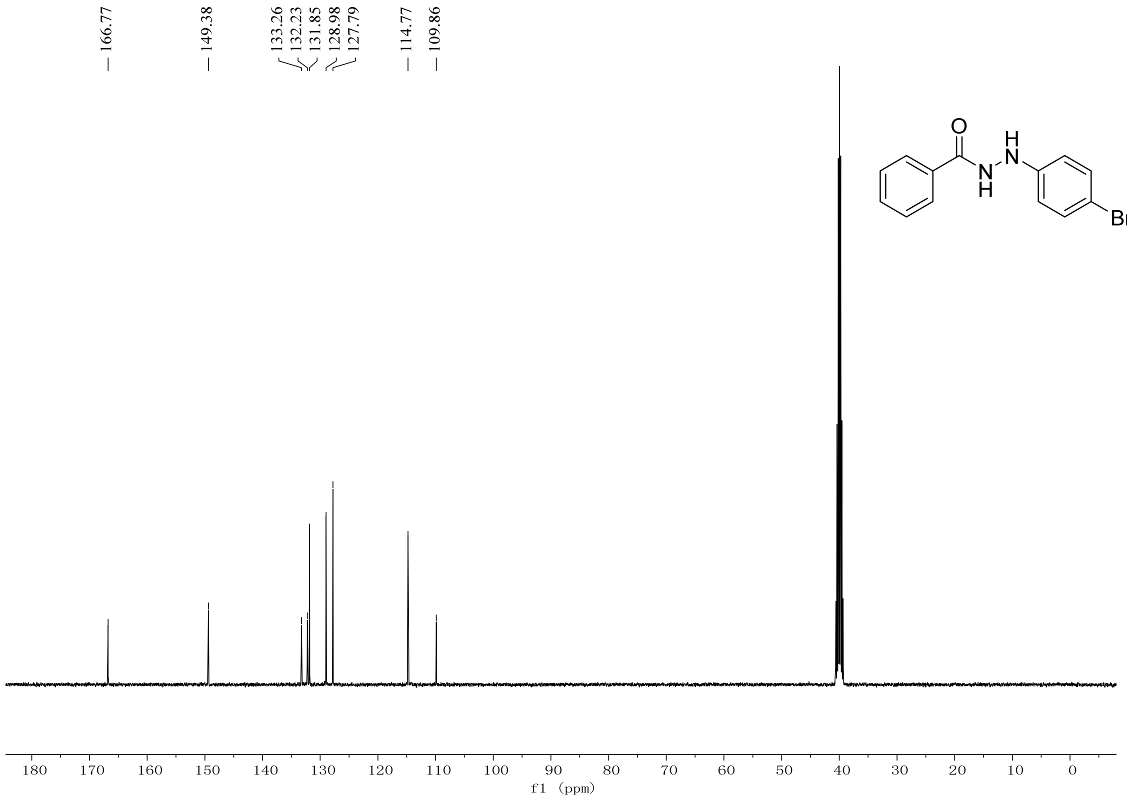

The ^{13}C NMR spectrum of **B12**.

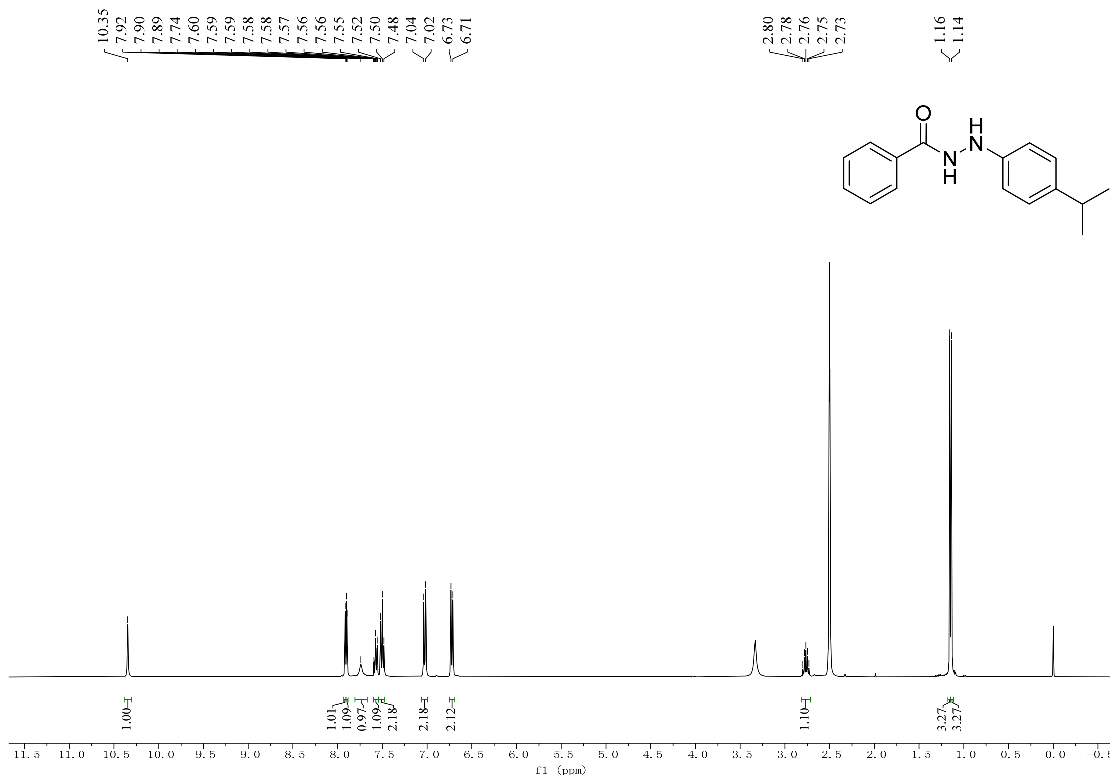

The ^{1}H NMR spectrum of **B13**.

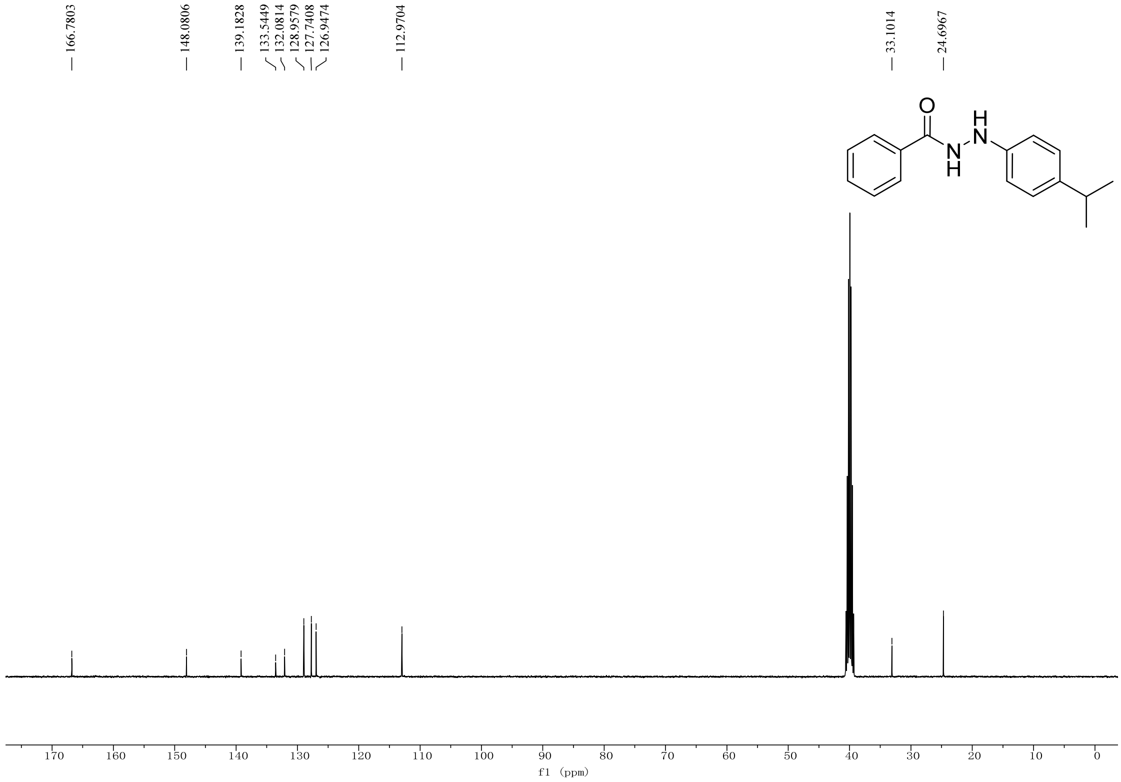

The ^{13}C NMR spectrum of **B13**.

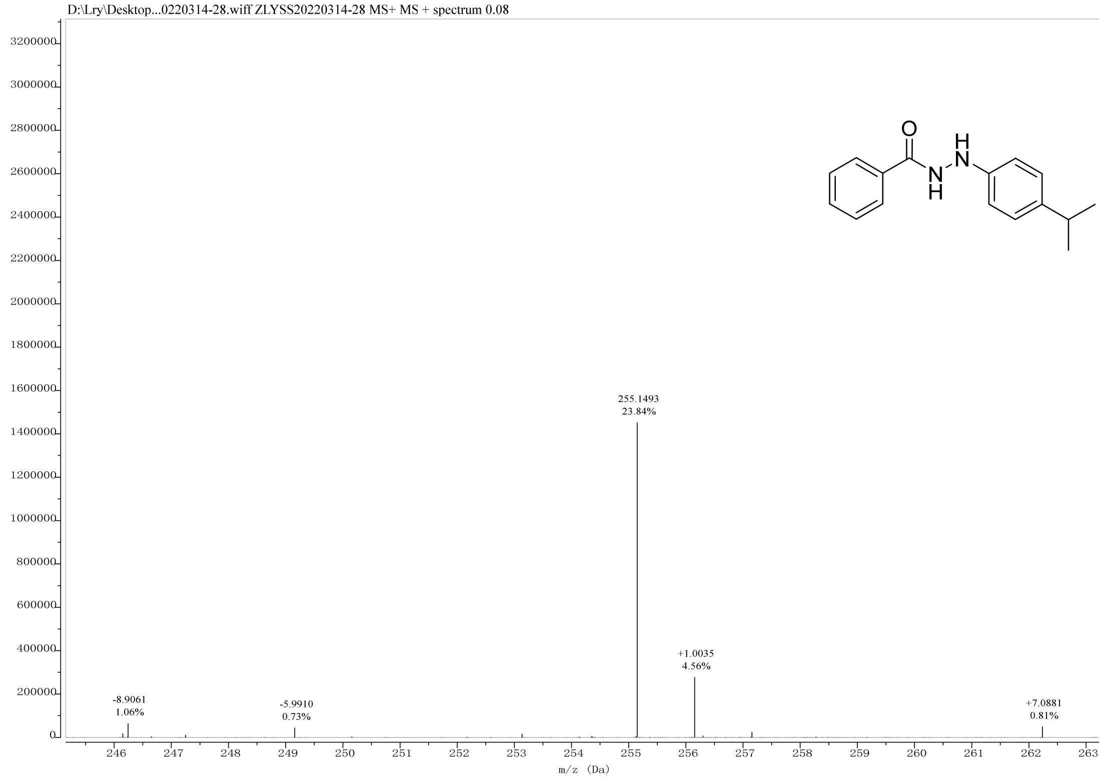

The HRMS spectrum of **B13**.

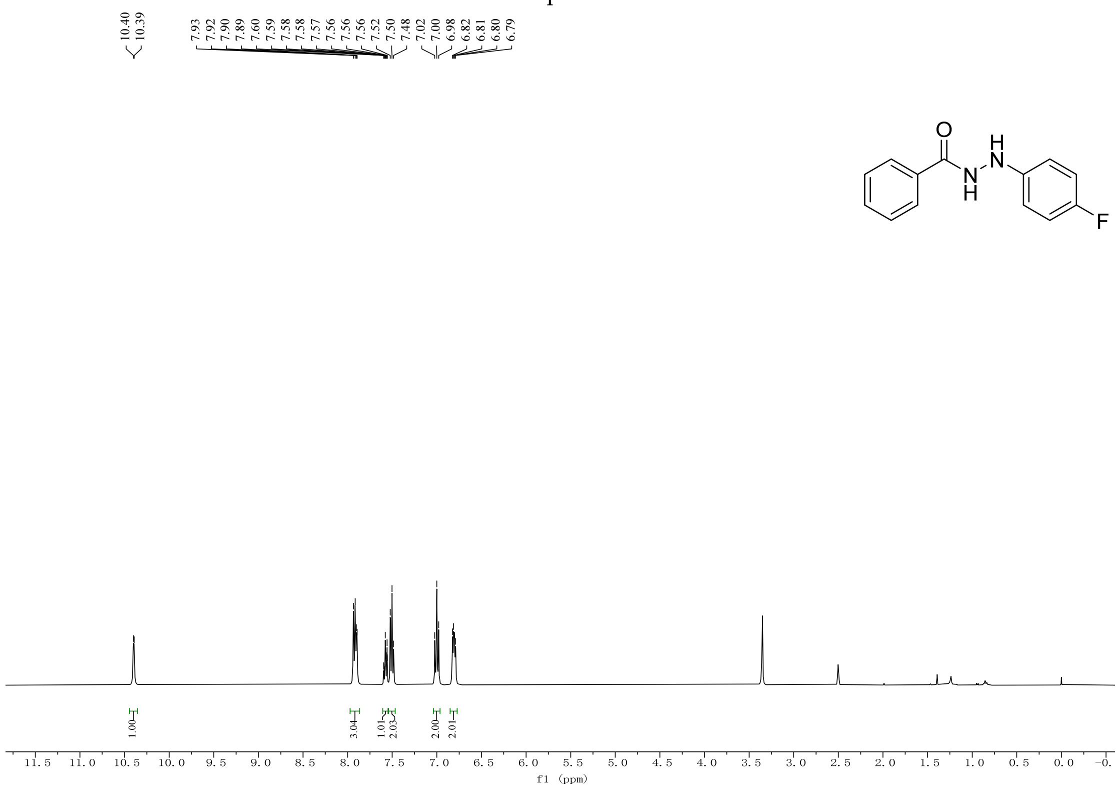

The ^{1}H NMR spectrum of **B14**.

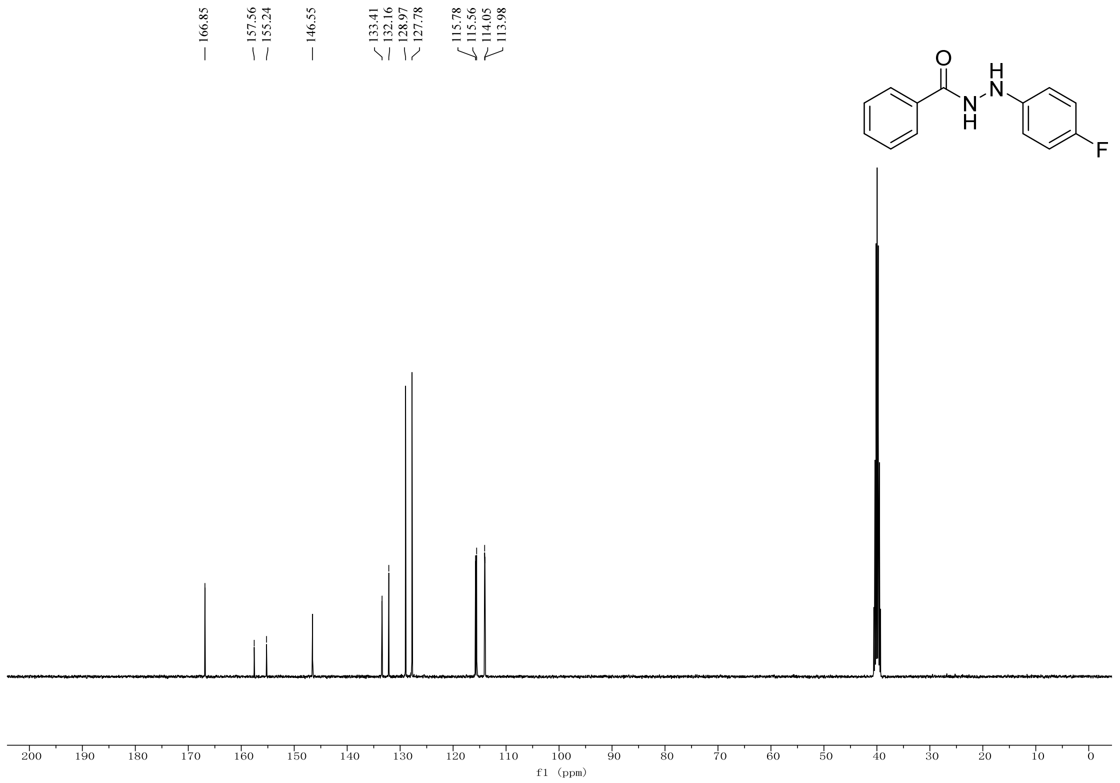

The ^{13}C NMR spectrum of **B14**.

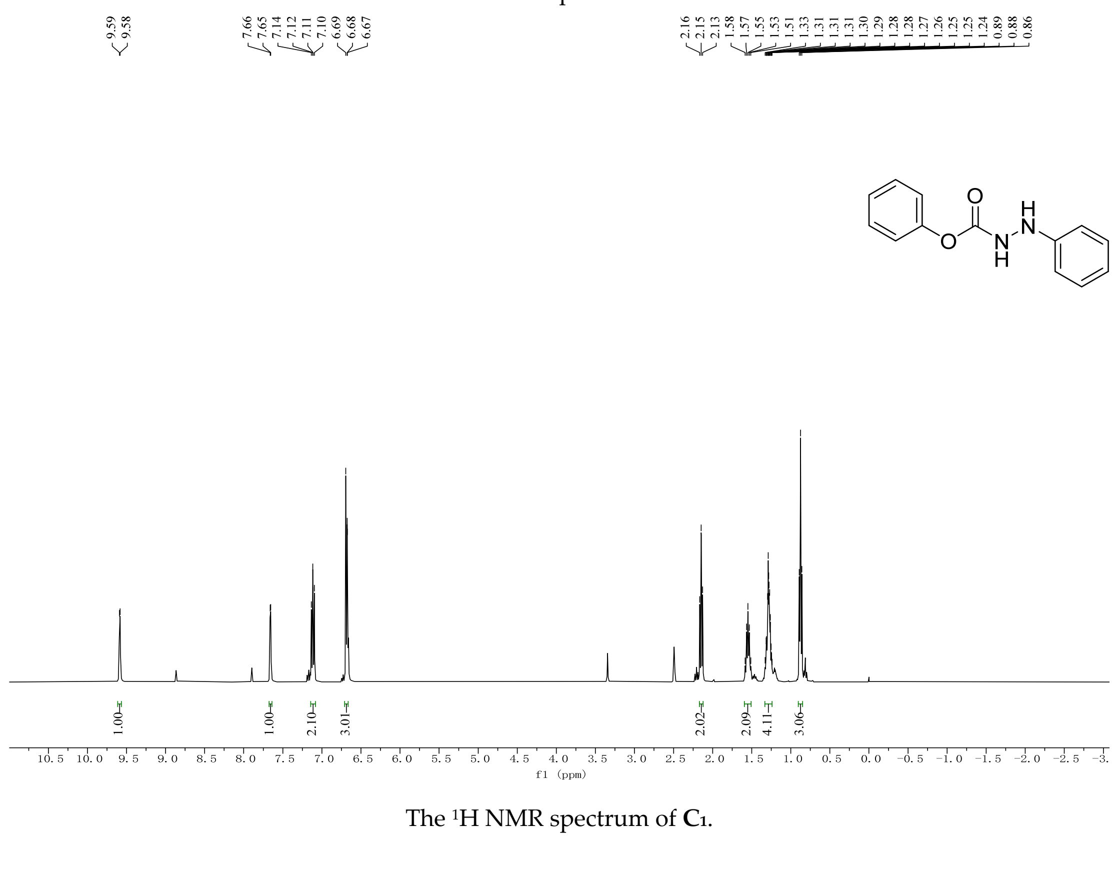

The ^{1}H NMR spectrum of **C1**.

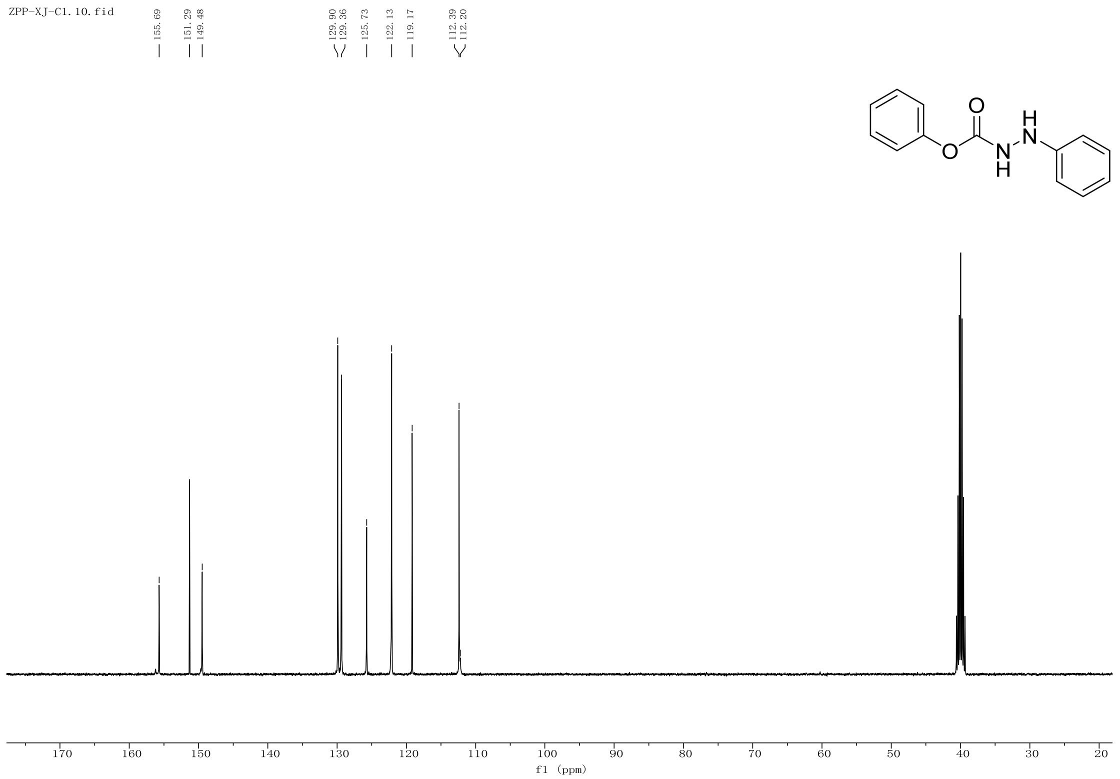

The ^{13}C NMR spectrum of **C1**.

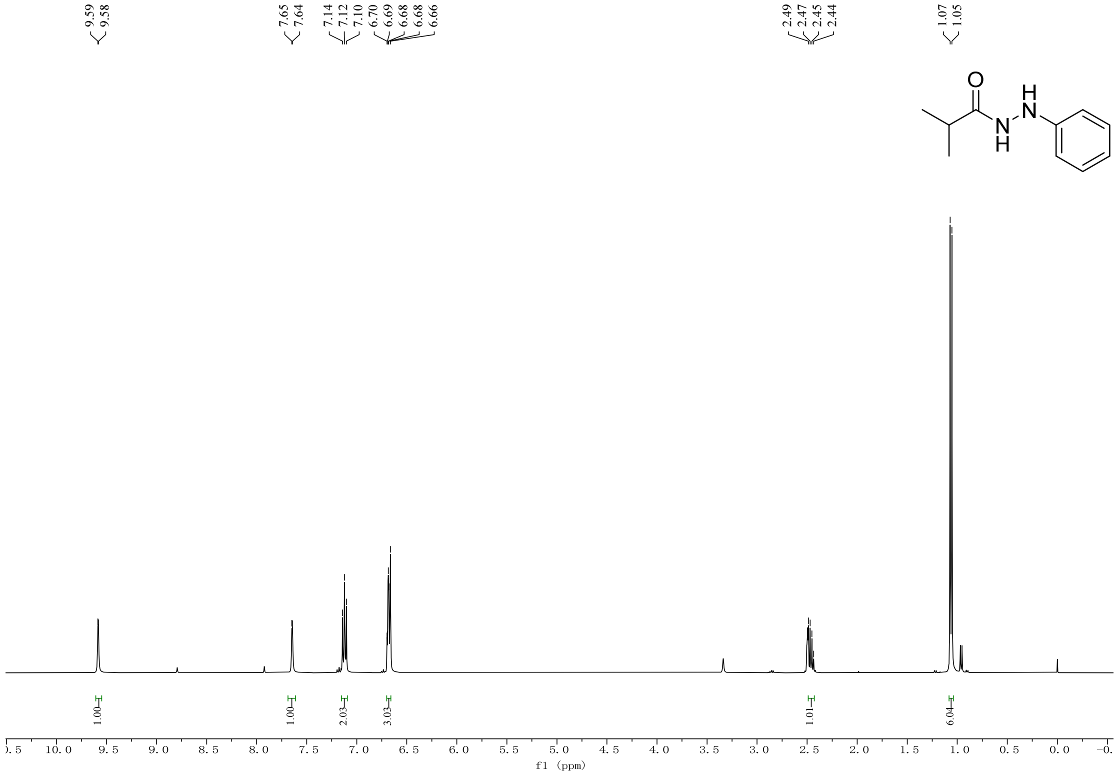

The ^{1}H NMR spectrum of **C2**.

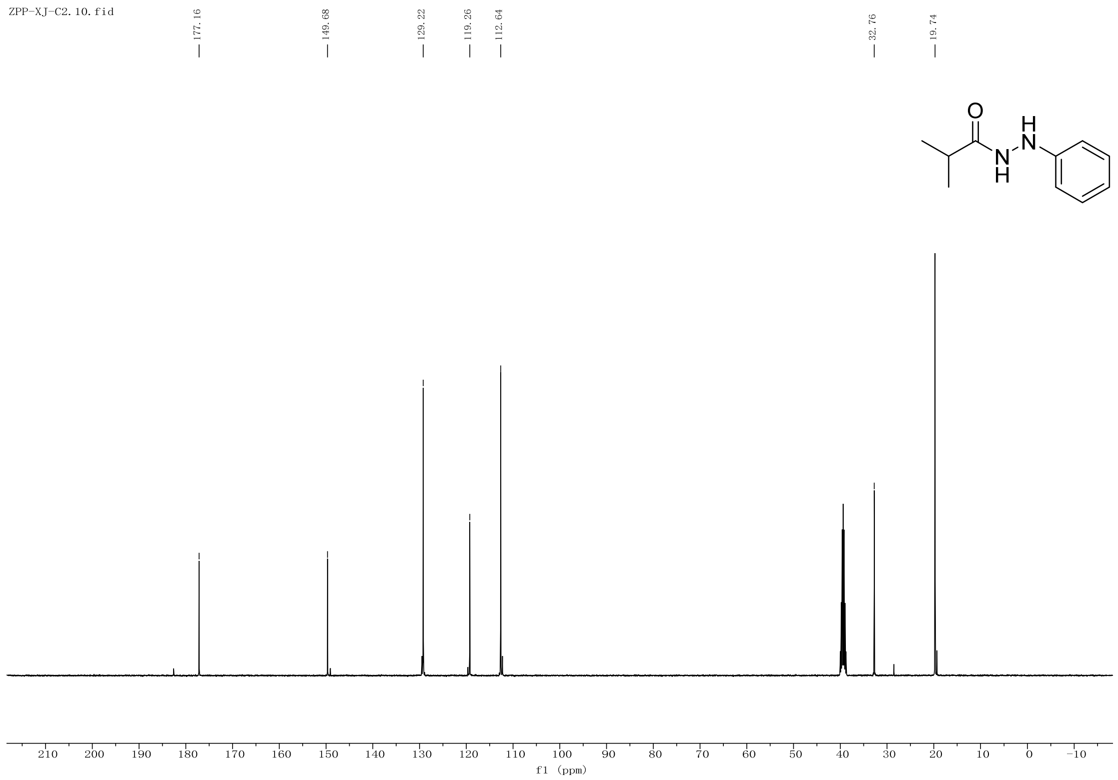

The ^{13}C NMR spectrum of **C2**.

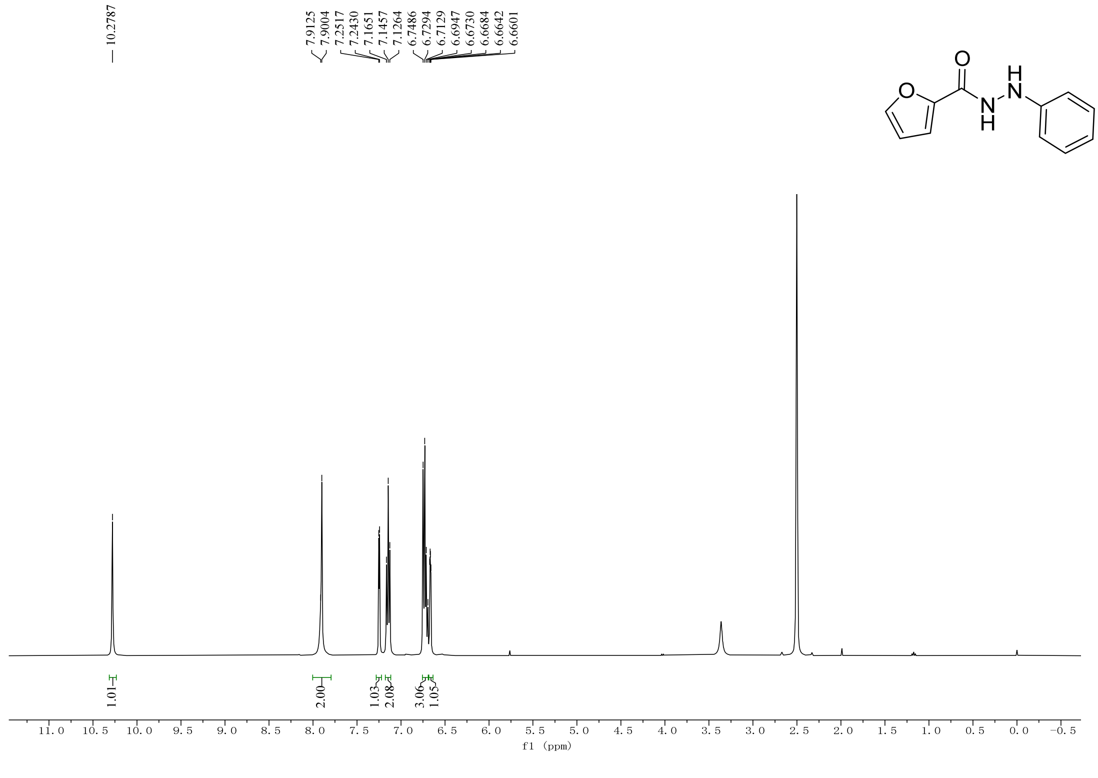

The ^{1}H NMR spectrum of **C3**.

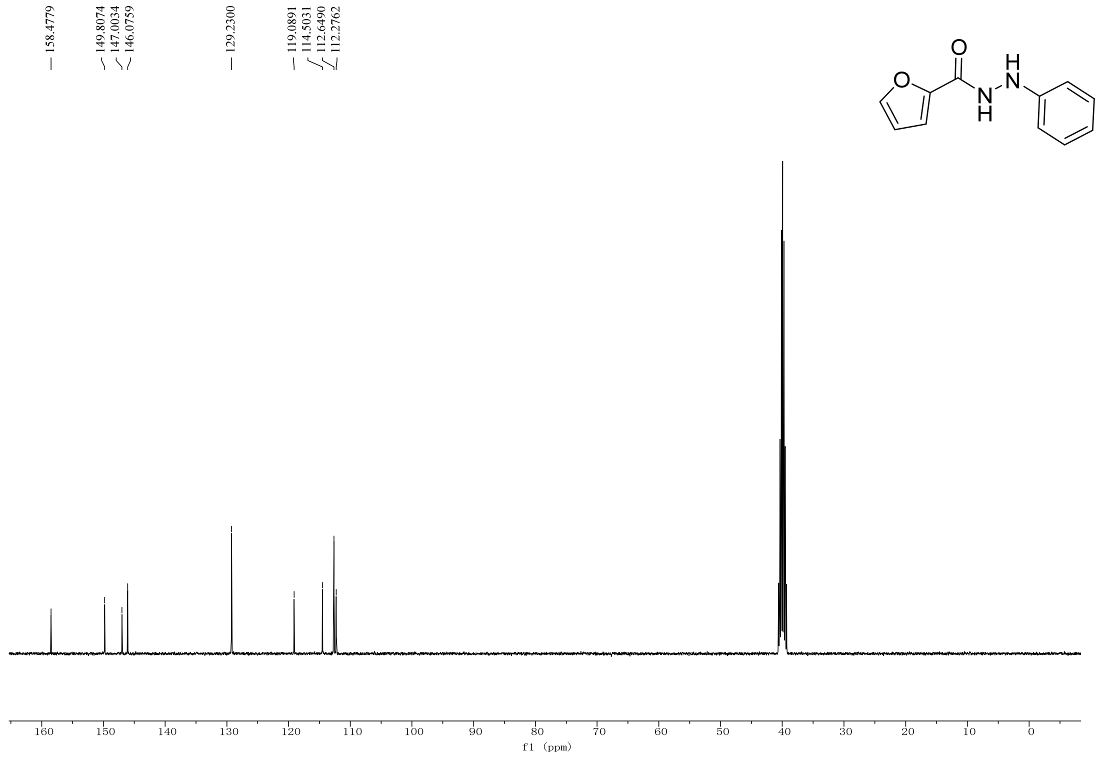

The ^{13}C NMR spectrum of **C3**.

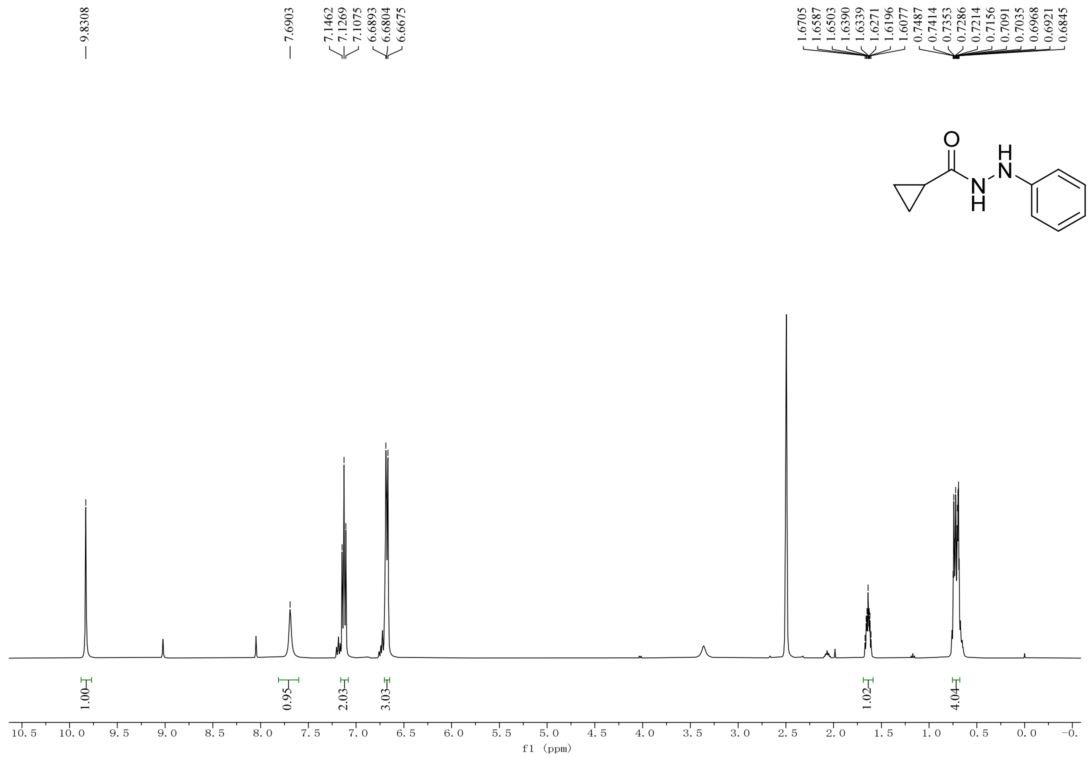

The ^{1}H NMR spectrum of **C4**.

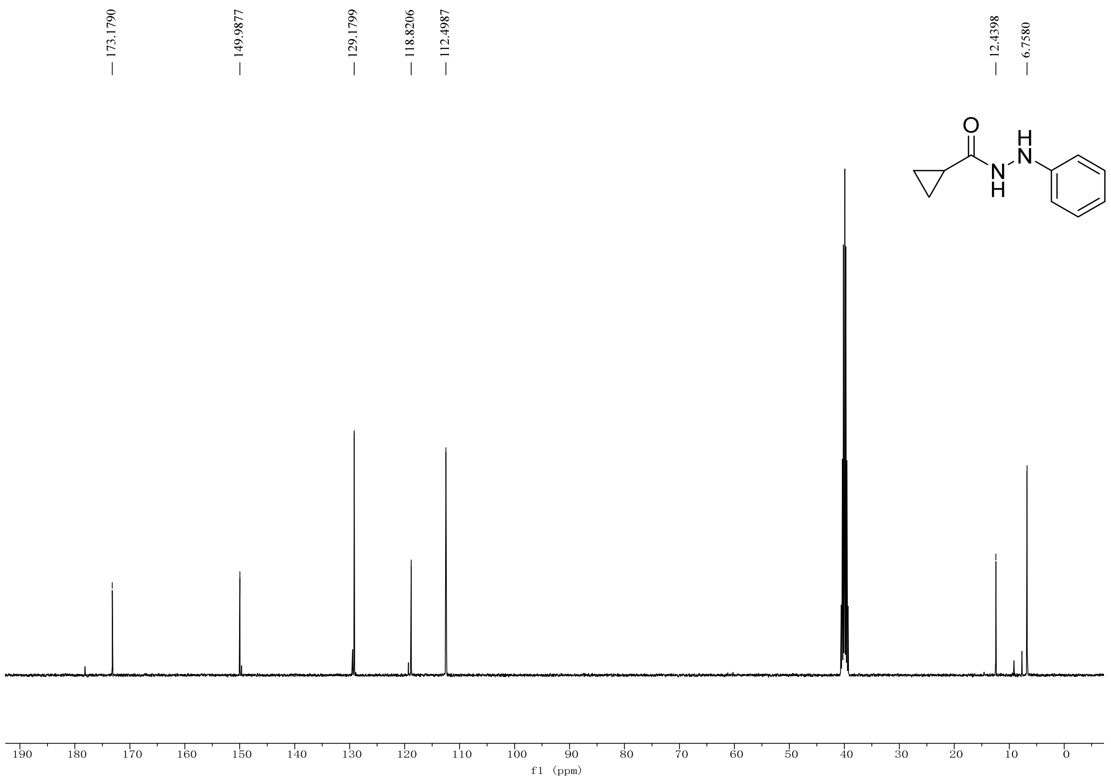

The ^{13}C NMR spectrum of **C4**.

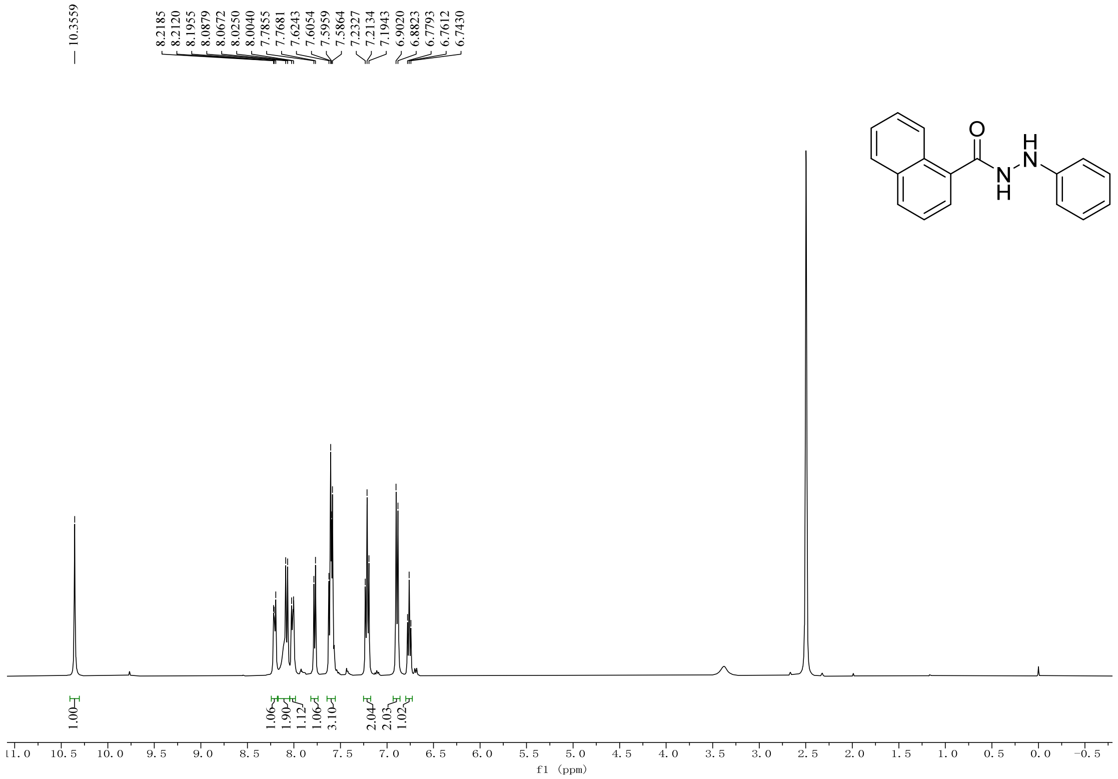

The ^{1}H NMR spectrum of **C5**.

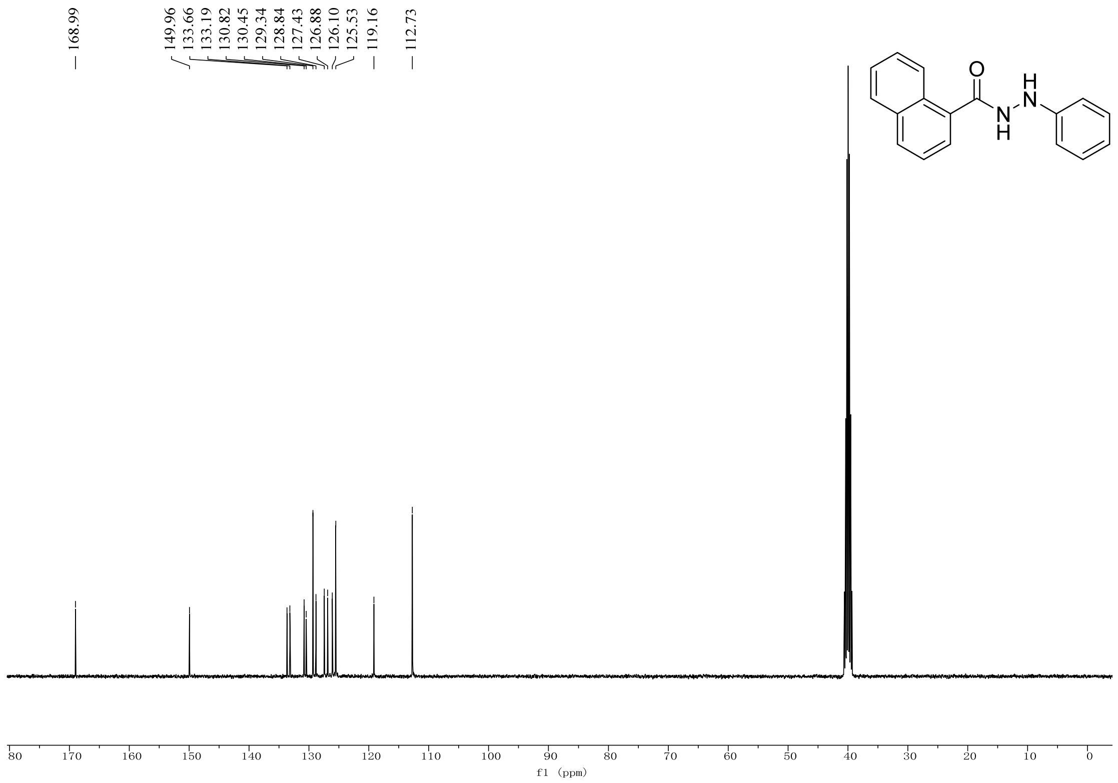

The ^{13}C NMR spectrum of **C5**.

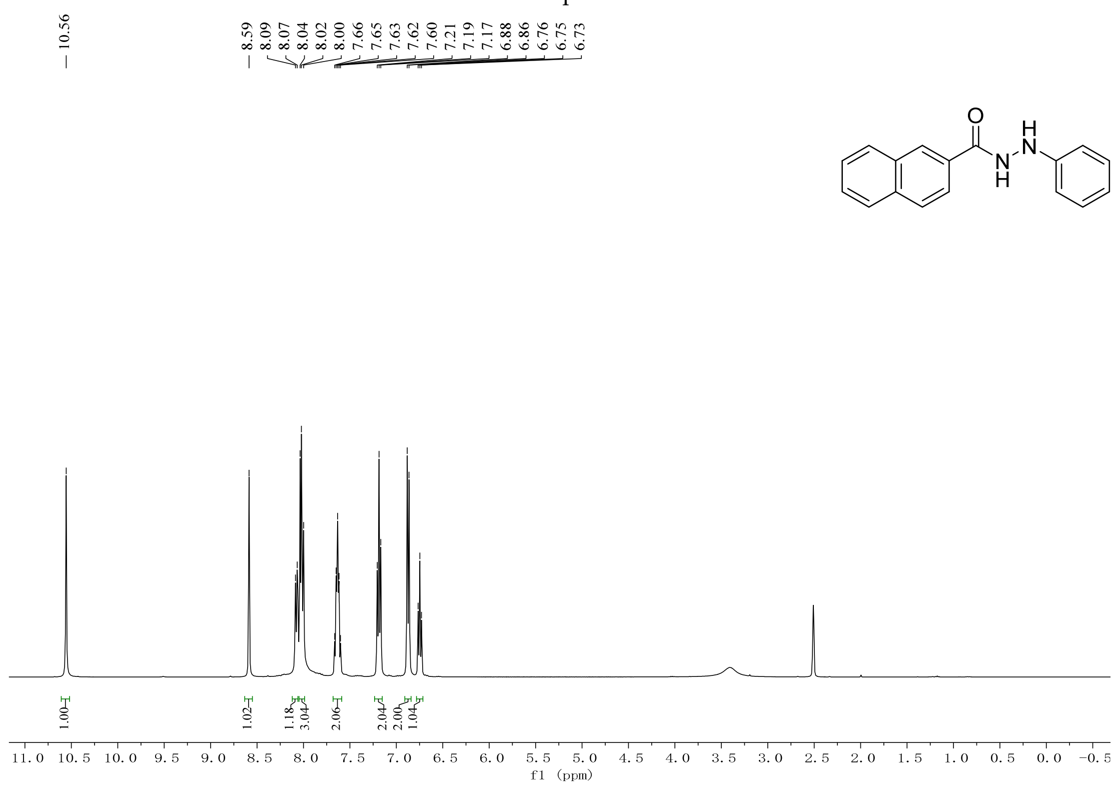

The ^{1}H NMR spectrum of **C6**.

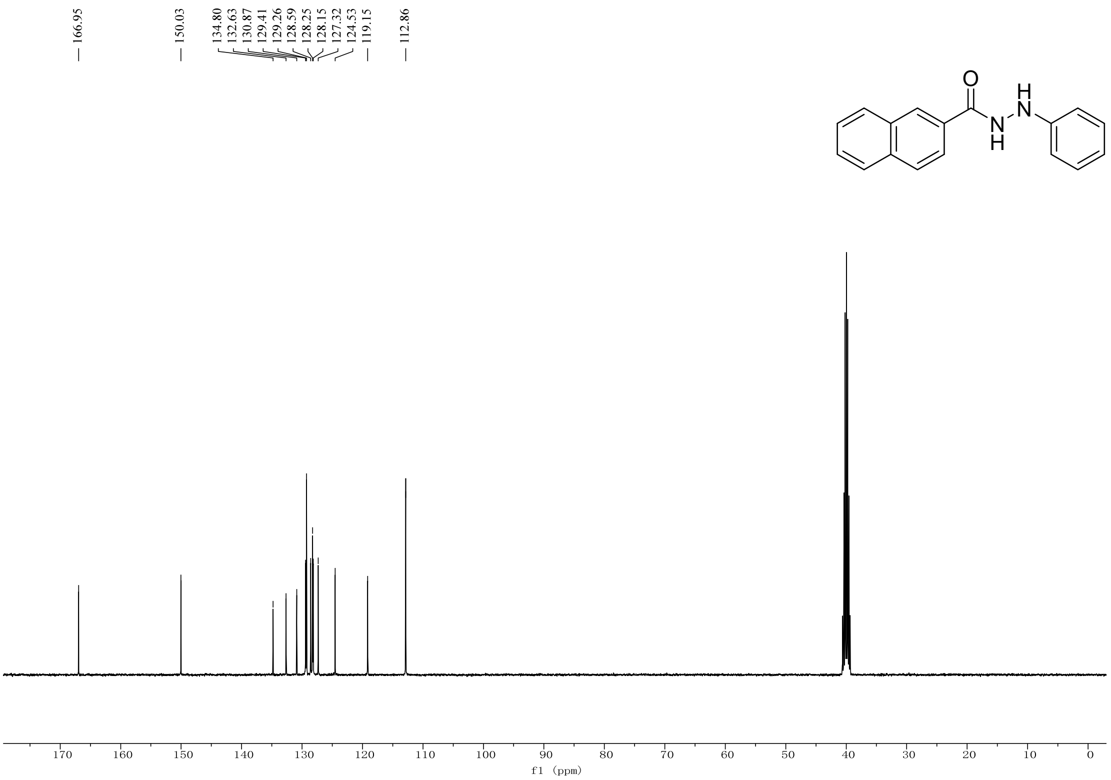

The ^{13}C NMR spectrum of **C6**.

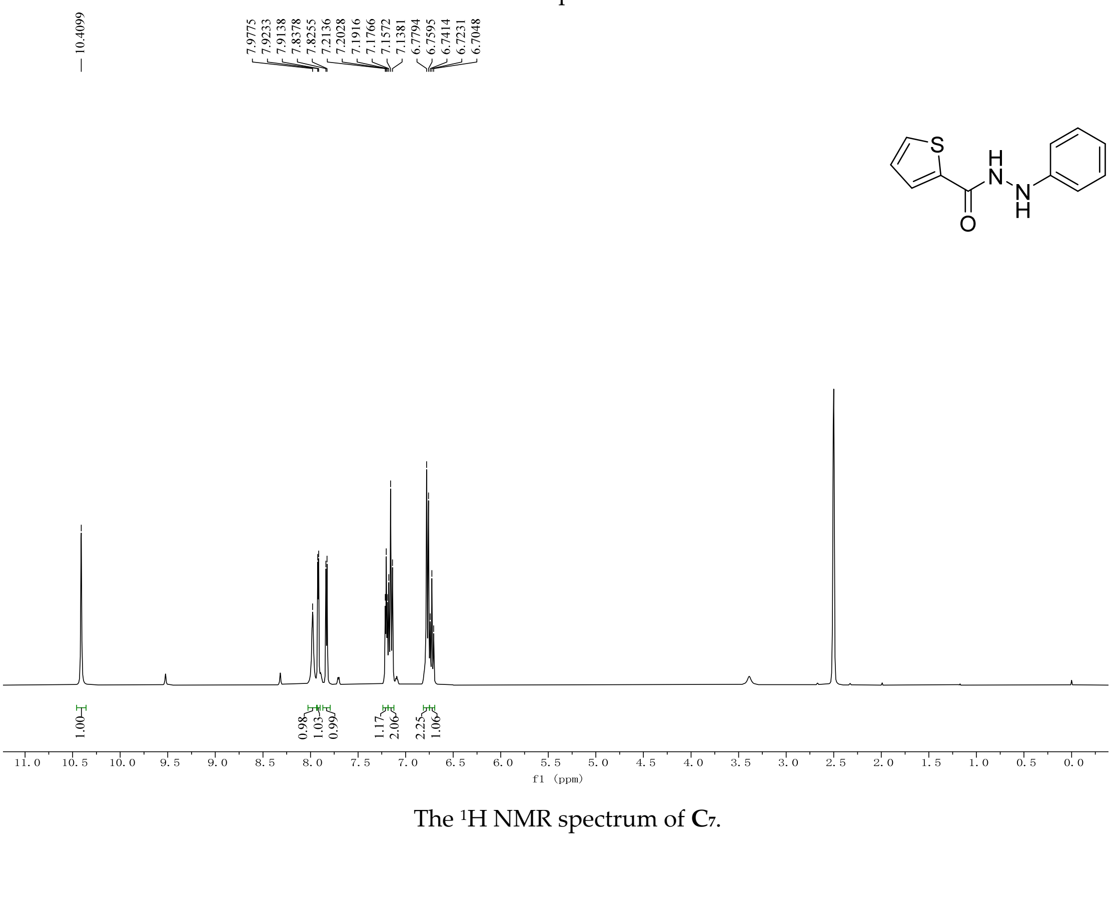

The ^{1}H NMR spectrum of **C7**.

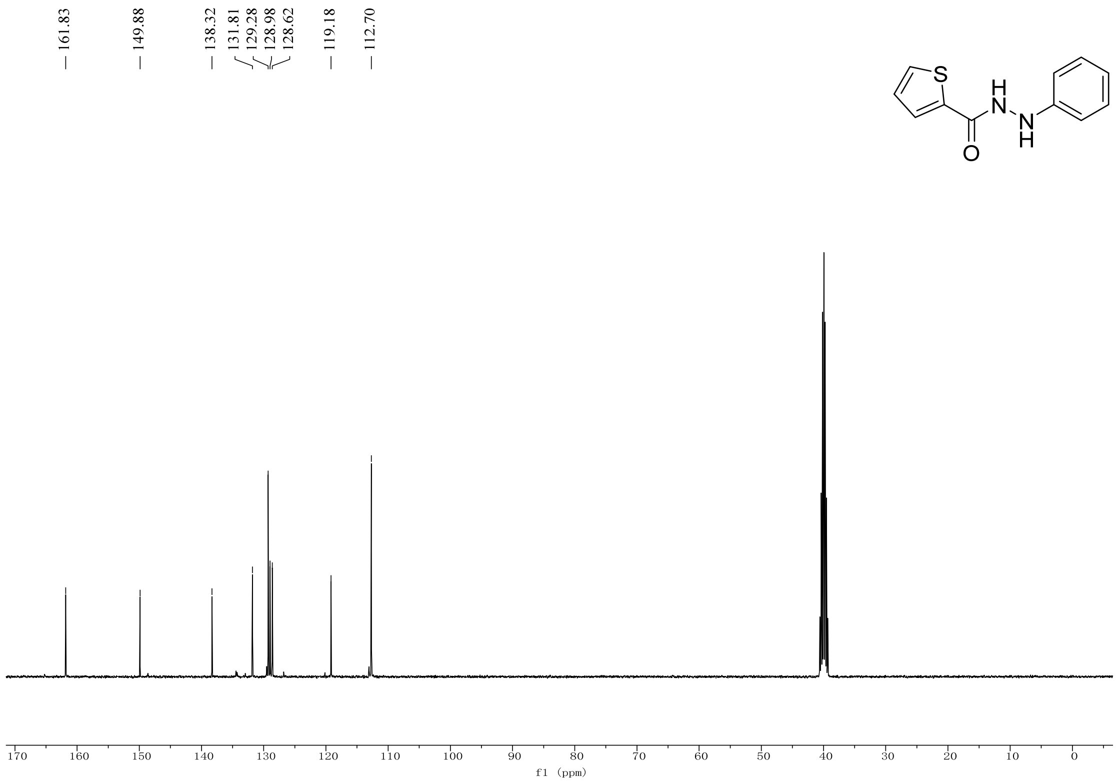

The ^{13}C NMR spectrum of **C**7.

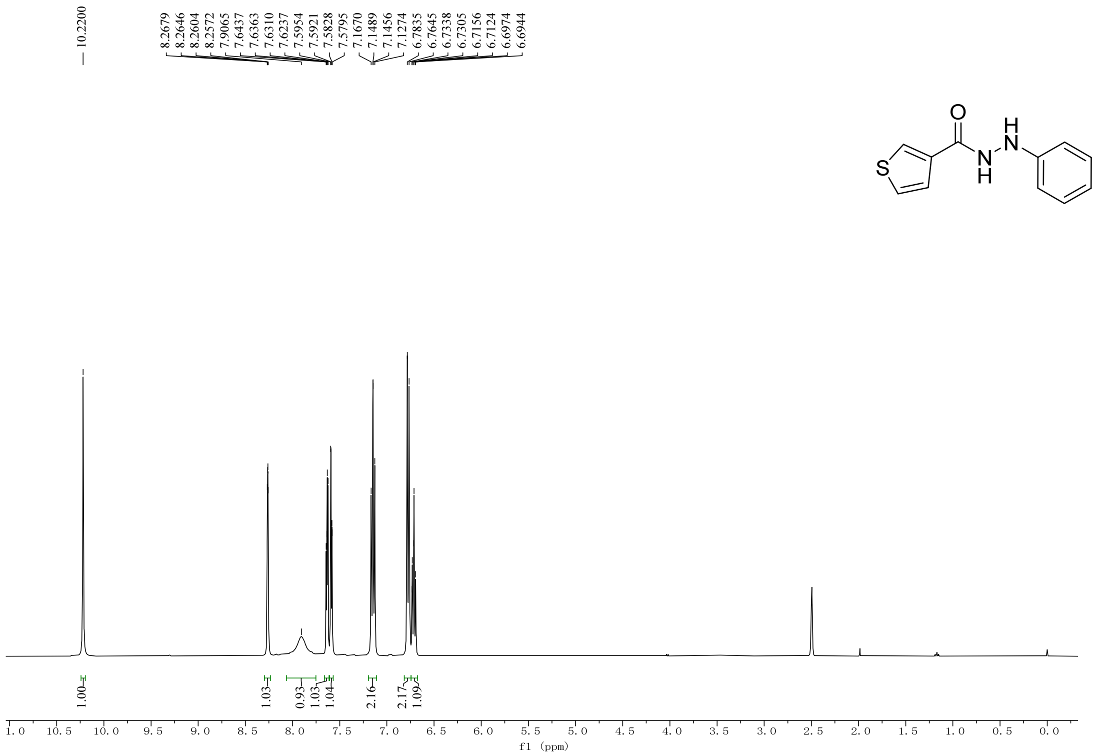

The ^{1}H NMR spectrum of **C**8.

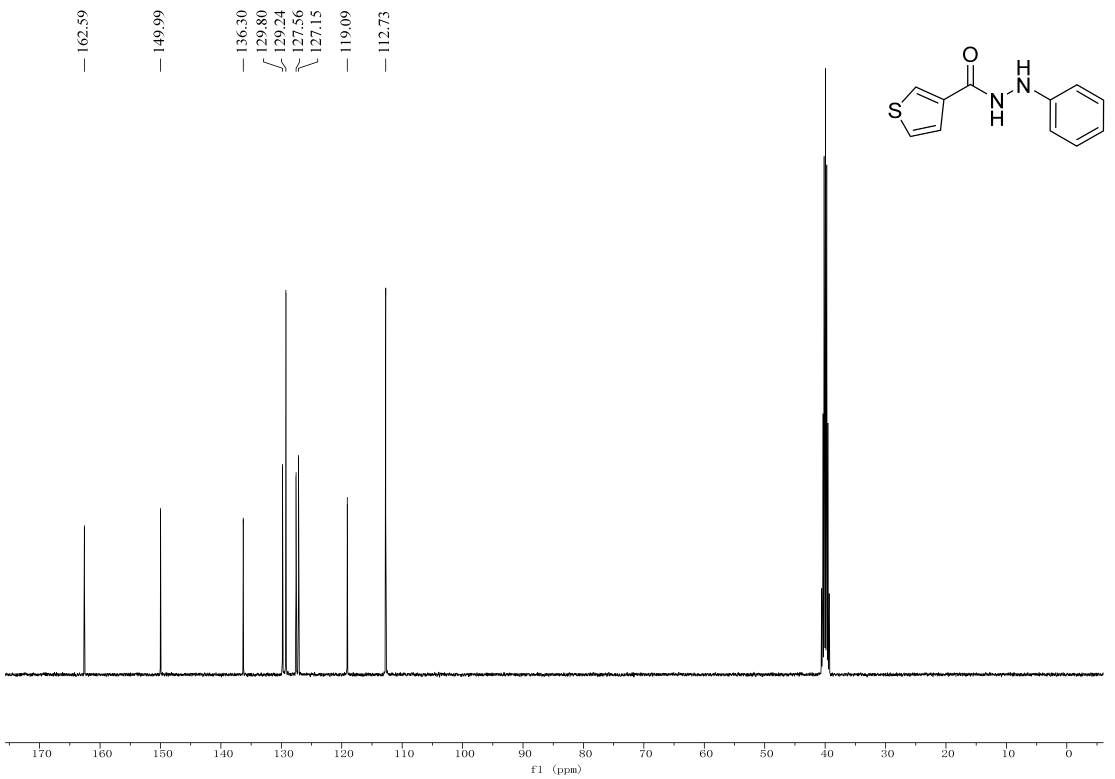

The ^{13}C NMR spectrum of **C$_8$**.

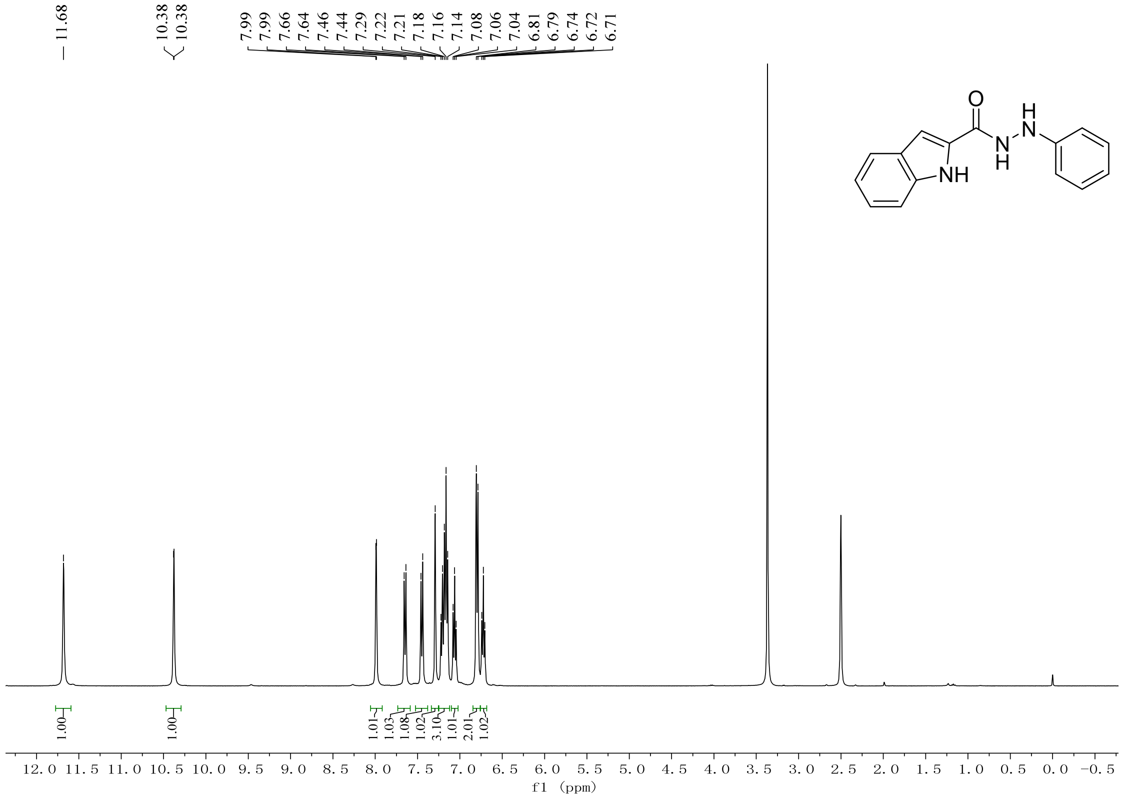

The ^{1}H NMR spectrum of **C$_9$**.

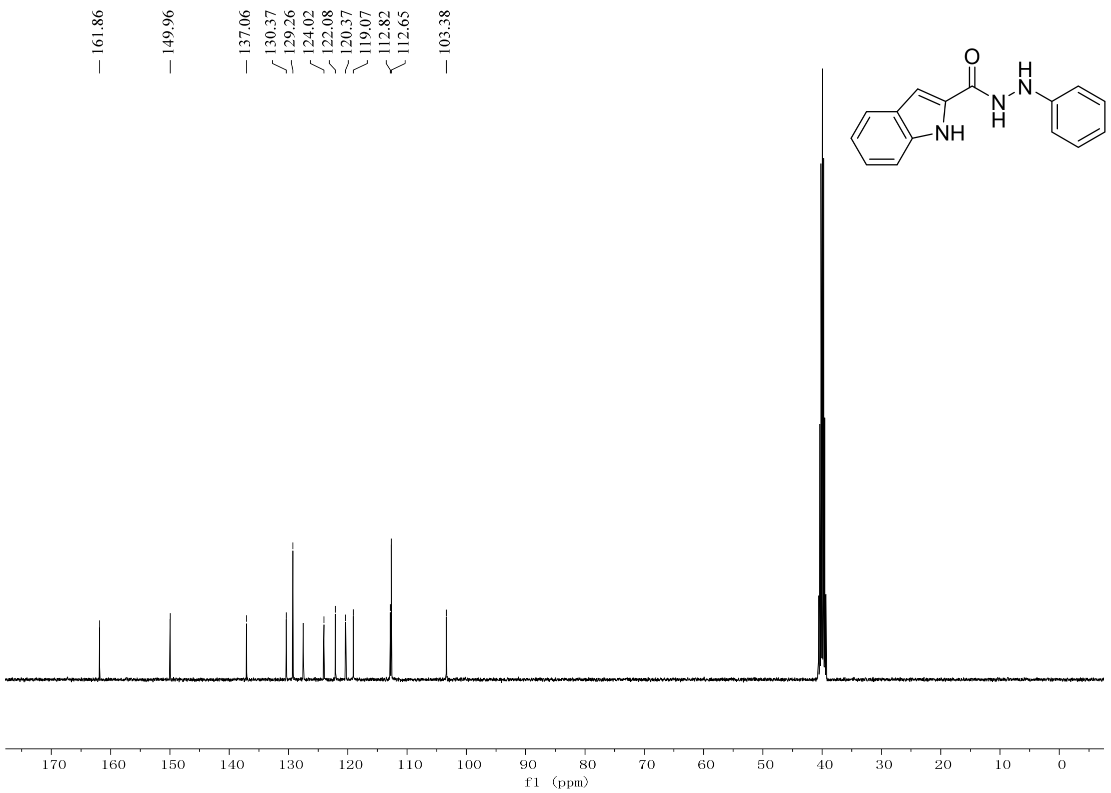

The ^{13}C NMR spectrum of **C9**.

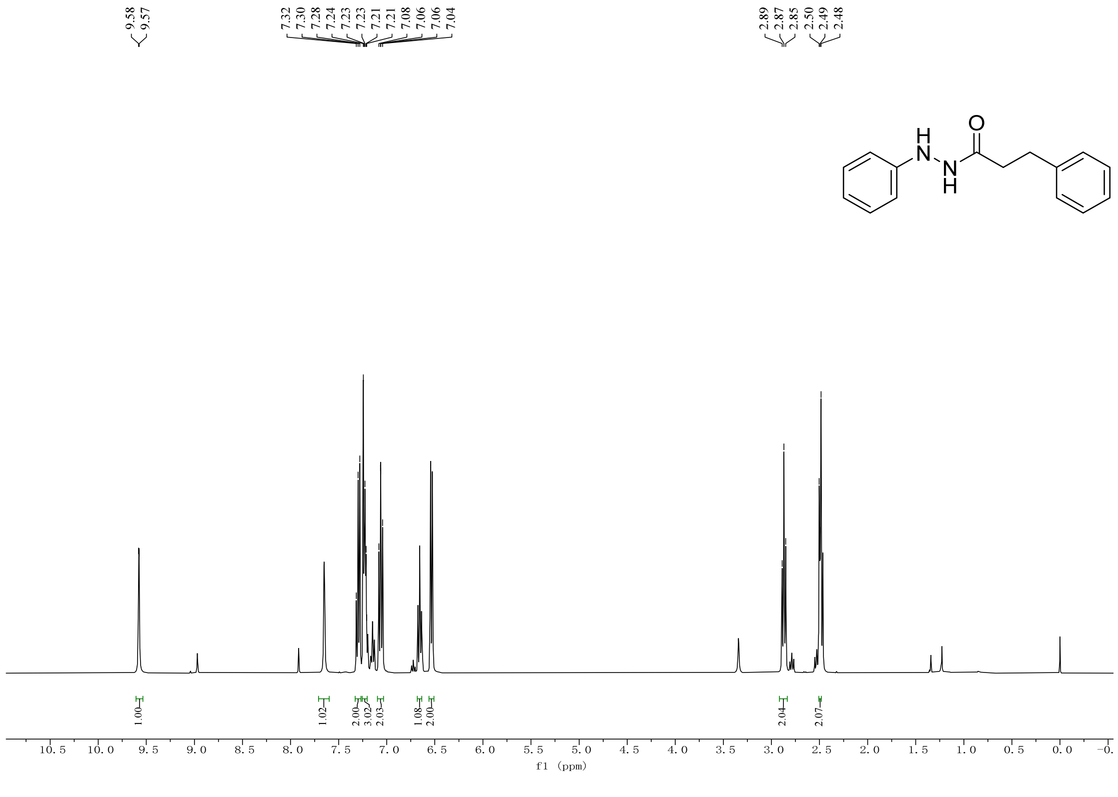

The ^{1}H NMR spectrum of **C10**.

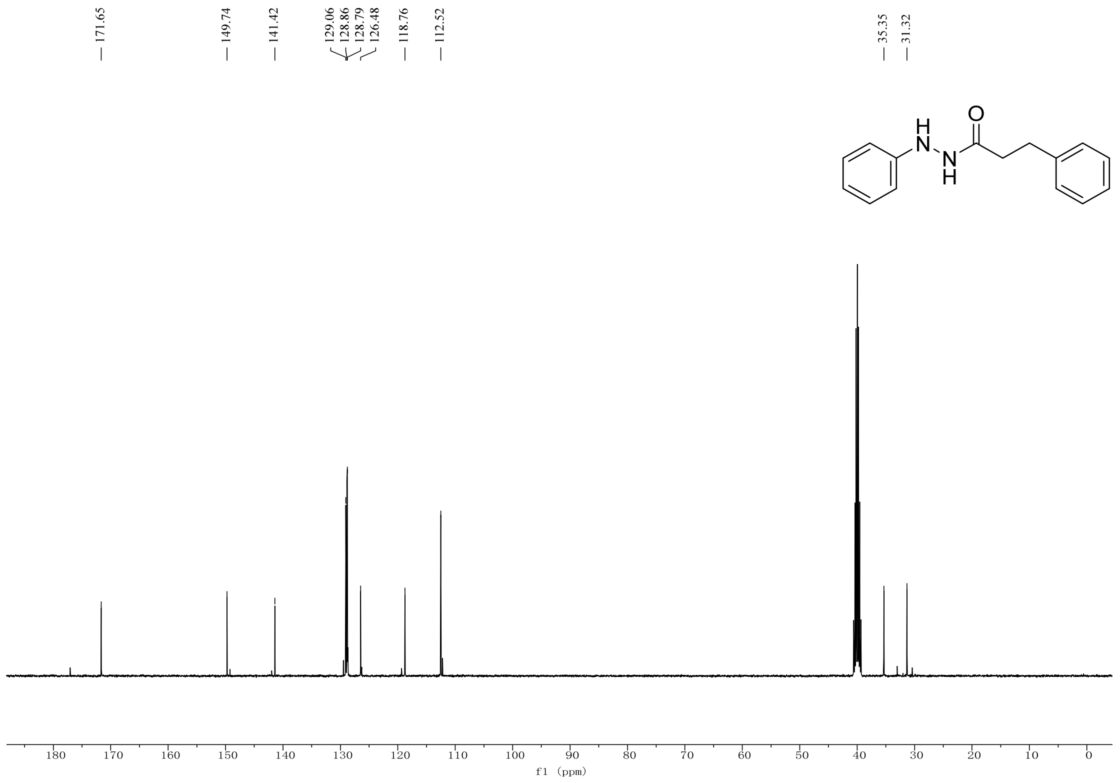

The ^{13}C NMR spectrum of **C10**.

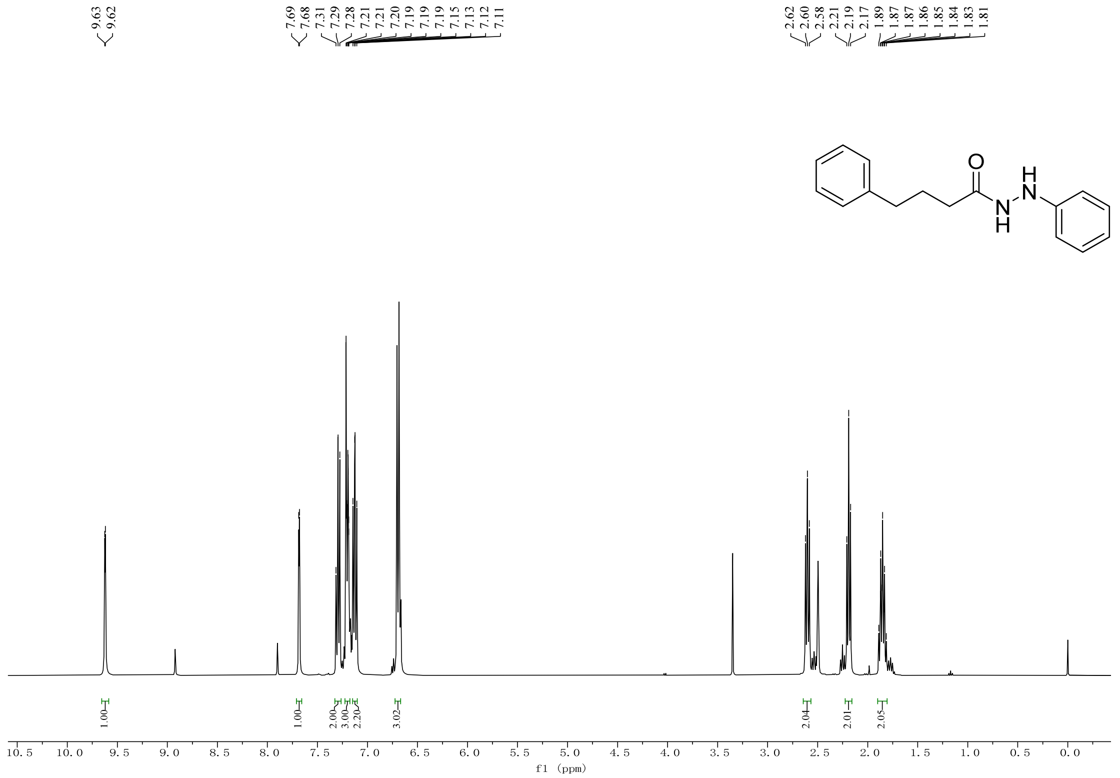

The ^{1}H NMR spectrum of **C11**.

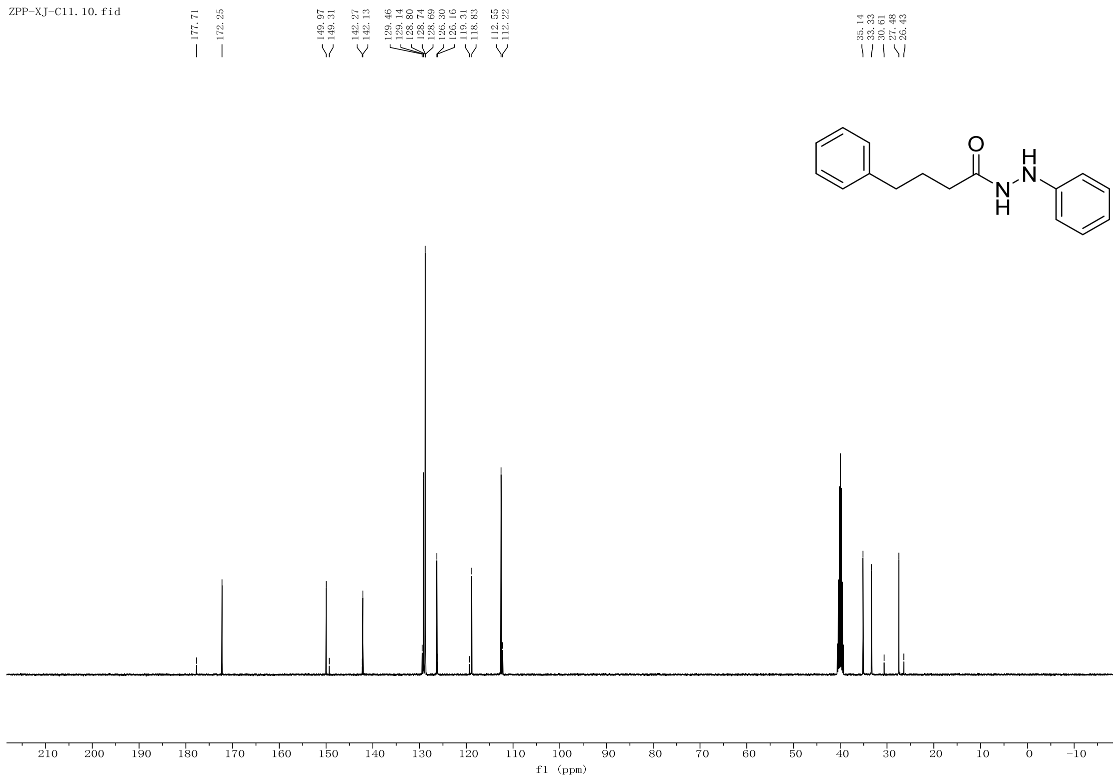

The ^{13}C NMR spectrum of **C11**.

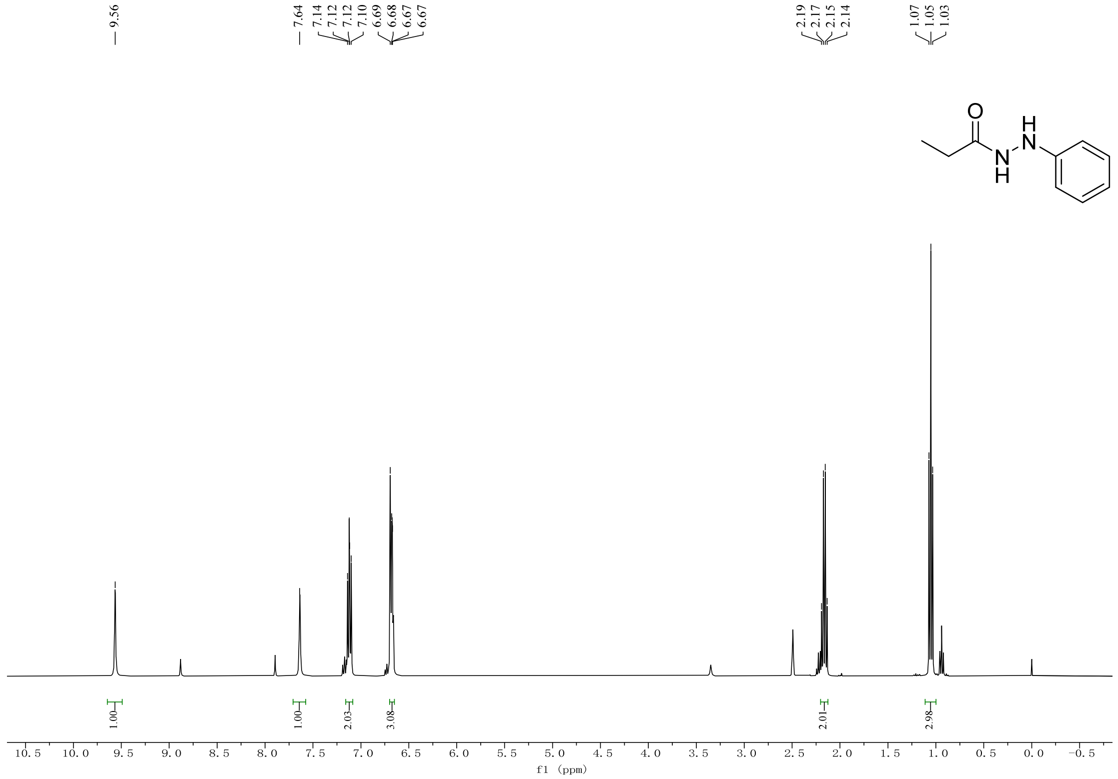

The ^{1}H NMR spectrum of **C12**.

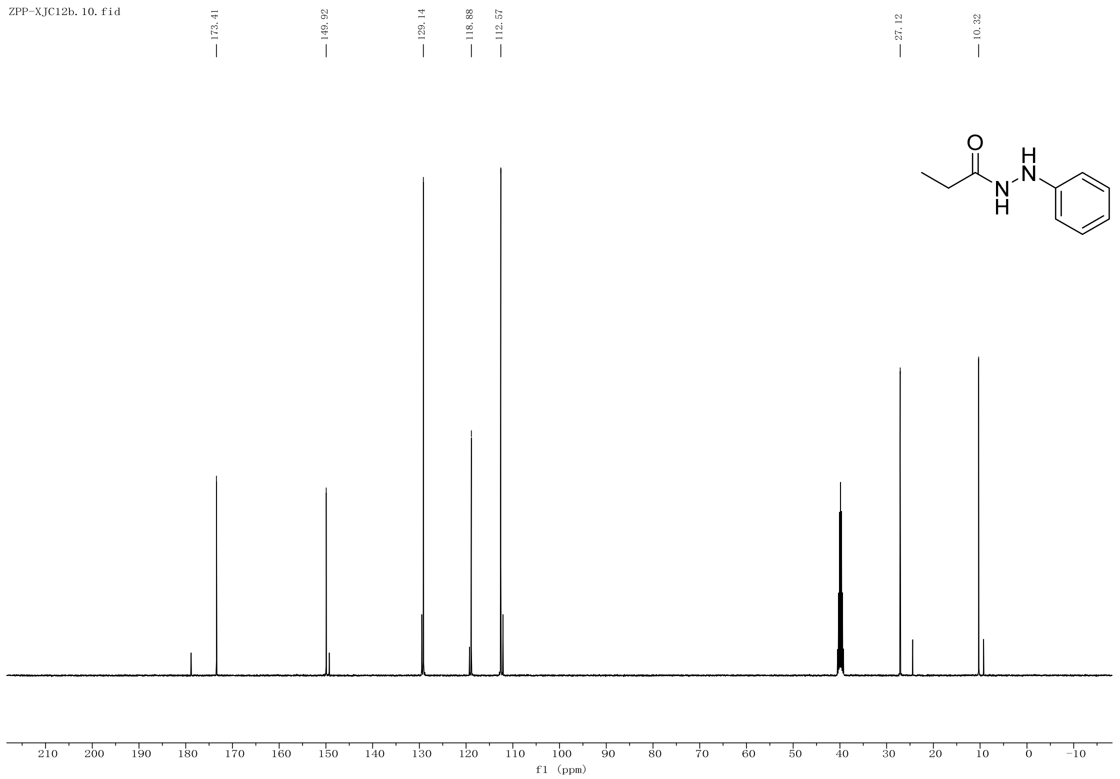

The ^{13}C NMR spectrum of **C12**.

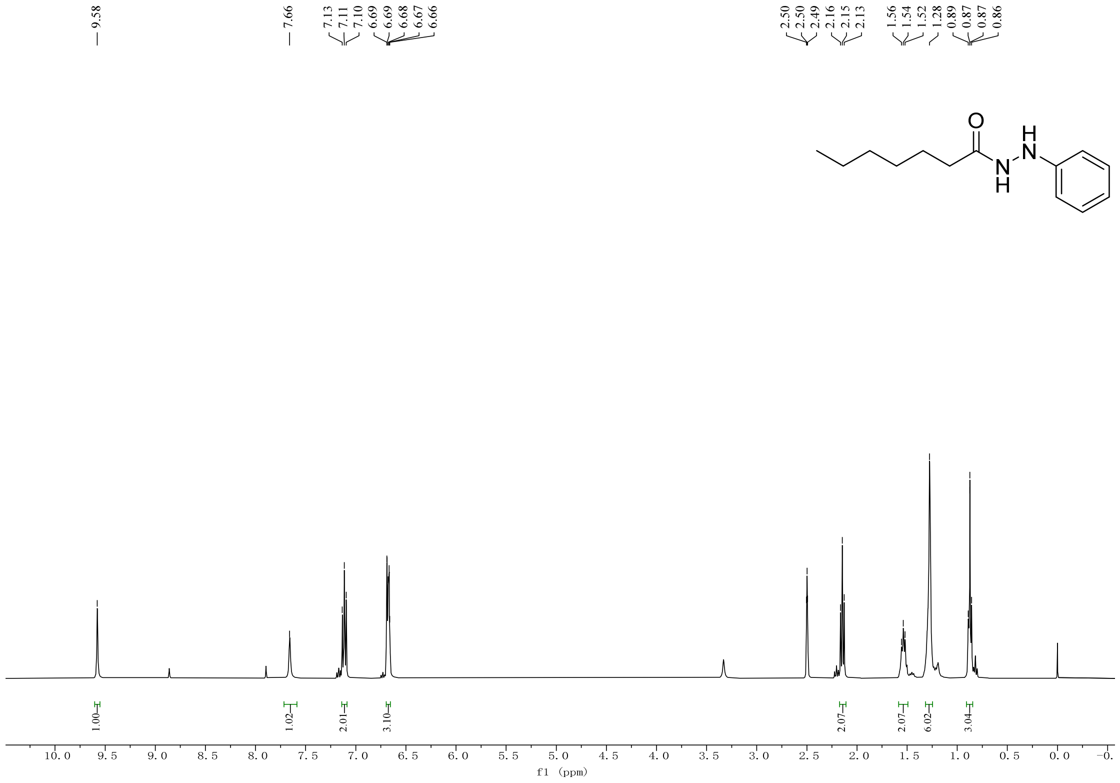

The ^{1}H NMR spectrum of **C13**.

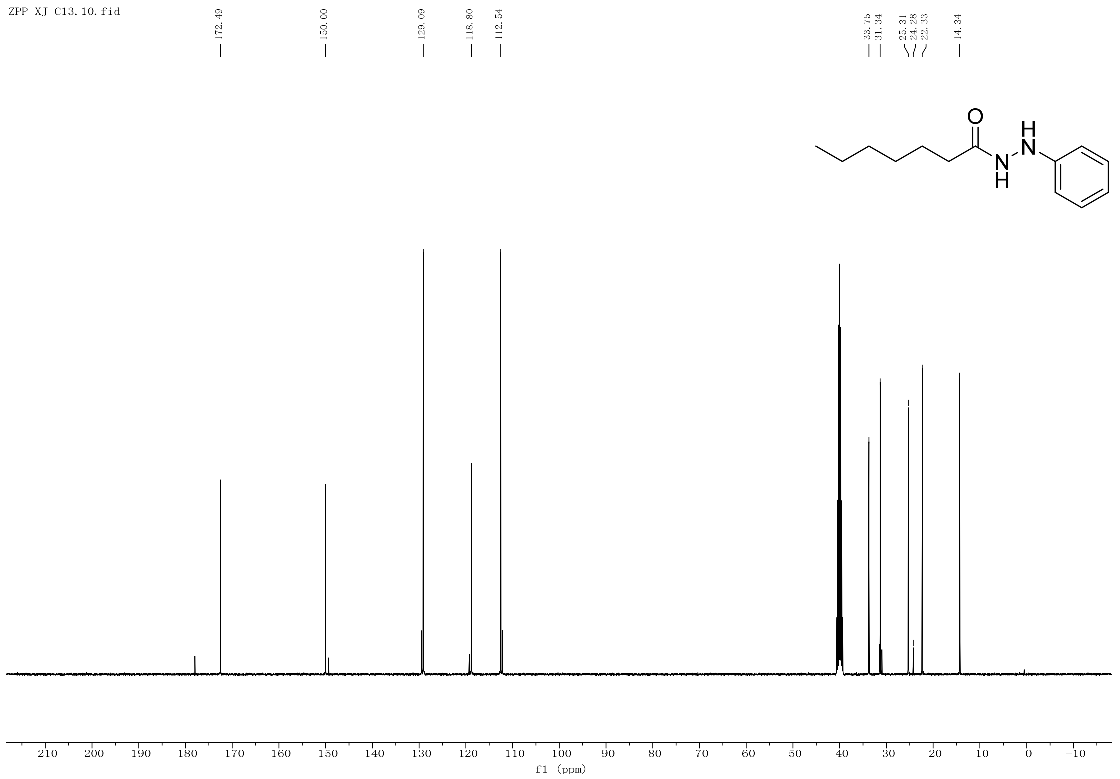

The ^{13}C NMR spectrum of **C13**.

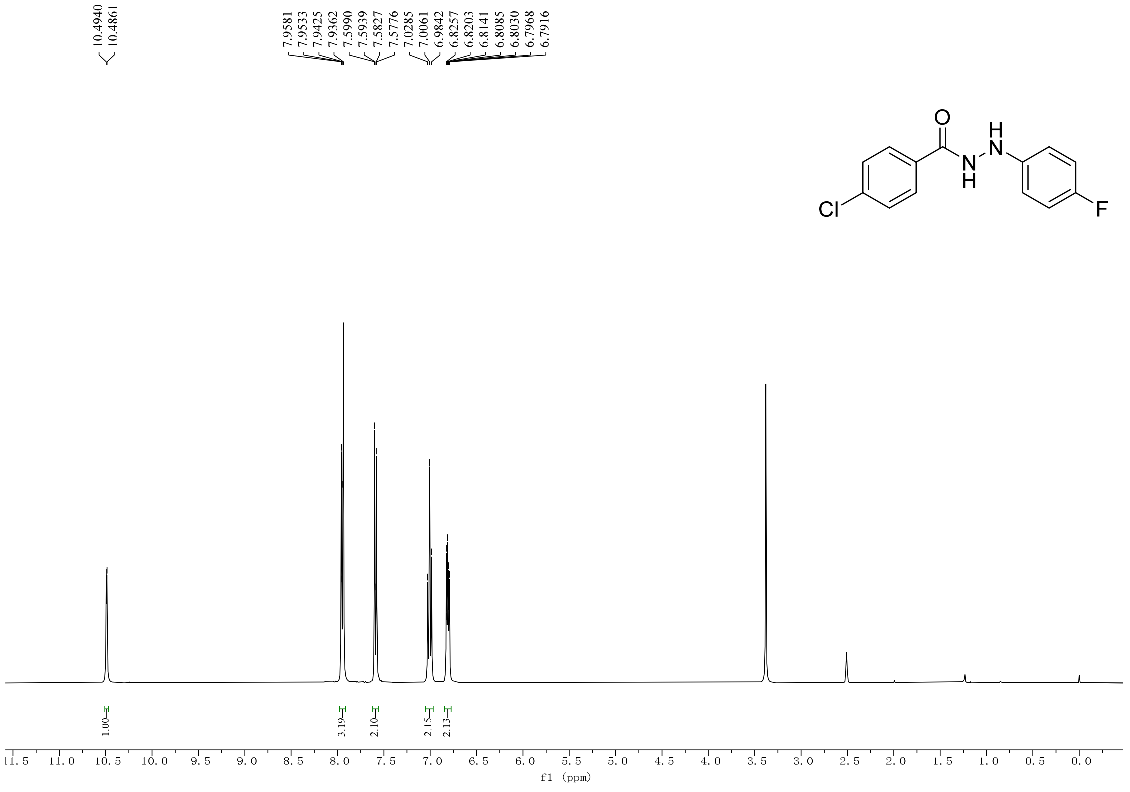

The ^{1}H NMR spectrum of **D1**.

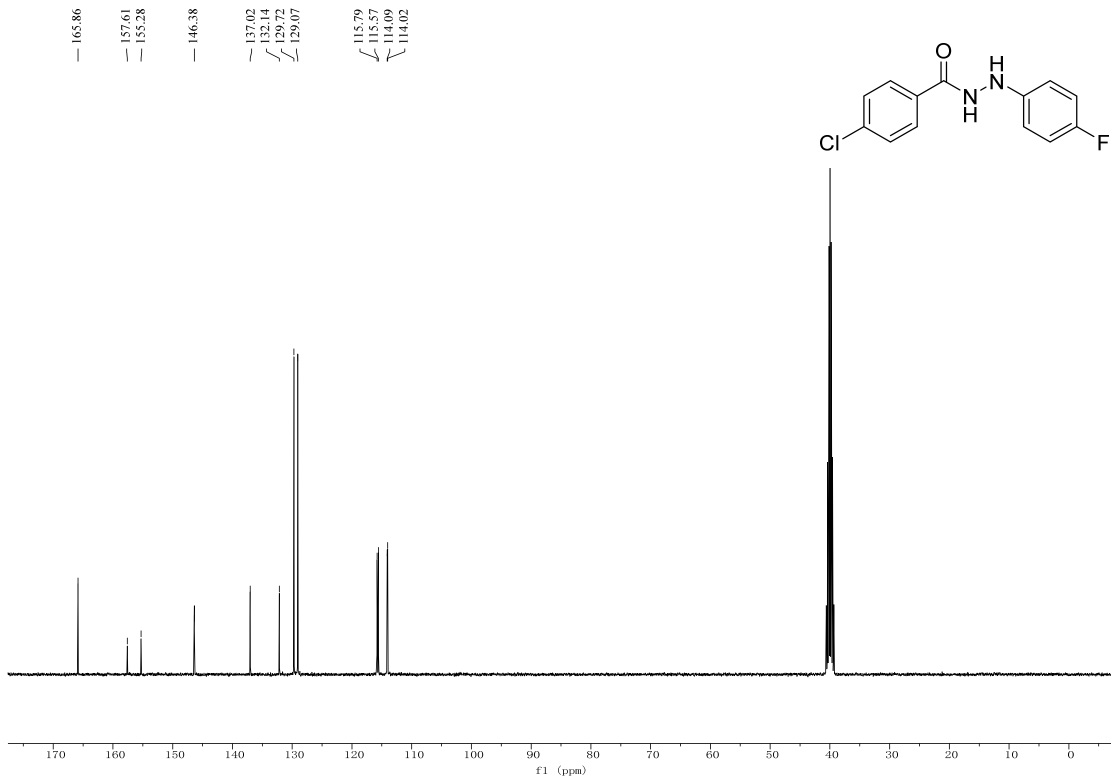

The ^{13}C NMR spectrum of **D1**.

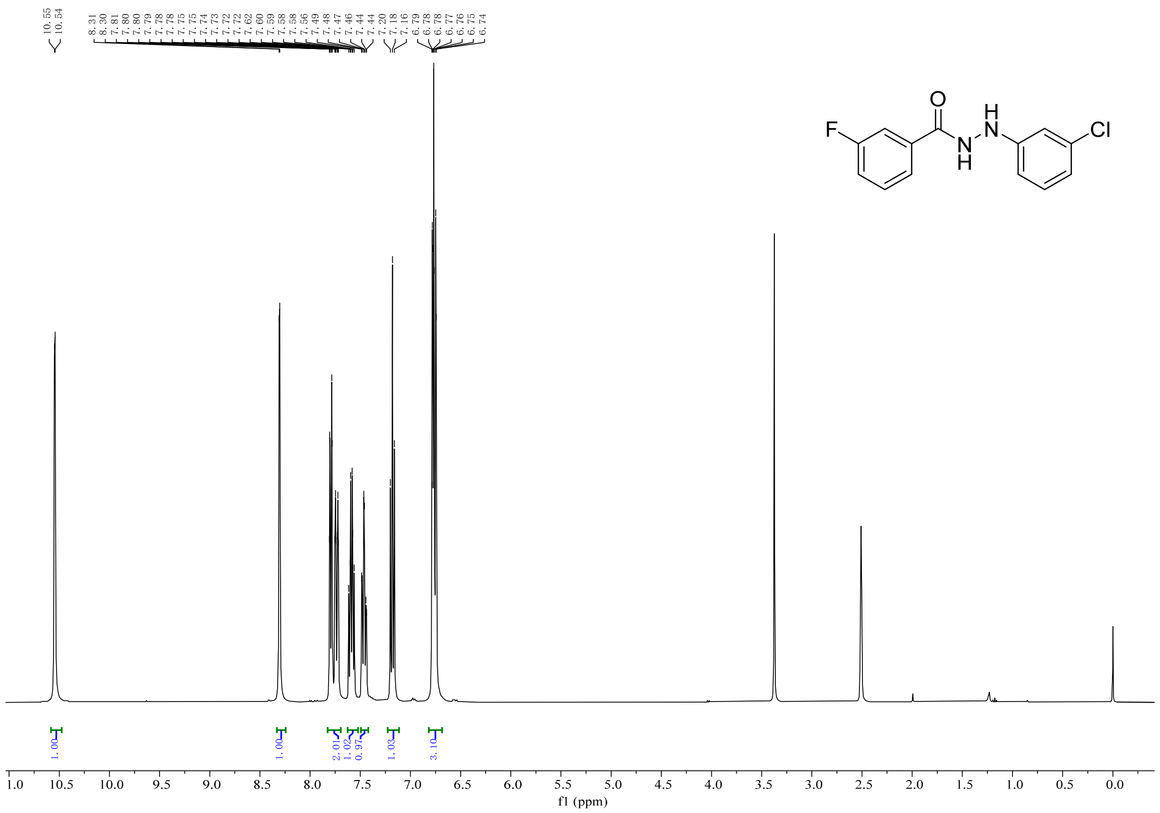

The ^{1}H NMR spectrum of **D2**.

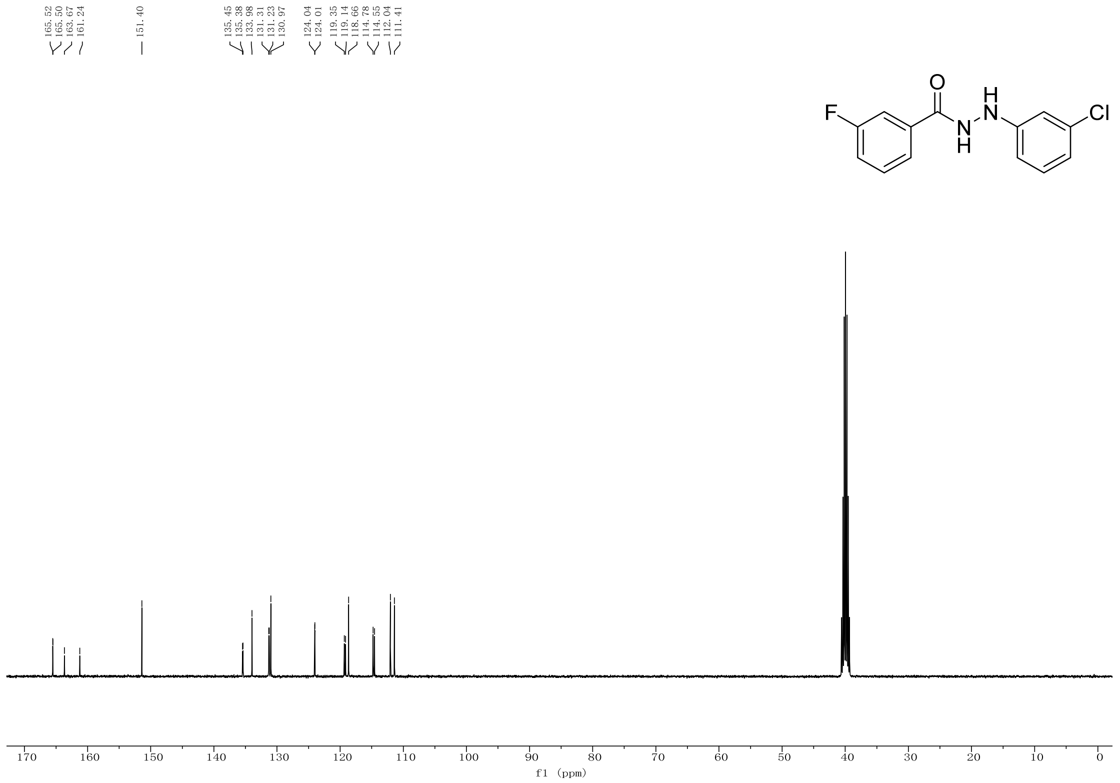

The ^{13}C NMR spectrum of **D₂**.

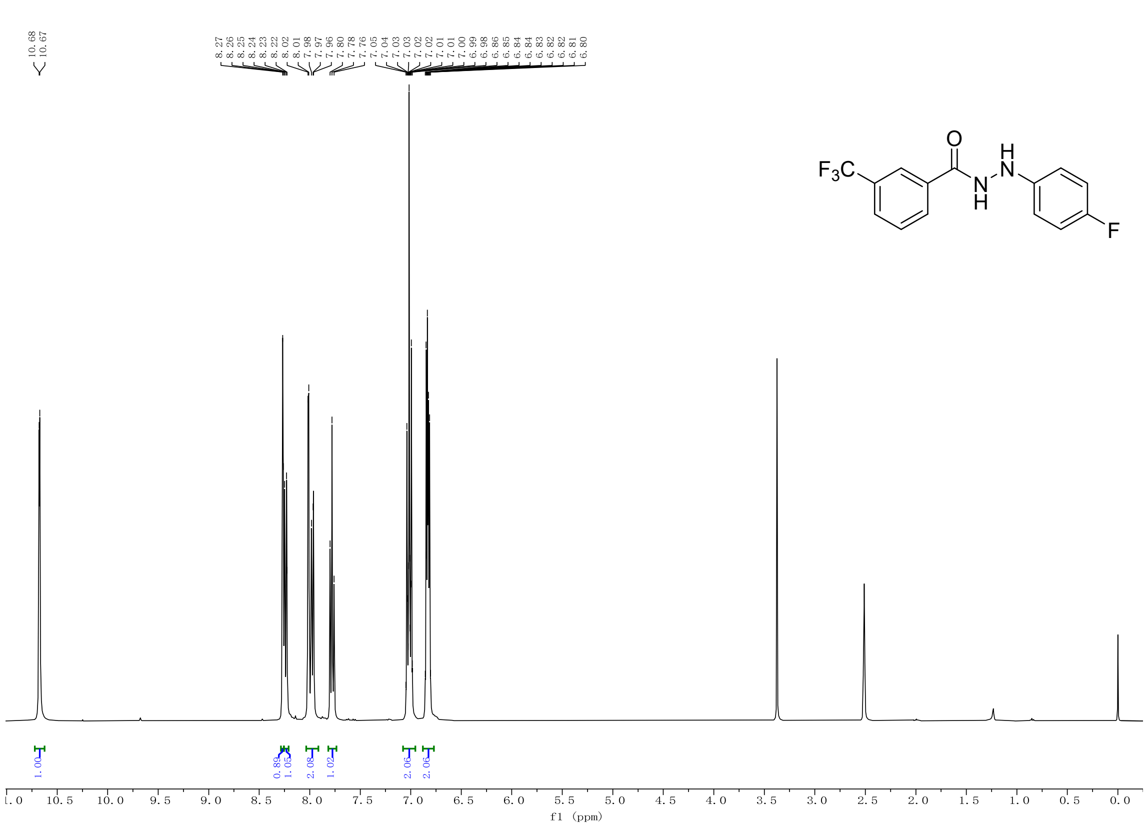

The ^{1}H NMR spectrum of **D₃**.

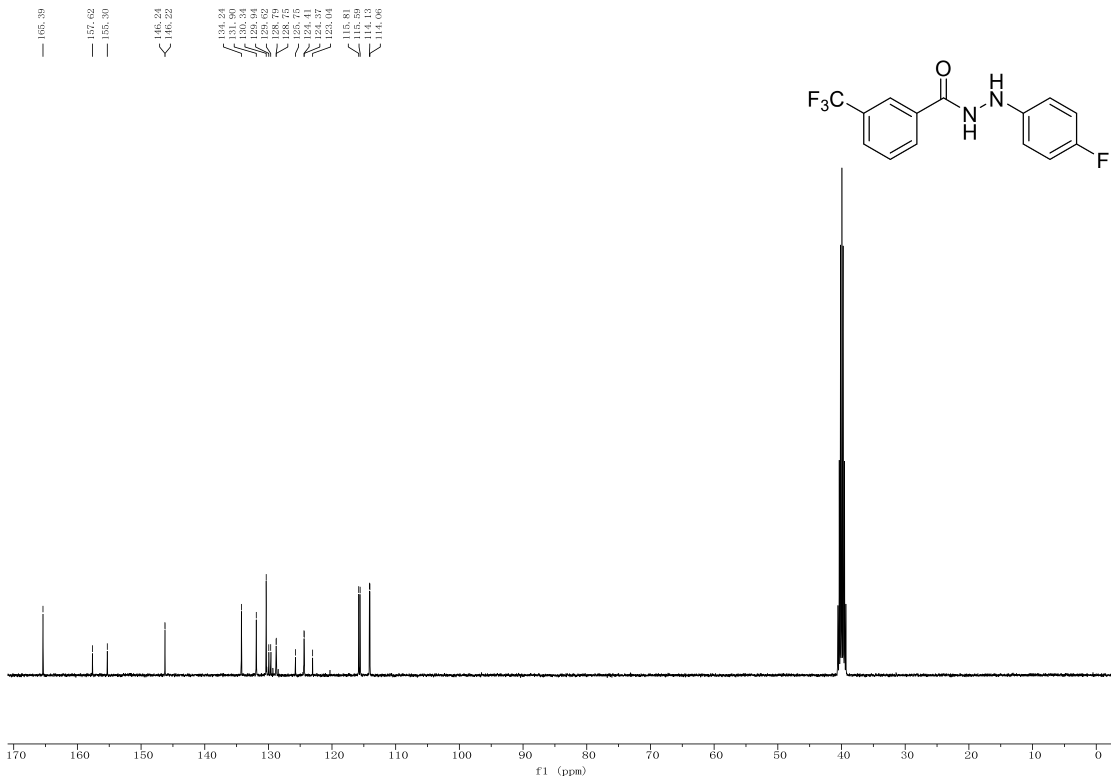

The ^{13}C NMR spectrum of **D3**.

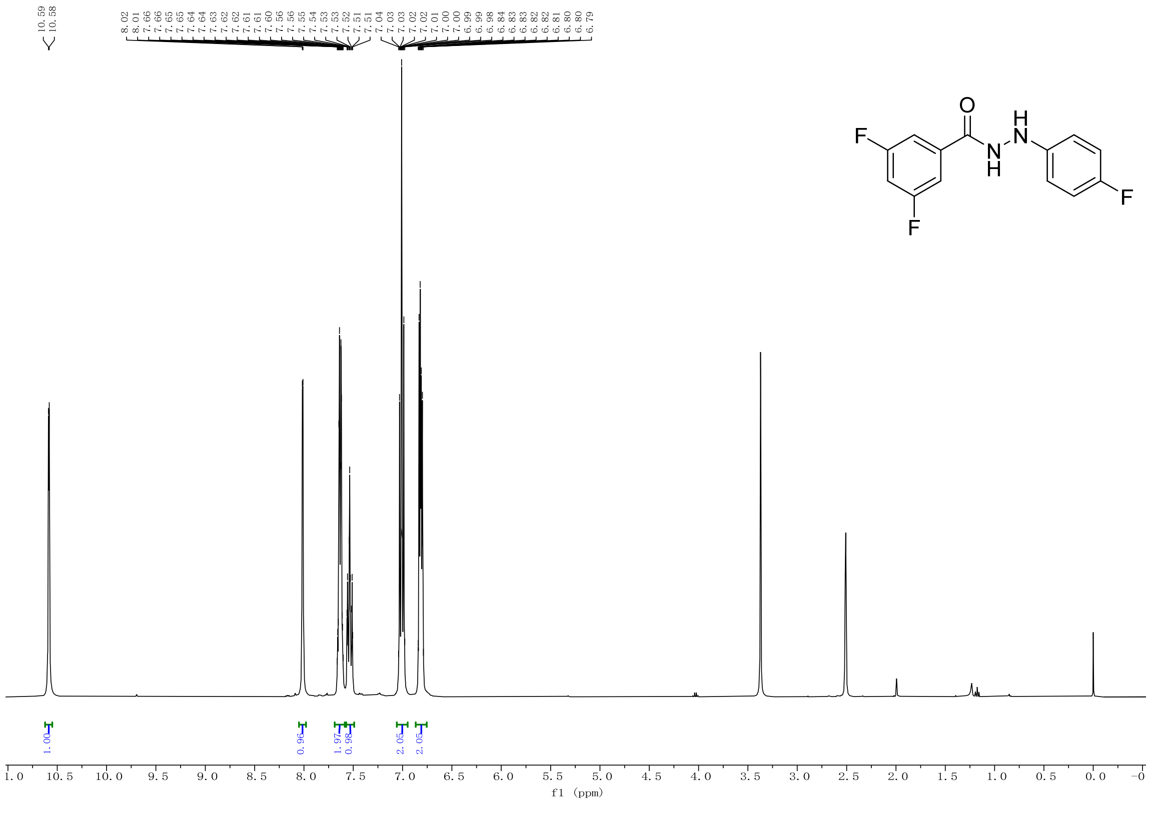

The ^{1}H NMR spectrum of **D4**.

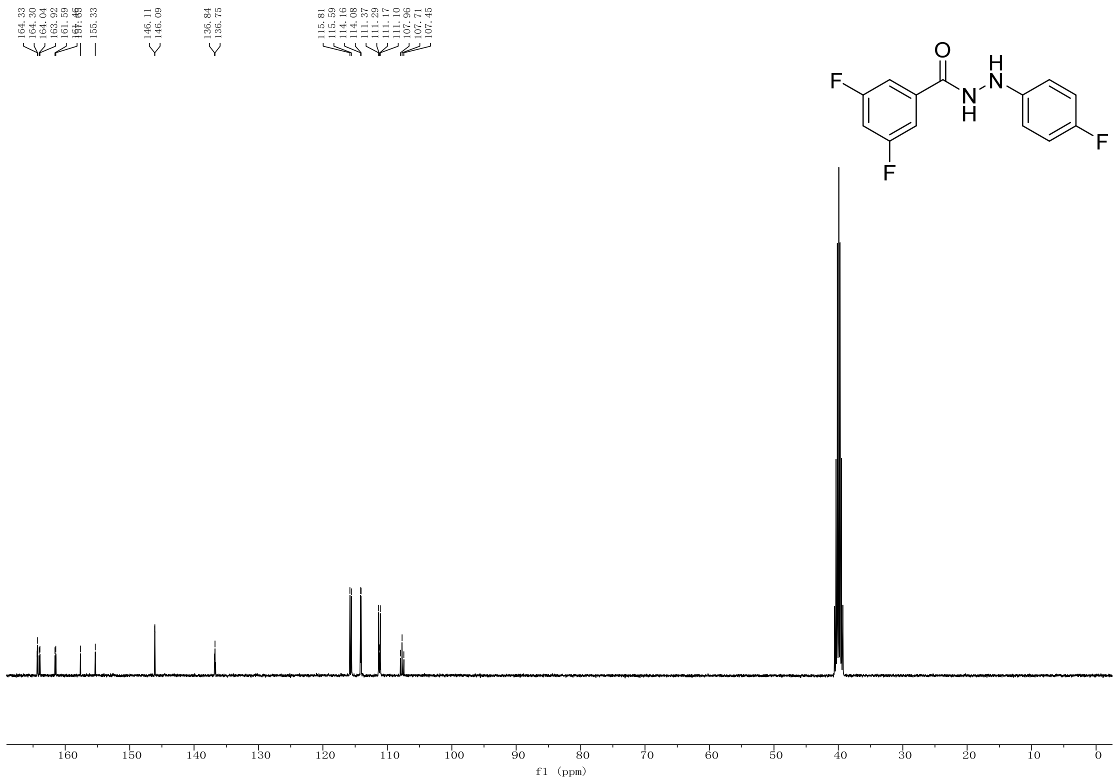

The ^{13}C NMR spectrum of **D4**.

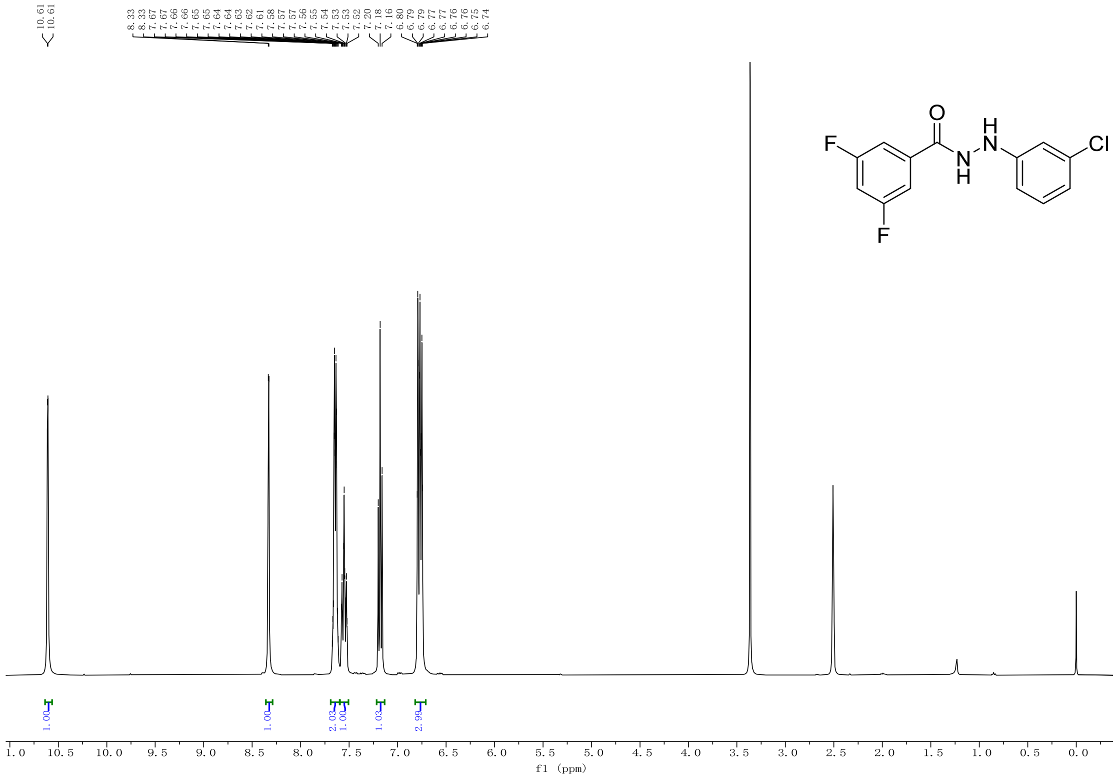

The ^{1}H NMR spectrum of **D5**.

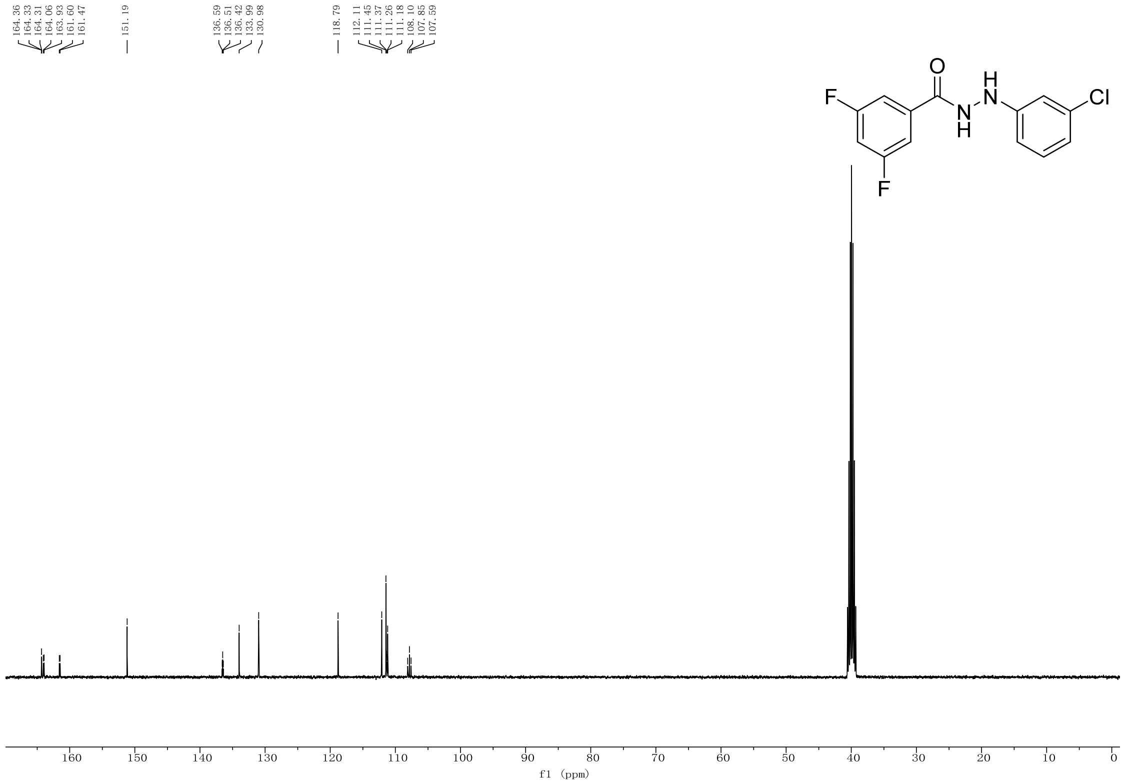

The ^{13}C NMR spectrum of **D5**.

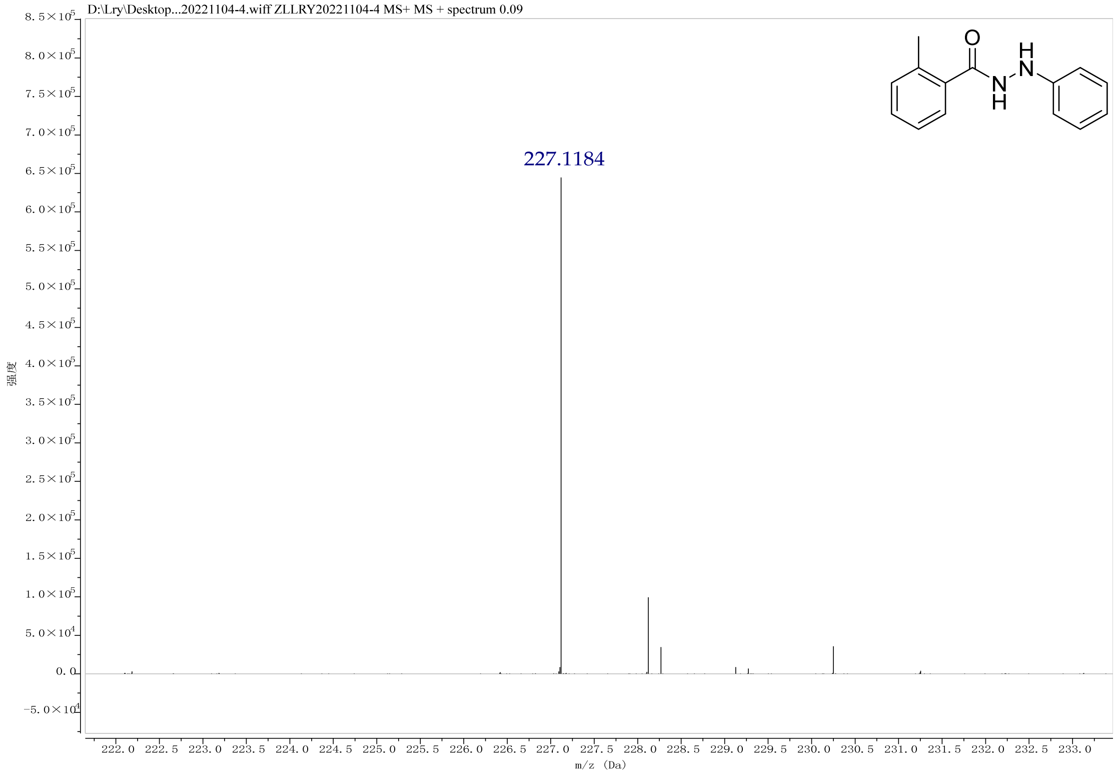

The HRMS spectrum of **A3**.

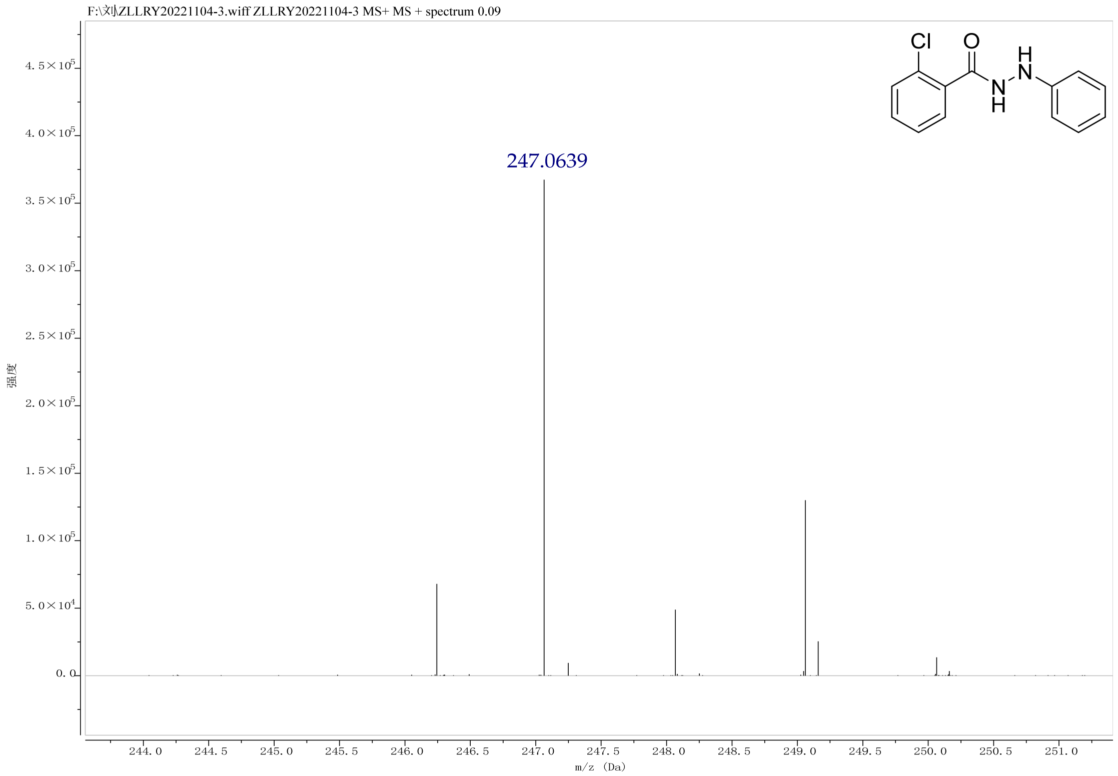

The HRMS spectrum of **A5**.

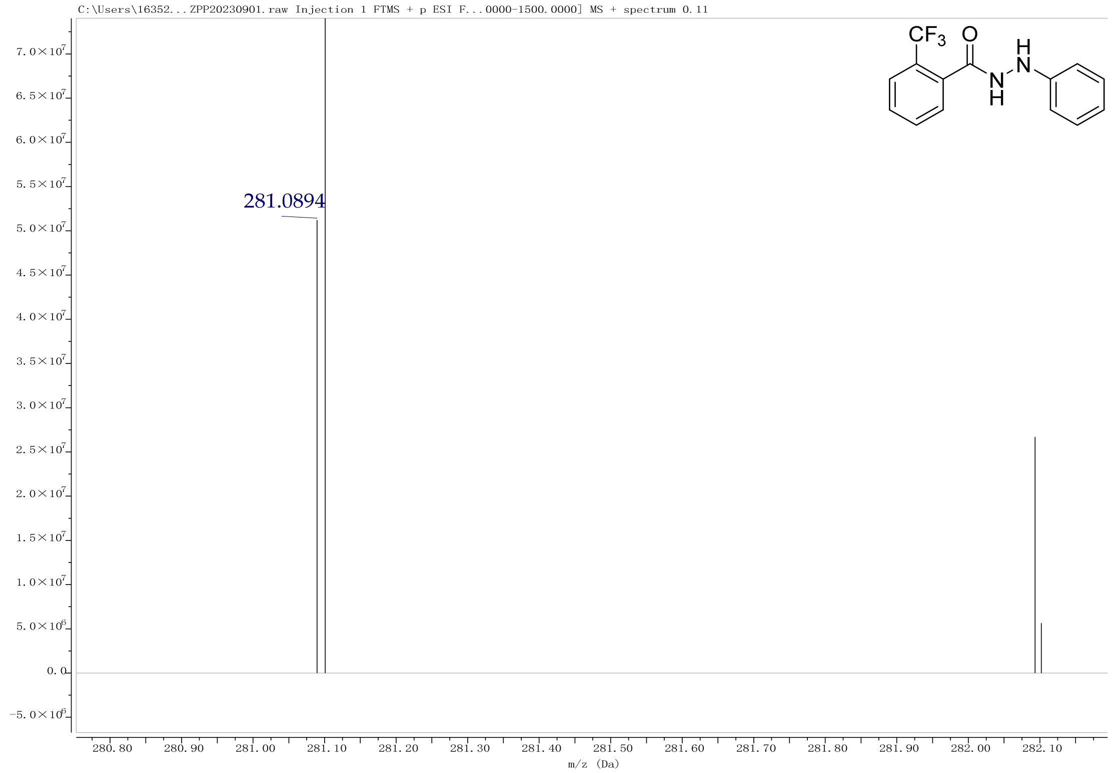

The HRMS spectrum of **A6**.

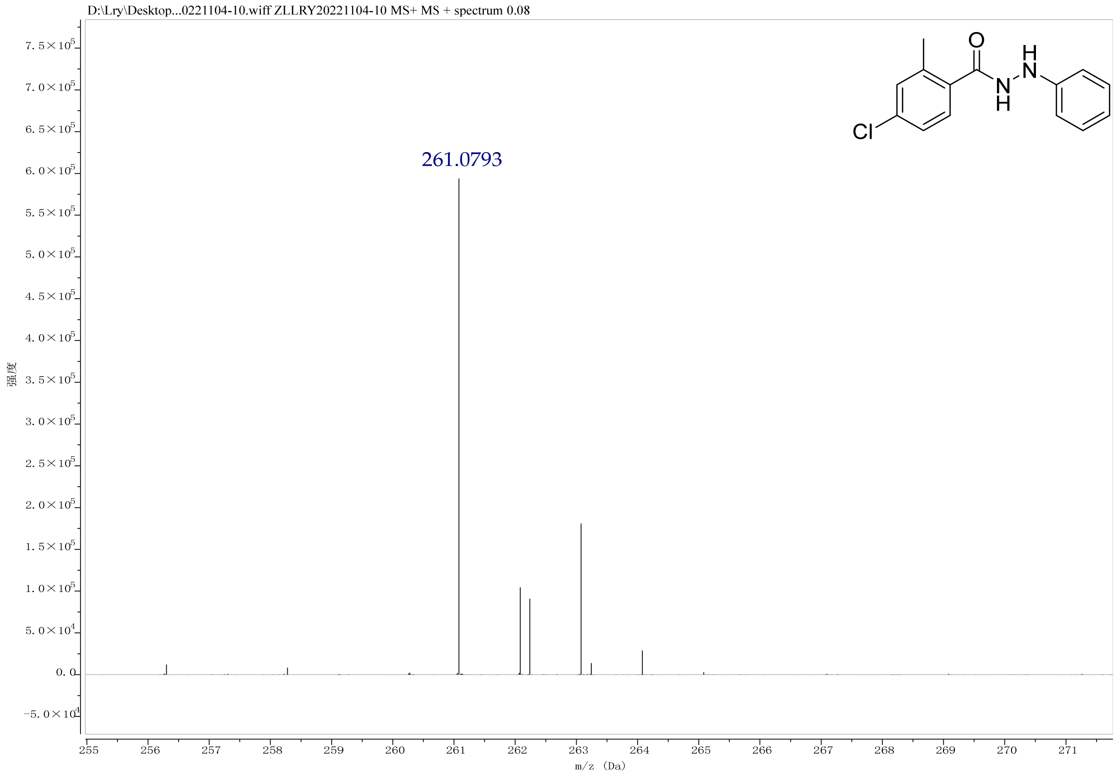

The HRMS spectrum of **A7**.

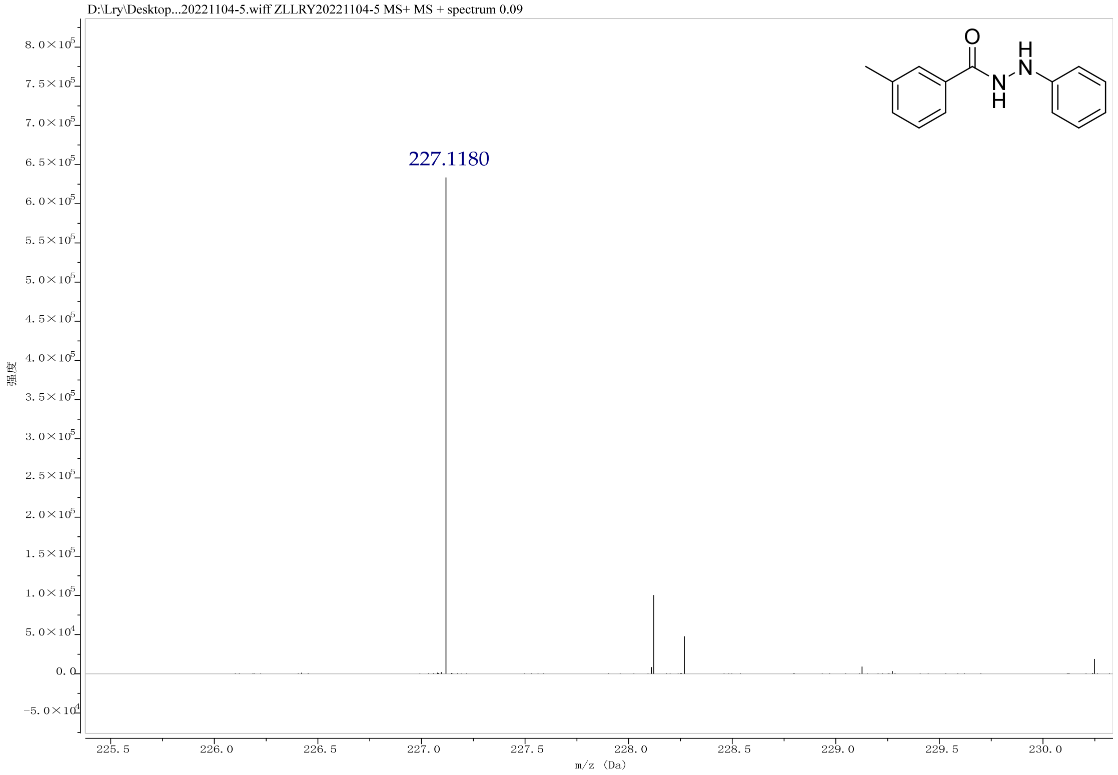

The HRMS spectrum of **A9**.

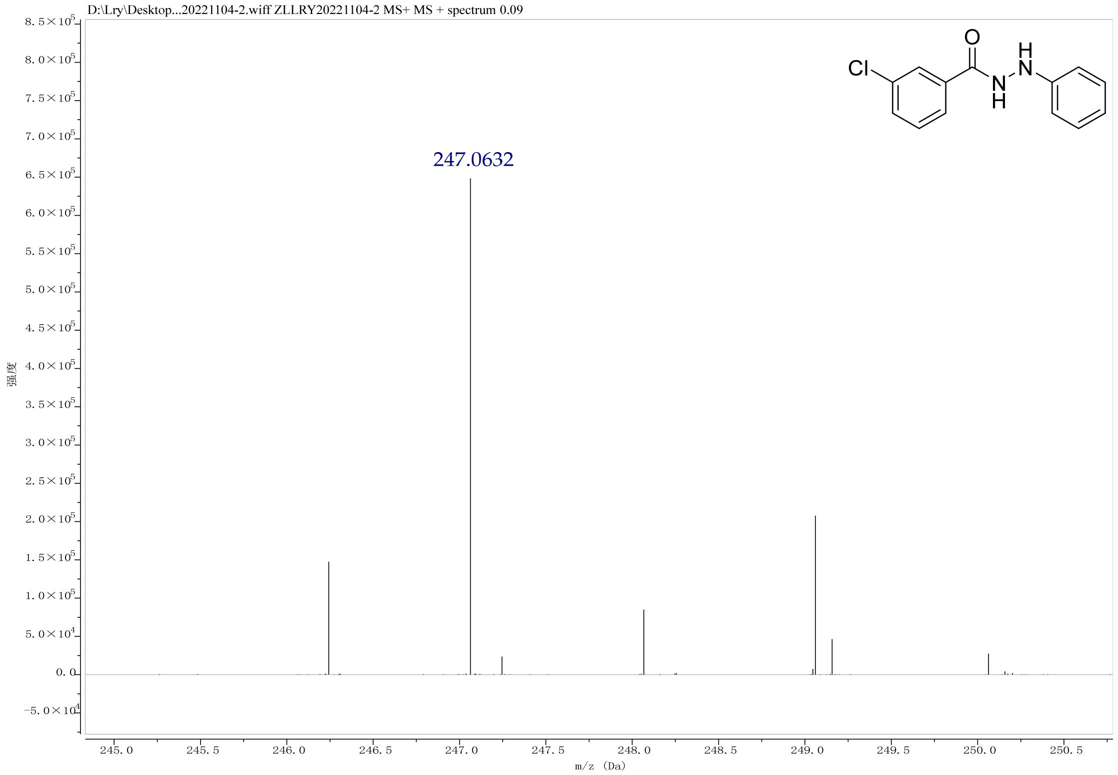

The HRMS spectrum of **A10**.

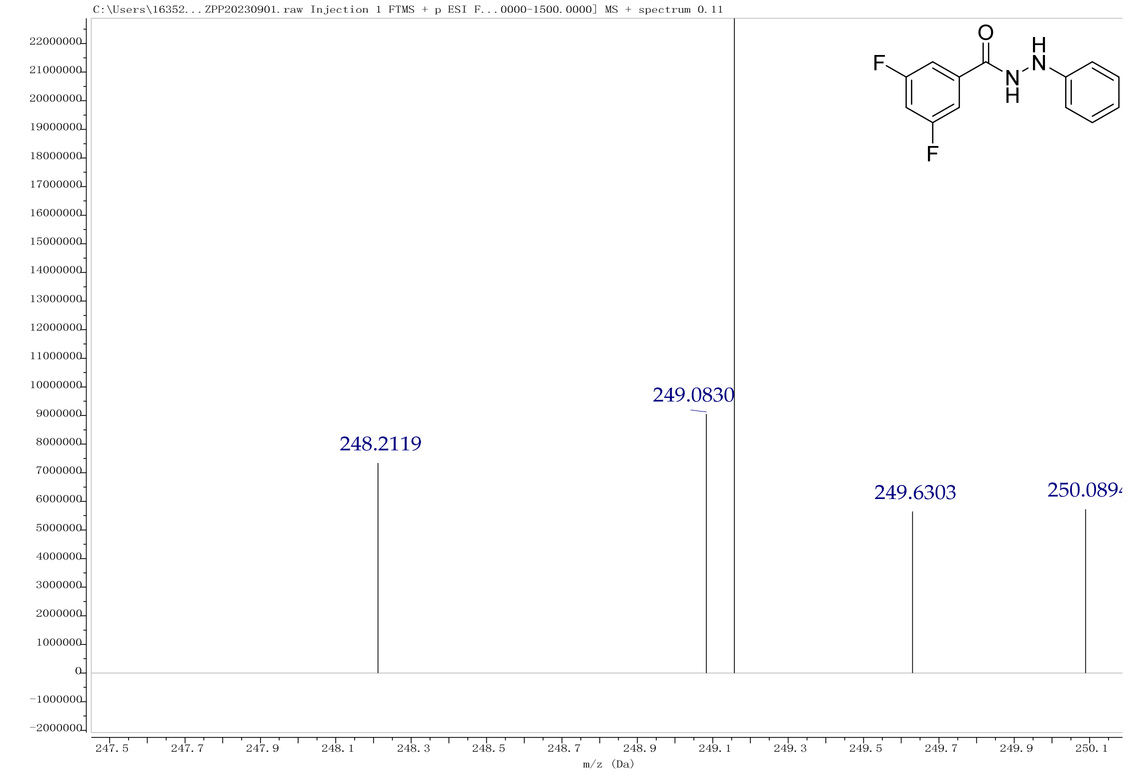

The HRMS spectrum of **A12**.

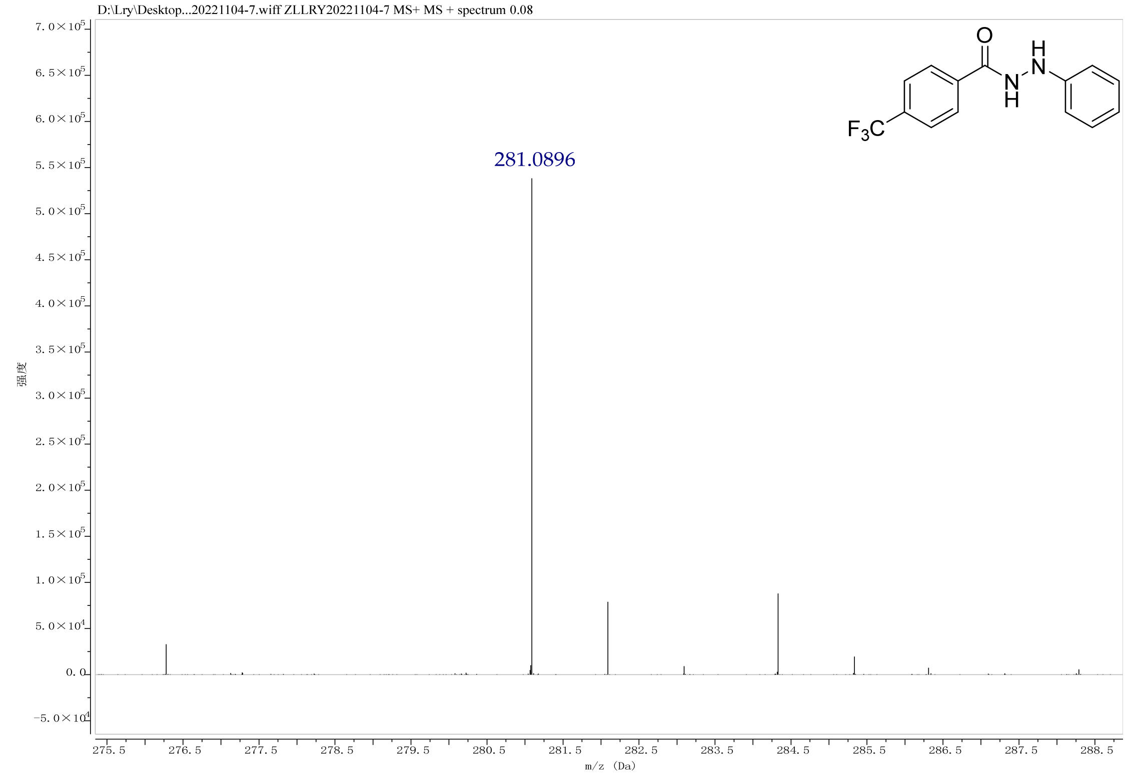

The HRMS spectrum of **A17**.

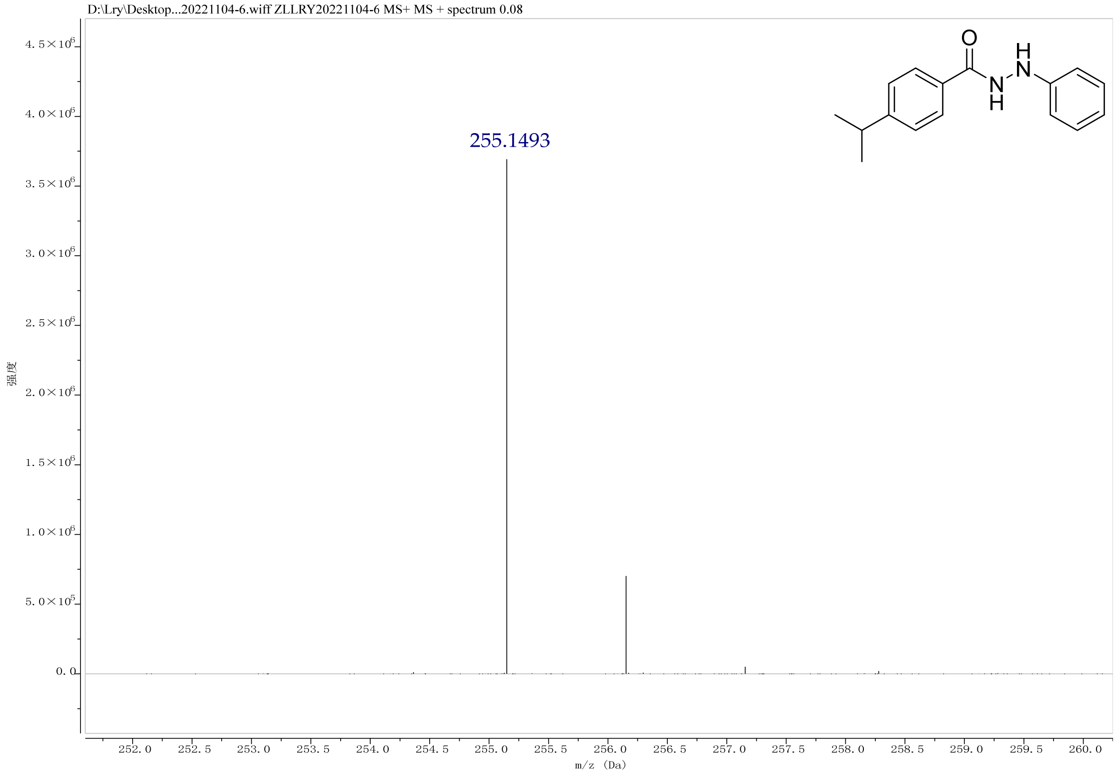

The HRMS spectrum of **A19**.

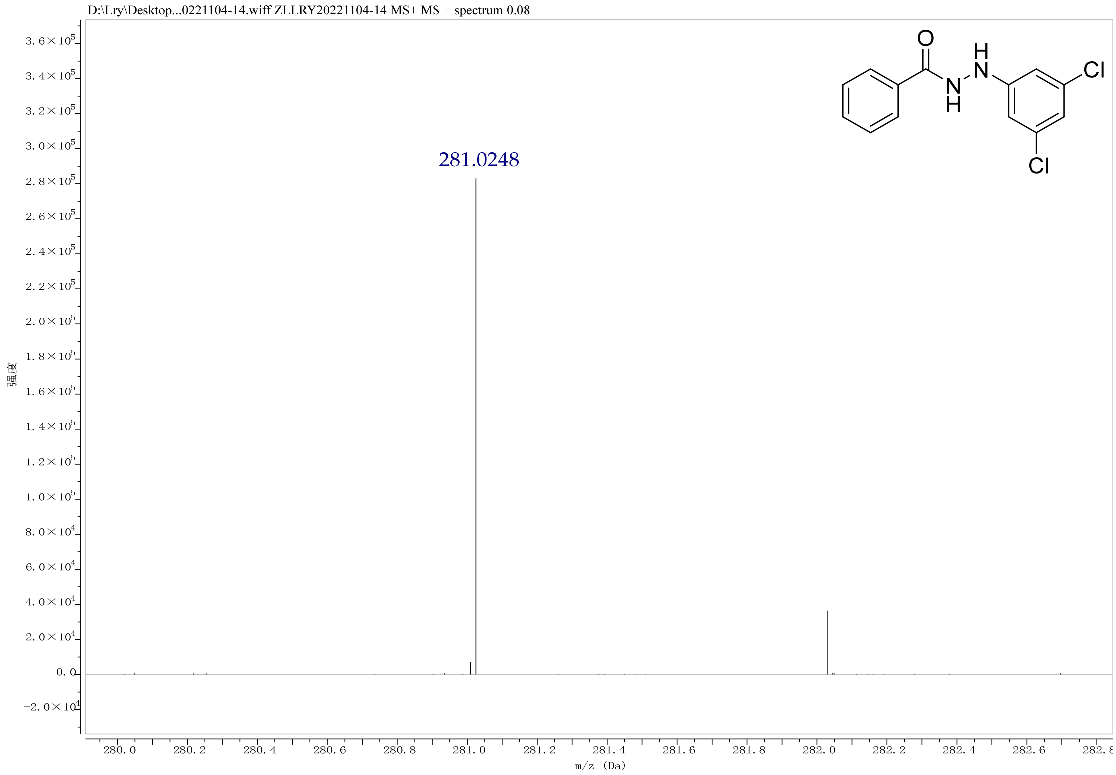

The HRMS spectrum of **B2**.

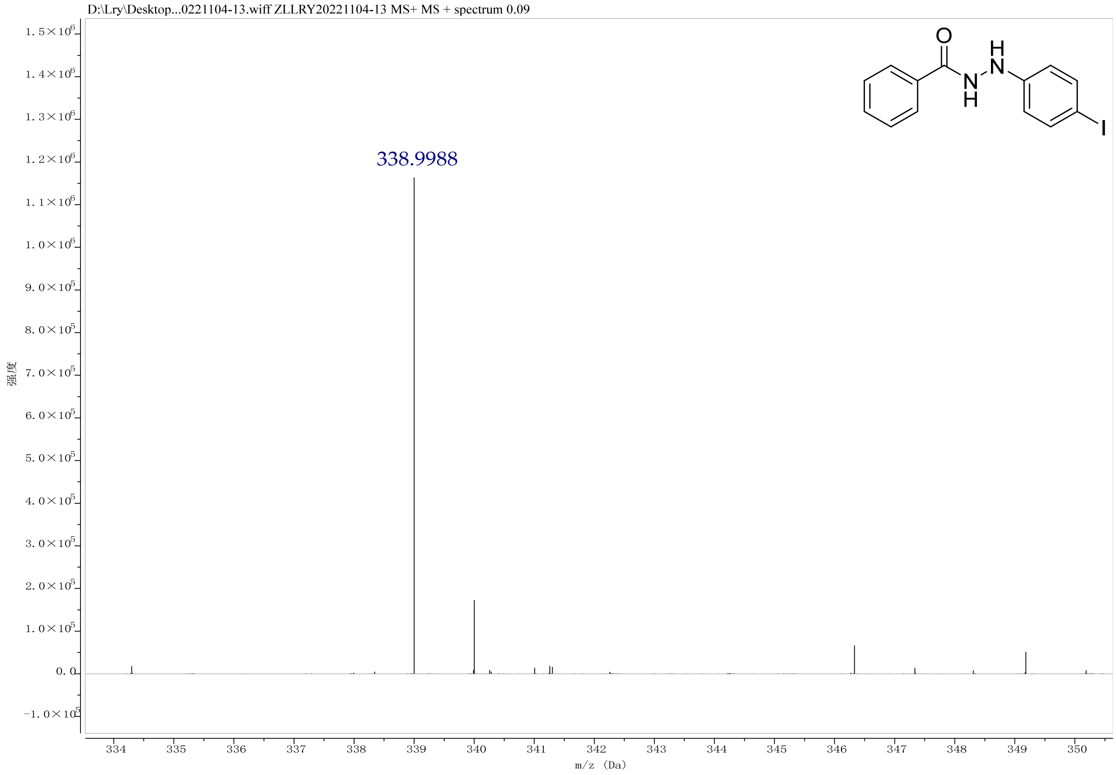

The HRMS spectrum of **B₆**.

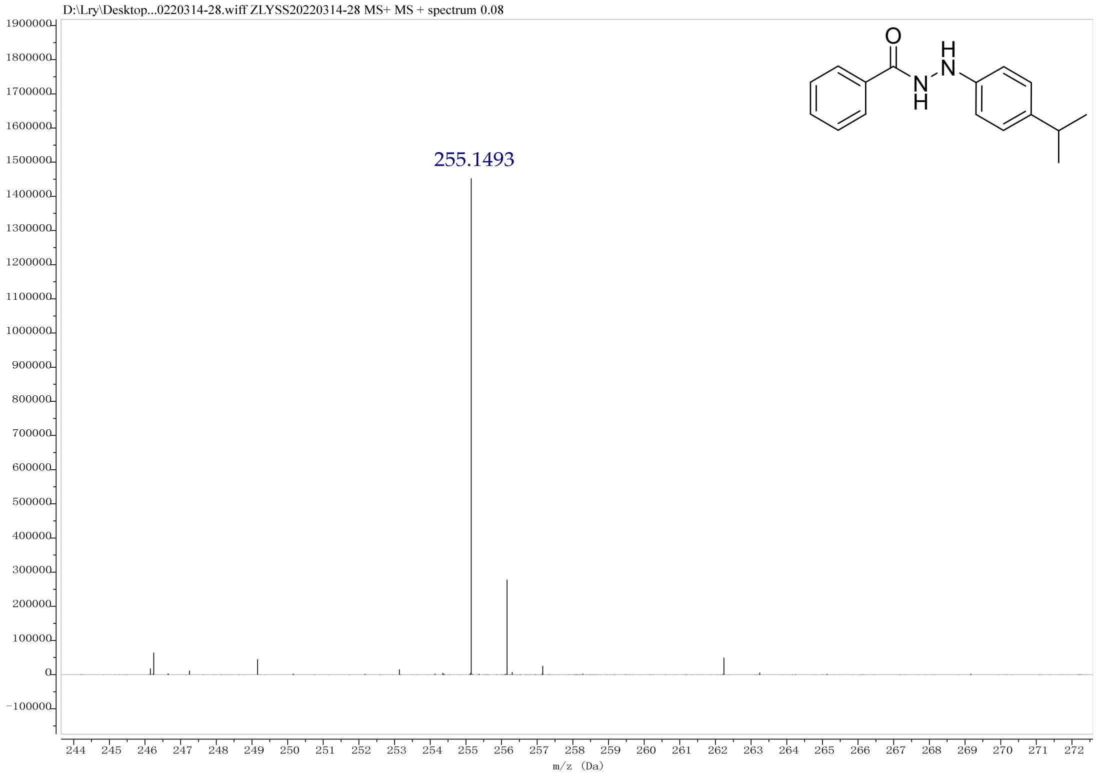

The HRMS spectrum of **B₁₃**.

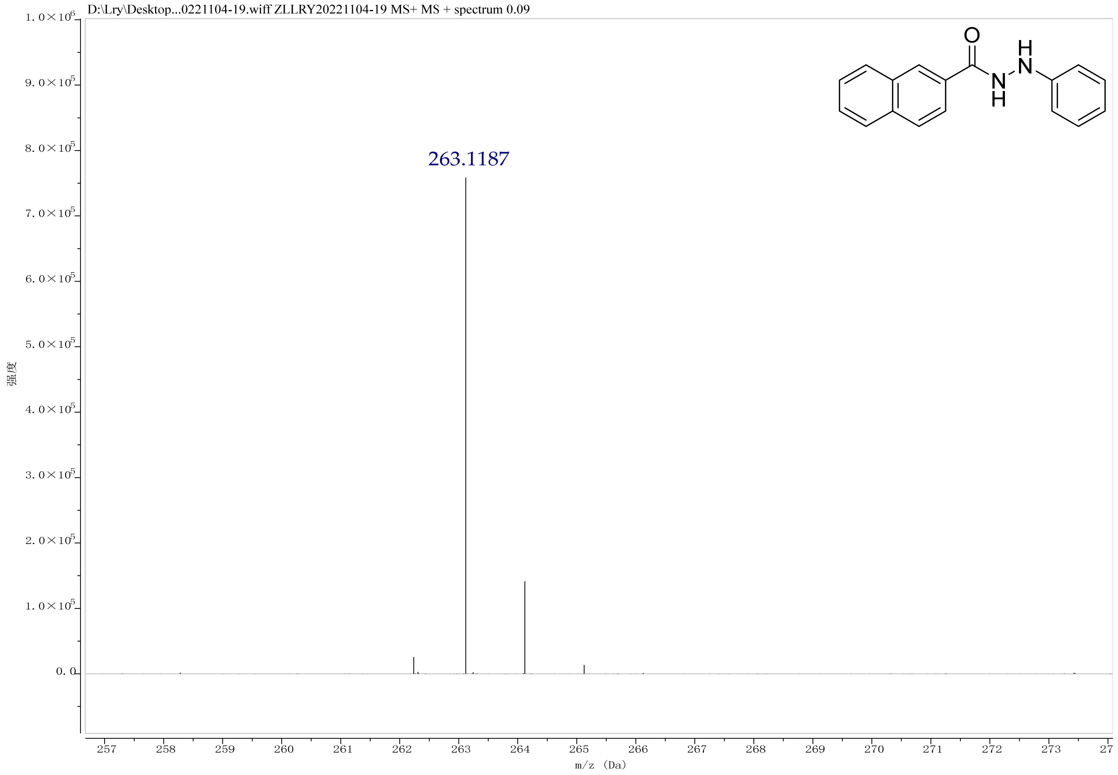

The HRMS spectrum of **C**6.

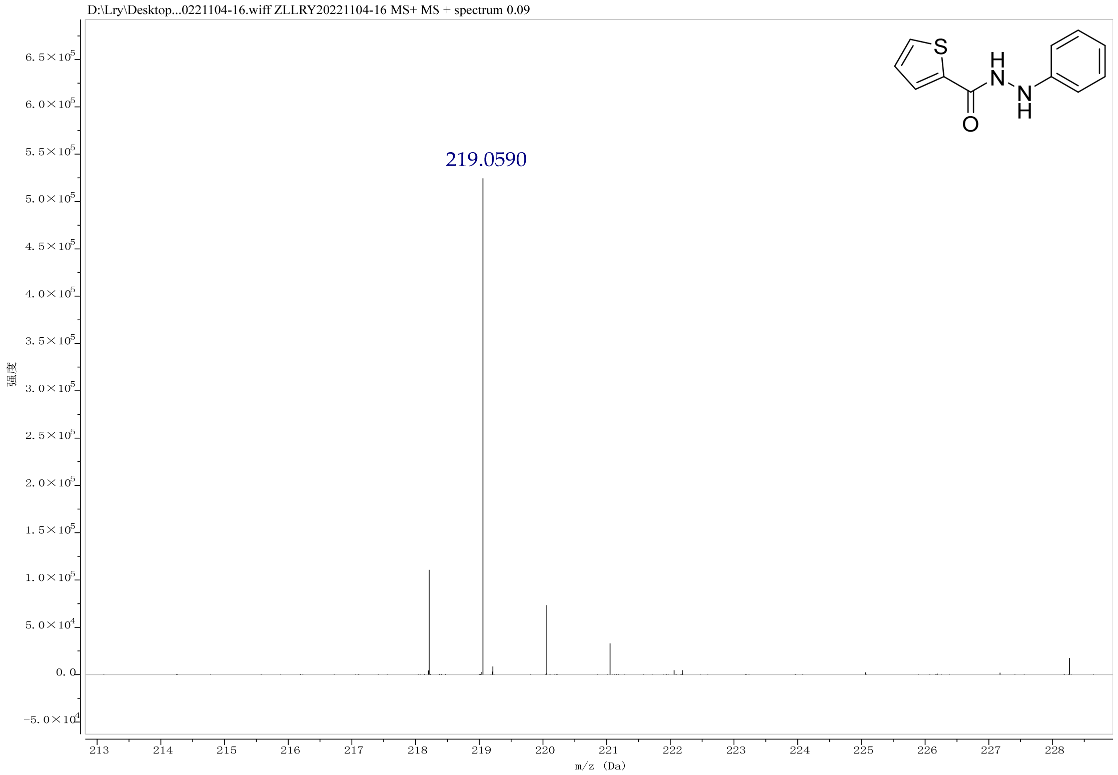

The HRMS spectrum of **C**7.

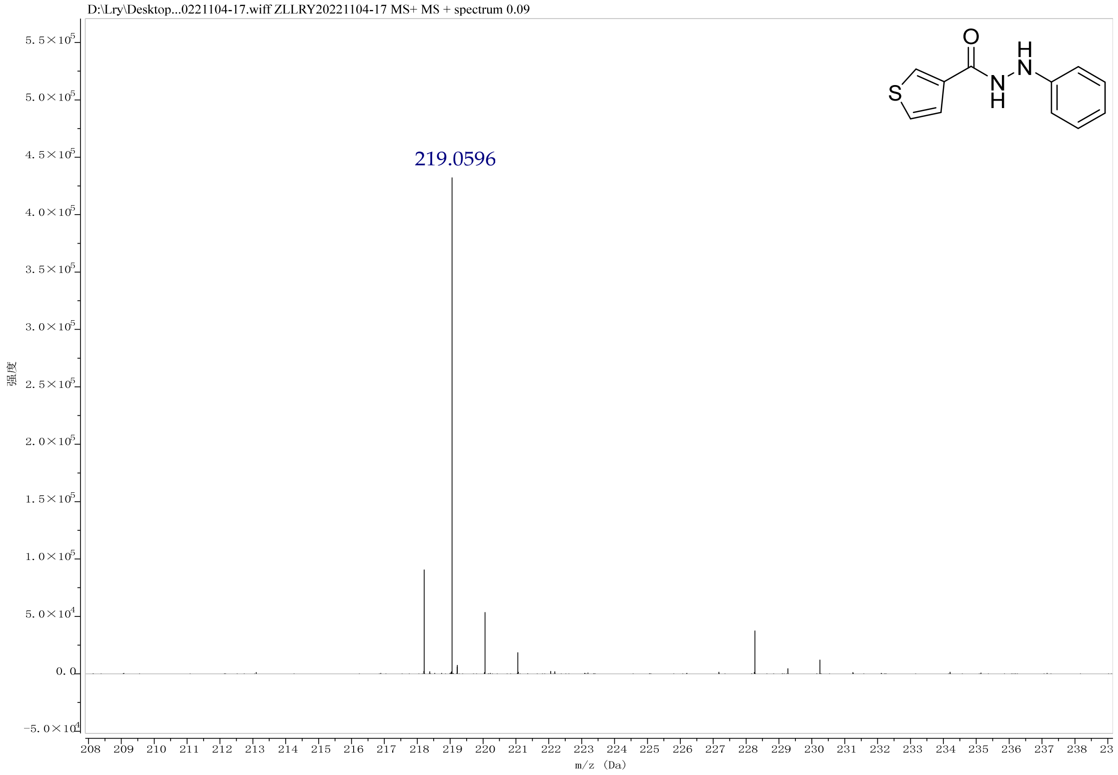

The HRMS spectrum of **C**8.

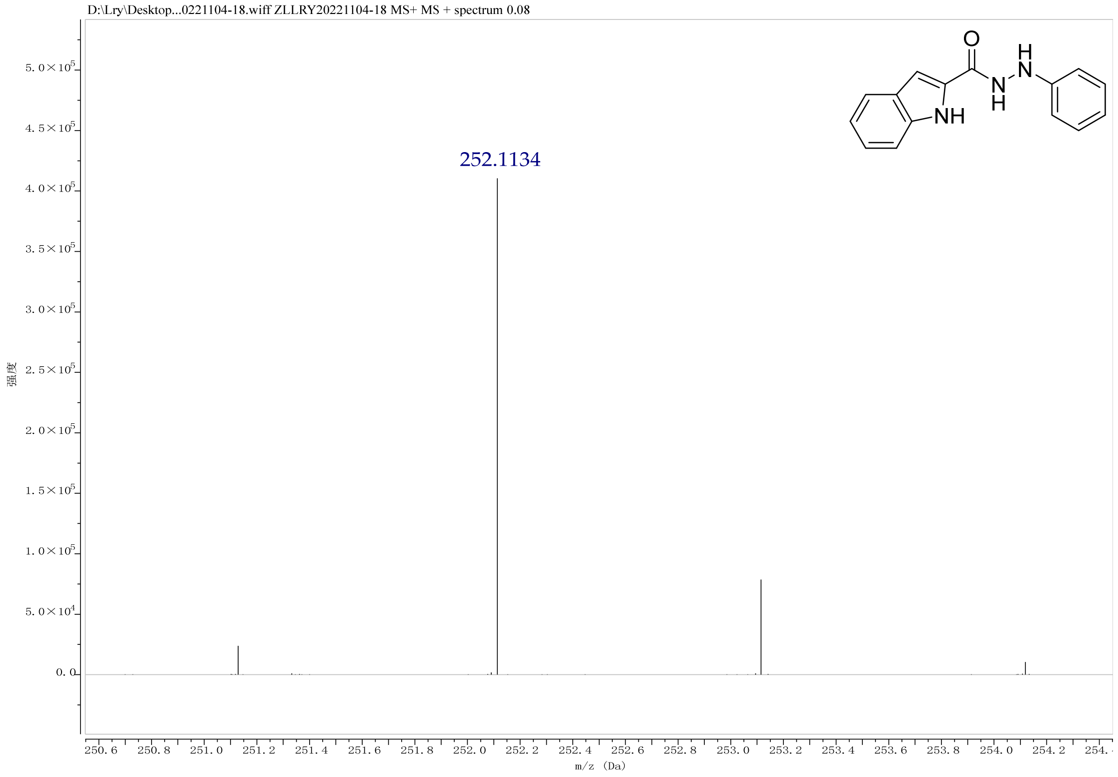

The HRMS spectrum of **C**9.

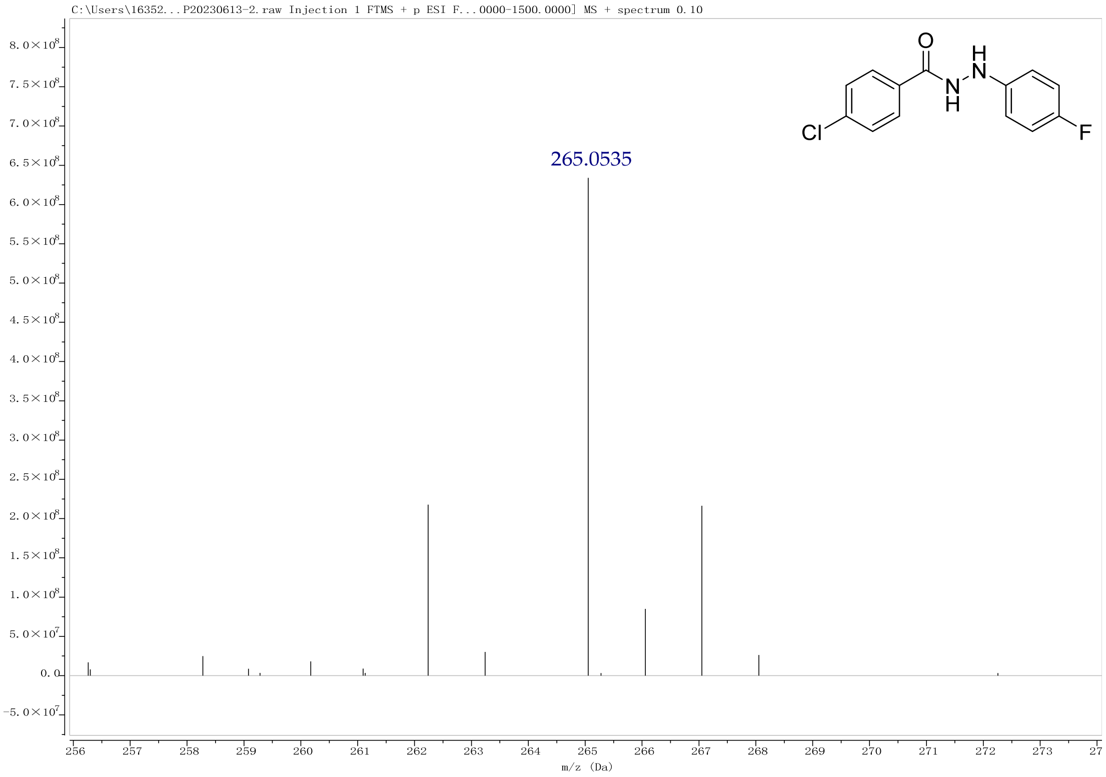

The HRMS spectrum of **D1**.

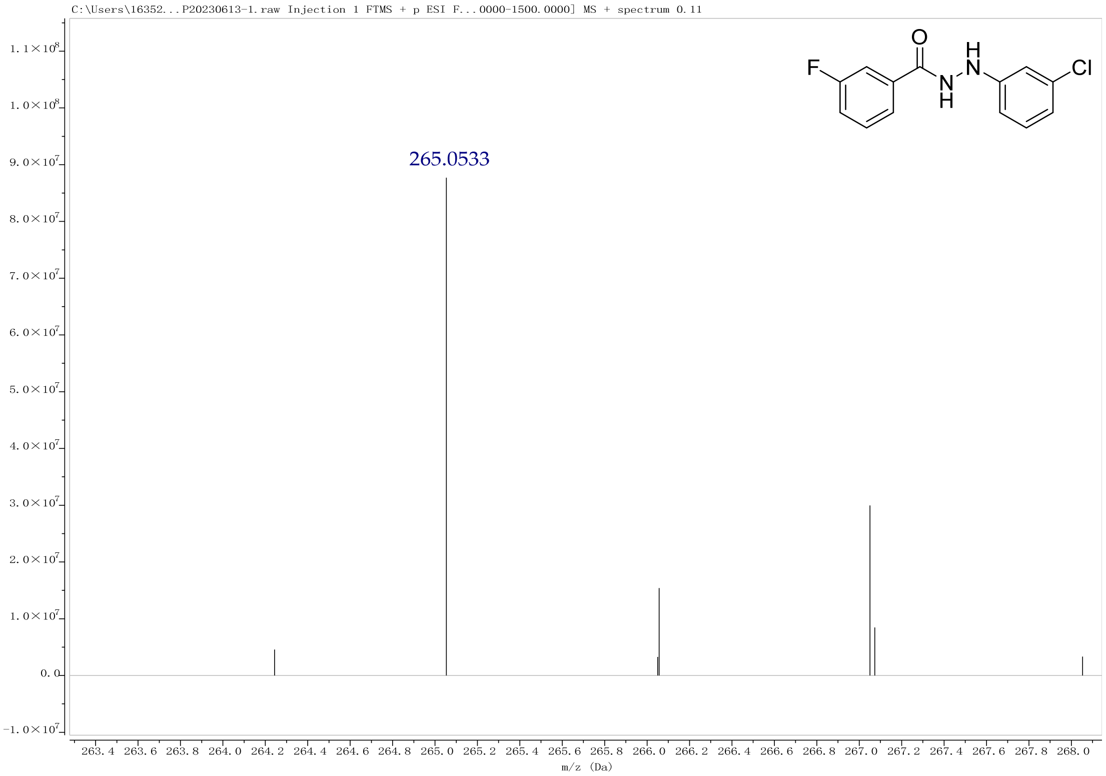

The HRMS spectrum of **D2**.

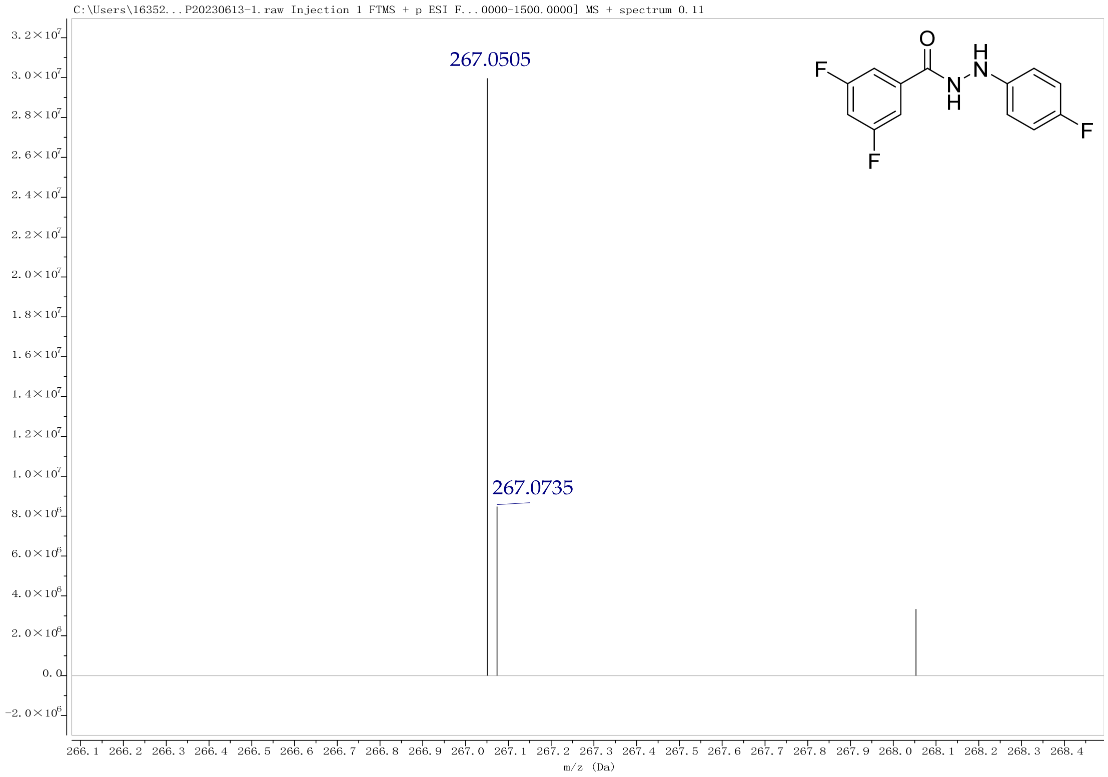

The HRMS spectrum of **D4**.

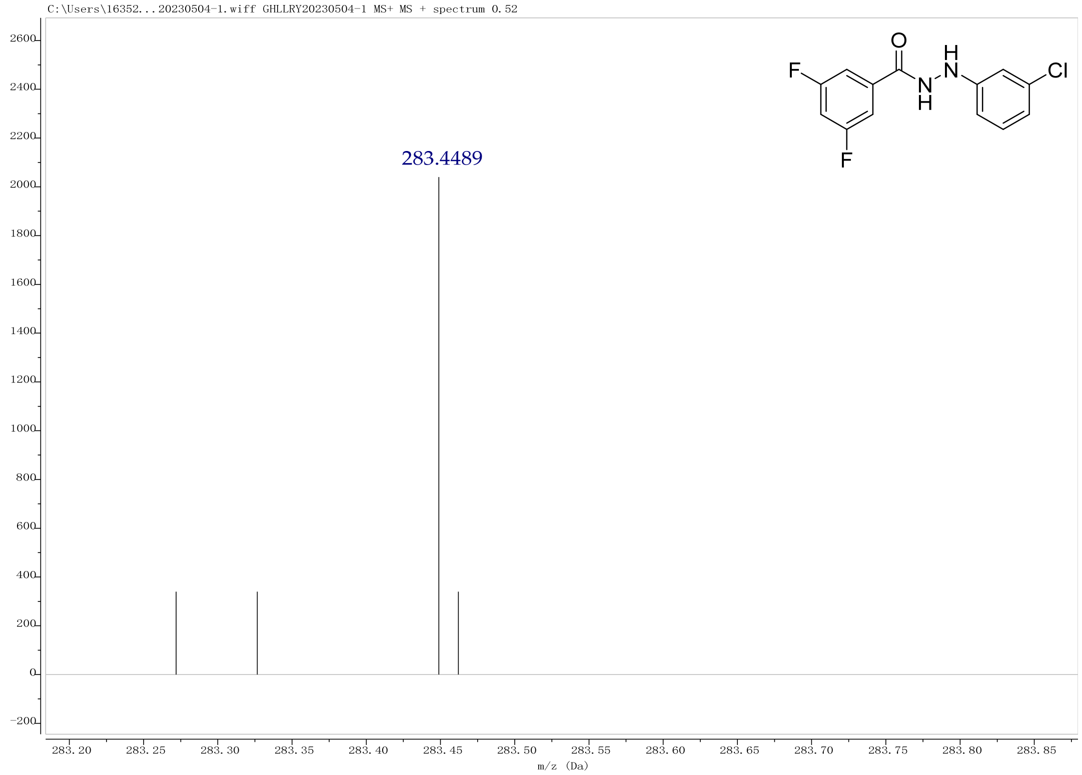

The HRMS spectrum of **D5**.

References

1. Zhang, J.Q.; Huang, G.B.; Weng, J.; Lu, G.; Chan, A.S.C. Copper(II)-Catalyzed Coupling Reaction: An Efficient and Regioselective Approach to N′,N′-Diaryl Acylhydrazines. *Org. Biomol. Chem.* **2015**, *13*, 2055–2063.
2. Huang, Z.Y.; Zhang, Q.Q.; Zhao, Q.G.; Yu, W.Q.; Chang, J.B. Synthesis of 2-Imino-1,3,4-Thiadiazoles from Hydrazides and Isothiocyanates via Sequential Oxidation and $P(NMe_2)_3$-Mediated Annulation Reactions. *Org. Lett.* **2020**, *22*, 4378–4382.
3. Shuler, W.G.; Smith, E.A.; Hess, S.M.; McFadden, T.M.C.; Metz, C.R.; Van Derveer, D.G.; Pennington, W.T.; Mabe, P.J.; Knick, S.L.; Beam, C.F. Preparation and X-Ray Crystal Structure of 3-(4-(Dimethylamino)Phenyl)-2-(Phenylamino)Isoquinolin-1(2h)-One, 3-(4-Methoxyphenyl)-2-(Phenylamino)Isoquinolin-1(2h)-One, and 2-Methyl-N′-(4-Methylbenzoyl)-N′-Phenylbenzohydrazide from Polylithiated 2-Methylbenzoic Acid Phenylhydrazide and Methyl 4-Dimethylaminobenzoate, Methyl 4-Methoxybenzoate, or Methyl 4-Methylbenzoate. *J. Chem. Crystallogr.* **2012**, *42*, 952–959.
4. Wang, T.T.; Gao, F.; Xue, M.; Song, Y.J.; Wang, W.H. Ethyl 3-(2-Chlorophenyl)-5-(Diethoxyphosphinoyl)-1-Phenyl-4,5-Dihydro-1h-Pyrazole-5-Carboxylate. *Acta Crystallogr. Sect. E Struct. Rep.* **2007**, *63*, o2549.
5. Liu, R.Y.; Li, Z.Z.; Liu, S.F.; Zheng, J.S.; Zhu, P.P.; Cheng, B.; Yu, R.J.; Geng, H.L. Synthesis, Structure–Activity Relationship, and Mechanism of a Series of Diarylhydrazide Compounds as Potential Antifungal Agents. *J. Agric. Food Chem.* **2023**, *71*, 6803–6817.
6. Liu, H.; Jia, H.; Wang, B.; Xiao, Y.; Guo, H. Synthesis of Spirobidihydropyrazole through Double 1,3-Dipolar Cycloaddition of Nitrilimines with Allenoates. *Org. Lett.* **2017**, *19*, 4714–4717.
7. Huang, Z.Y.; Zhang, Q.Q.; Yi, X.F.; Zhao, Z.X.; Yu, W.Q.; Chang, J.B. Synthesis of 2-Imino-1,3,4-Selenadiazoles via Tributylphosphine-Mediated Annulation of N-Aroyldiazenes with Isoselenocyanates. *Adv. Synth. Catal.* **2021**, *363*, 4894–4898.
8. Clements, J.S.; Islam, R.; Sun, B.; Tong, F.; Gross, A.D.; Bloomquist, J.R.; Carlier, P.R. N′-Mono- and N, N′-Diacyl Derivatives of Benzyl and Arylhydrazines as Contact Insecticides against Adult Anopheles Gambiae. Pestic. Biochem. Physiol. 2017, 143, 33–38.
9. Wang, W.J.; Zhang, T.; Duan, L.J.; Zhang, X.J.; Yan, M. KOt-Bu Promoted Homocoupling and Decomposition of N'-Aryl Acylhydrazines: Synthesis of Unsymmetric N′,N′-Diaryl Acylhydrazines. *Tetrahedron* **2015**, *71*, 9073–9080.
10. Sun, Y.; Ling, S.H.; Duan, Y.B.; Li, J.X.; Chen, Z.K.; Wu, X.F. Synthesis of 5-Trifluoromethyl-1,4-Dihydro-1,2,4-Triazines via Base-Mediated [3+3] Cycloaddition of Nitrile Imines and Cf3-Imidoyl Sulfoxonium Ylides. *Adv. Synth. Catal.* **2023**, *365*, 1521–1525.
11. Voronin, V.V.; Ledovskaya, M.S.; Gordeev, E.G.; Rodygin, K.S.; Ananikov, V.P. [3 + 2]-Cycloaddition of in Situ Generated Nitrile Imines and Acetylene for Assembling of 1,3-Disubstituted Pyrazoles with Quantitative Deuterium Labeling. *J. Org. Chem.* **2018**, *83*, 3819–3828.
12. Areephong, J.; Mattson, K.M.; Treat, N.J.; Poelma, S.O.; Kramer, J.W.; Sprafke, H.A.; Latimer, A.A.; Read de Alaniz, J.; Hawker, C.J. Triazine-Mediated Controlled Radical Polymerization: New Unimolecular Initiators. *Polym. Chem.* **2016**, *7*, 370–374.
13. Jimenez, A.X.; Palacios, F.; de los Santos, J.M. Sc(Otf)3-Mediated [4 + 2] Annulations of N-Carbonyl Aryldiazenes with Cyclopentadiene to Construct Cinnoline Derivatives: Azo-Povarov Reaction. *J. Org. Chem.* **2022**, *87*, 11583–11592.
14. Molina, C.L.; Chow, C.P.; Shea, K.J. Type 2 Intramolecular N-Acylazo Diels-Alder Reaction: Regio- and Stereoselective Synthesis of Bridgehead Bicyclic 1,2-Diazines. *J. Org. Chem.* **2007**, *72*, 6816–6823.
15. Hisler, K.; Commeureuc, A.G.J.; Zhou, S.Z.; Murphy, J.A. Synthesis of Indoles via Alkylidenation of Acyl Hydrazides. *Tetrahedron Lett.* **2009**, *50*, 3290–3293.
16. Yuan, C.; Ning, X.J.; Gao, T.; Zeng, Z.G.; Lee, K.; Xing, Y.L.; Sun, S.F.; Wang, G.Q. [3+2] Cycloaddition of Nitrile Imines with 3-Benzylidene Succinimides: A Facile Access to Functionalized Spiropyrazolines. *Asian J. Org. Chem.* **2022**, *11*, e202100699.
17. Sakamoto, T.; Kikugawa, Y. Synthesis of N-Phenylalkanehydrazonoyl Chlorides. *Chem. Pharm. Bull.* **1988**, *36*, 800–802.
18. Yamaguchi, J.I.; Aoyagi, T.; Fujikura, R.; Suyama, T. An Oxidative Transformation of N′-Phenylhydrazide to T-Butyl Ester Using a Copper (II) Halide-Lithium T-Butoxide System. *Chem. Lett.* **2001**, *30*, 466–467.

19. Lopez, R.R.; Romero, G.R.; Ortega, C.E.; Garrido, F.A. Dissipation Studies of Famoxadone in Vegetables under Greenhouse Conditions Using Liquid Chromatography Coupled to High-Resolution Mass Spectrometry: Putative Elucidation of a New Metabolite. *J. Sci. Food Agric.* **2019**, *99*, 5368–5376.
20. Kapkan, L.M.; Pekhtereva, T.M.; Chervinskii, A.Y.; Berdinskii, I.S. Conformation of Hydrazides. *Ukr. Khim. Zh. (Russ. Ed.)* **1989**, *55*, 404.
21. Murata, T.; Hara, S.; Niizuma, S.; Hada, K.; Kawada, H.; Sakaitani, M.; Shimada, H.; Nakanishi, Y. Preparation of Quinazolinone and Isoquinolinone Derivatives as Antitumor Agents for Treating Cancer and/or Cancer Metastasis and Invasion. WO2015060373, 24 December 2015.
22. Swindle, J.; Ajioka, J.; Hummel, H.S.; Robertson, S. Methods of Inhibiting Stearoyl Coa Desaturase. WO2009070533, 4 June 2009.
